# Genome-wide identification and expression profiling of *DnaJ* gene family in *Gossypium barbadense* reveals candidate thermotolerance genes

**DOI:** 10.3389/fpls.2025.1728216

**Published:** 2026-01-20

**Authors:** Ziling Han, Chao Li, ShuGuang Li, Jingchen Xu, Wenlong Li, Hemeng Wang, Yiling Liu, Yanqin Wang

**Affiliations:** Xinjiang Production & Construction Corps, Key Laboratory of Protection and Utilization of Biological Resources in Tarim Basin, College of Life Science and Technology, Tarim University, Alar, Xinjiang, China

**Keywords:** *DnaJ* gene family, heat stress, genome-wide identification, expression profiling, *Gossypium barbadense*

## Abstract

**Introduction:**

Heat stress is one of the primary abiotic stress factors affecting plant growth, seriously compromising crop quality and yield. The *DnaJ* gene family functions as a crucial component of molecular chaperones, playing a vital role in protein folding, unfolding, translocation, and degradation.

**Results:**

In this study, 109 *GbDnaJ* genes were identified and characterized in Gossypium barbadense, divided into five subfamilies based on the phylogeny analysis. Segmental duplication was identified as the primary driver for *GbDnaJ* family expansion, with the family underwent significant expansion and experiencing whole-genome duplication (WGD) events during the polyploidization process. The majority of duplicated gene pairs within Gossypium barbadense, as well as orthologous genes between related cotton species, were subjected to strong purifying selection. Cis-acting regulatory elements analysis revealed that the promoter regions of *GbDnaJ* genes are enriched with light-responsive, hormone-responsive and stress-responsive elements, and may be involved in cotton fiber development. Expression profiles demonstrated tissue-specific patterns for most *GbDnaJ* genes. Further investigation of 15 heat stress-responsive genes using RNA-seq data revealed divergent expression trends across tissues, with several genes showing strong stress-induced expression. These molecular patterns were closely associated with physiological changes, including decreased photosynthetic rate and increased activities of catalase and peroxidase.

**Discussion:**

This study provides the first comprehensive analysis of the evolutionary and functional characteristics of the *GbDnaJ* gene family, offering theoretical insights and candidate gene resources for elucidating *DnaJ*-mediated thermotolerance mechanisms in Gossypium barbadense.

## 1 Introduction

Global climate change poses an unprecedented threat to agricultural productivity and food security worldwide. In recent years, global temperatures have been progressively increasing. Studies have demonstrated that within a growing season, a 1°C rise in the average temperature can reduce crop yields by up to 17% (Urban et al., 2017). Elevated temperatures generally lead to decreased photosynthetic efficiency and a shortened growth period in most crops, ultimately resulting in diminished yield. Sea island cotton (*Gossypium barbadense*) is an important economic crop, widely valued for its superior fiber quality that makes it particularly suitable for high-end textile manufacturing and apparel production. However, *Gossypium barbadense* is relatively sensitive to heat stress during its growth and development. Heat stress occurring from mid-to-late July to early August can cause delayed vegetative growth, reduced boll size, increased boll shedding, and inferior fiber quality (Mittler et al., 2004). Therefore, identifying genes involved in the heat stress response is an effective strategy for enhancing thermotolerance and improving yield in *Gossypium barbadense*. However, little is known about the identification and function of the *DnaJ* gene family in *Gossypium barbadense*. In particular, the association of this gene family with fiber development is unknown.

Plants perceive and adapt to environmental changes through various physiological adjustments and molecular responses to enhance their resilience under extreme conditions. Among these mechanisms, Heat Shock Proteins (HSPs), a class of highly conserved and multifunctional molecular chaperones, play vital roles in maintaining protein homeostasis and enhancing stress resistance (Becker and Craig, 1994; Ahuja et al., 2010). Based on their molecular weights, HSPs in plants are primarily classified into six major families: HSP100, HSP90, HSP70, HSP60, HSP40, and sHSPs (HSP20) (Khan et al., 2021; Queitsch et al., 2000). HSP40, with an approximate molecular weight of 41 kDa, is referred to as DnaJ. DnaJ proteins typically contain a J-domain that interacts with the chaperone HSP70. By stimulating the ATPase activity of HSP70, they play crucial roles in protein folding, unfolding, translocation, synthesis, and degradation (Georgopoulos et al., 1980; Hartl, 1996; Qiu et al., 2006). Canonical DnaJ proteins are classified into four types based on their domain architectures: DnaJA, DnaJB, DnaJC, and DnaJD (Pulido and Leister, 2018), which are widely involved in plant responses to various abiotic stresses and pathogen infections (Qiu et al., 2006; Fu et al., 2025).

Numerous studies have reported the important roles of *DnaJ* genes in positively regulating plant drought and heat tolerance (Fan et al., 2017; Luo et al., 2019; Wang et al., 2019a). For instance, overexpression of *ZmDnaJ96* enhanced drought and heat tolerance in transgenic *Arabidopsis thaliana* by increasing antioxidant enzyme activities and osmolyte content, thereby maintaining membrane stability and reducing damage to chloroplasts under stress, ultimately improving root development and stress resistance (Cao et al., 2024). *LeCDJ1* protected chloroplasts and Photosystem II (PSII), effectively mitigating oxidative and physical damage caused by heat stress, thereby enhancing thermotolerance in tomato (Kong et al., 2014). Conversely, some studies have indicated that certain *DnaJ* proteins can negatively regulate temperature stress tolerance. For example, overexpression of *CmDnaJ27* in tobacco led to reactive oxygen species (ROS) accumulation and membrane lipid peroxidation, significantly reducing tolerance to both cold and heat stresses (Yu et al., 2025). Furthermore, some *DnaJ* genes are implicated in resistance to pathogen infection. For instance, *ClDJC24* negatively regulated citrus resistance to Huanglongbing (HLB) by suppressing key components of the salicylic acid signaling pathway and the expression of pathogenesis-related genes, thereby weakening the host’s defense against the pathogen (Tian et al., 2024). These findings collectively represent substantial progress in the research of plant *DnaJ* gene families.

However, while DnaJ proteins have been extensively studied in stress responses across various plant species, limited research is available on this gene family in *Gossypium barbadense*. Moreover, the functions of *DnaJ* genes in regulating heat stress tolerance and fiber development remain largely unknown, which constrains the understanding of the molecular mechanisms underlying heat adaptation and fiber quality determination in *Gossypium barbadense*. In this study, we performed the first comprehensive genome-wide identification and characterization of the *GbDnaJ* gene family, including phylogenetic relationships, gene structure, conserved motifs, cis-regulatory elements in promoter regions, and expression patterns under heat treatment, to elucidate their potential roles in thermotolerance and fiber quality regulation. These results provide a valuable resource for further investigation of the functions and molecular mechanisms of the *GbDnaJ* gene family.

## 2 Materials and methods

### 2.1 Plant materials and transcriptome sequencing analysis

This study utilized ‘Jinhai 1’, a high-quality *Gossypium barbadense* cultivar bred by Xinjiang Jinfengyuan Seed Co., Ltd., which is widely cultivated in Southern Xinjiang, as the experimental material. The field trial was conducted at the experimental station of Jinfengyuan Seed Co., Ltd. in Awat County, Xinjiang Uygur Autonomous Region. To effectively mitigate interference from drought, salinity, and other abiotic stresses with the experimental results, the following comprehensive management measures were implemented: the experimental field was selected in an area with uniform soil type and an appropriate groundwater level to ensure consistent background conditions across all plots. During the growth period, a drip irrigation system was used for controlled irrigation with freshwater that met agricultural water standards. Field management practices, including fertilization, intertillage, and pest control, were strictly standardized to prevent the effects of drought, secondary salinization, and biotic stress on the experiment.

Sampling was conducted during the flowering and boll-setting stage in late July. To control variables and minimize anthropogenic interference, sampling adhered to the following protocols: under heat stress conditions characterized by three consecutive days with average daily temperatures exceeding 40°C, control samples were collected at 9:00 AM on the fourth day (ambient temperature 22°C), while high-temperature treated samples were collected at 5:00 PM on the same day (ambient temperature 40°C). Detailed climate data on the sampling day are provided in **Supplementary Table 3**, which helped exclude potential interference from time and other confounding factors. Sampling was performed in the canopy zone, collecting flowers, buds, and leaves. Whole flowers and buds were collected with sepals removed and pooled separately. The third main-stem leaves from the apex were collected as fresh intact samples, with petioles removed after collection. Each treatment consisted of three biological replicates, with each replicate comprising samples pooled from five uniformly growing plants. Collected samples were immediately flash-frozen in liquid nitrogen and stored at -80°C for subsequent transcriptome sequencing, physiological and biochemical assays, and qRT-PCR analysis. All sampling and handling procedures followed strict RNase-free protocols to ensure RNA integrity.

RNA-seq raw reads were subjected to quality assessment using FastQC and were preprocessed with Trimmomatic to obtain high-quality clean reads. These clean reads were then aligned to the reference genome of *Gossypium barbadense* ‘3-79’ using HISAT2 to acquire their mapping positions. The aligned reads were assigned to genomic features using FeatureCounts to generate a raw count matrix. Differential expression analysis between the Heat and CK groups in each tissue was performed using both DESeq2 and edgeR. Genes with an adjusted p-value (padj) < 0.05 and an absolute log2 fold change (|log2FC|) > 2 were identified as differentially expressed genes (DEGs). For the visualization of expression patterns, the Transcripts Per Million (TPM) metric was used to quantify gene expression levels.

### 2.2 Identification and sequence analysis of the *DnaJ* gene family in *Gossypium barbadense*

The protein sequences of *Arabidopsis thaliana* DnaJ were obtained from the TAIR database(https://www.arabidopsis.org/) (Lamesch et al., 2012). The reference genome sequences, protein sequences and annotation files (GFF3 format) for the cotton species (AD2, ‘3-79’ genome HAU_v2_a1) (Wang et al., 2019b) were sourced from the Cotton Multi-omics Database (http://yanglab.hzau.edu.cn/CottonMD) (Yang et al., 2023). The Hidden Markov Model (HMM) profile for the conserved DnaJ domain (Pfam ID: PF00226) was downloaded from the Pfam database (http://pfam.xfam.org) and used as a query to identify potential protein sequences containing the DnaJ domain in *Gossypium barbadense* (Sesia et al., 2019). The Simple HMM Search function in TBtools (Chen et al., 2020)was employed to screen the *Gossypium barbadense* protein sequences using a strict E-value cutoff (e-value < 10^-20^), identifying candidate DnaJ protein sequences from *Gossypium barbadense*. To verify the accuracy of these candidate DnaJ proteins, redundant sequences were manually removed. The remaining sequences were further screened using the NCBI Conserved Domain Database (CDD) (Marchler-Bauer et al., 2015) (https://www.ncbi.nlm.nih.gov/Structure/cdd/cdd.shtml) based on protein conserved domains, confirming the final set of GbDnaJ protein sequences. The physicochemical properties of the GbDnaJ proteins were predicted using the TBtools Protein Parameter Calc function (Chen et al., 2020), including the number of amino acids, molecular weight, theoretical isoelectric point, instability index, aliphatic index, and grand average of hydropathicity. Subcellular localization predictions were performed using the online tool WOLF PSORT (https://wolfpsort.hgc.jp) (Horton et al., 2007).

### 2.3 Phylogenetic tree analysis of the *GbDnaJ* gene family

*Arabidopsis thaliana* DnaJ protein sequences were obtained from the TAIR database (https://www.arabidopsis.org/) (Lamesch et al., 2012). MAFFT software (v7.505) (Katoh, 2002)was used to perform multiple sequence comparisons of *Arabidopsis thaliana and Gossypium barbadense* DnaJ protein sequences, with local-pair algorithm and optimized 1,000 iterations. Parameter settings included a zero gap opening penalty to enhance alignment sensitivity. Phylogenetic tree were constructed using maximum likelihood (ML) method and Jones-Taylor-Thornton (JTT) model, and visualized with iTOL v7 (Letunic and Bork, 2007). Bootstrap tests with1000 replications were performed to ensure statistical reliability.

### 2.4 Chromosomal localization and gene duplication analysis of the *GbDnaJs*

The chromosomal localization of *GbDnaJ* genes was determined using the *Gossypium barbadense* genome sequence and annotation file (GFF). Chromosomal length and positional information for *GbDnaJ* family members were extracted using TBtools software (Chen et al., 2020). Synteny analysis was performed with the TBtools-MCScanX toolkit (Chen et al., 2020) to investigate segmental and tandem duplications resulting from gene duplication events. Selection pressure analysis within *Gossypium barbadense* and between *Gossypium barbadense* and its closely related *Gossypium* species was conducted using the TBtools-Simple Ka/Ks Calculator (NG) toolkit (Chen et al., 2020).

### 2.5 Analysis of protein motifs and gene structure of the *GbDnaJ* gene family

Conserved motifs were identified using the online MEME tool (version 5.5.2) (Bailey et al., 2009). The genomic annotation file (GFF) was utilized to extract gene structure information for the *GbDnaJ* genes, and the corresponding structural features were visualized using TBtools (Chen et al., 2020). The conserved protein motifs of *GbDnaJs* were analyzed via the MEME online website(https://meme-suite.org/meme/tools/meme) with the number of motifs set to 10 and other parameters kept at their default values. The motif distribution diagrams were generated using TBtools (Chen et al., 2020). Protein domain structures of the GbDnaJ sequences were analyzed using the NCBI CDD Batch CD-Search tool (https://www.ncbi.nlm.nih.gov/Structure/bwrpsb/bwrpsb.cgi) (Marchler-Bauer et al., 2015), and the protein domain architecture diagrams were subsequently plotted using TBtools (Chen et al., 2020).

### 2.6 Cis-acting regulatory elements analysis in the *GbDnaJ* gene family

The upstream regulatory regions (approximately 2000 bp) of the *GbDnaJ* gene sequences were extracted from the 3–79 genome and submitted to the PlantCARE database (Lescot, 2002) (https://bioinformatics.psb.ugent.be/webtools/plantcare/html/) to predict the distribution of cis-regulatory elements on the promoters. All identified cis-regulatory elements were subsequently extracted, categorized, and visualized using a custom script (**Supplementary Data 2**).

### 2.7 Interacting protein prediction of GbDnaJs and GbaHSP70s

The HSP70 protein sequences of *Gossypium barbadense* were derived from published research(**Supplementary Table 4**) (Rehman et al., 2020). Exploring potential interactions among GbDnaJs and GbaHSP70s proteins, we employed the STRING database (https://cn.string-db.org/) at a medium confidence score of 0.400. The results were visualized through Cytoscape software(v3.10.4), with node size and color employed to map node degree values for intuitive display of network topological features.

### 2.8 Expression of *GbDnaJ* genes based on RNA-seq in *Gossypium barbadense*

This study investigated the expression patterns of *GbDnaJ* genes in *Gossypium barbadense* using RNA-seq data from ‘Jinhai 1’, a widely cultivated cotton cultivar in Xinjiang. Gene expression levels were quantified and normalized using TPM values to enable comparison across samples and genes(**Supplementary Table 2**). A heat map of *GbDnaJ* genes under heat stress was generated using custom scripts, with hierarchical clustering performed to group genes with similar expression patterns (**Supplementary Data 1**).

### 2.9 RNA extraction and quantitative real-time PCR analysis

Total RNA was extracted from flowers, buds, and leaves of *Gossypium barbadense* using the RNAprep Pure Plant Plus Kit (for Polysaccharides & Polyphenolics-rich plant tissues) from Tiangen Biotech (Beijing, China), following the manufacturer’s instructions. The concentration and purity (A260/A280 ratio) of the RNA were measured using a NanoDrop spectrophotometer. Subsequently, cDNA was synthesized from the extracted RNA using the EasyScript^®^ One-Step gDNA Removal and cDNA Synthesis SuperMix (TransGen Biotech, China). Quantitative real-time PCR (qPCR) was performed using an Applied Biosystems real-time PCR system. Specific primers for the target genes were designed using NCBI Primer-BLAST (Ye et al., 2012) (https://www.ncbi.nlm.nih.gov/tools/primer-blast/index.cgi?LINK_LOC=BlastHome), detailed primer sequences are provided in **Supplementary Table 2**. The *ubq7* gene (encoding ubiquitin-40S ribosomal protein S27a) was used as the internal reference (Chen et al., 2019). The relative expression levels of the target genes were calculated using the 2^−ΔΔCT^ method (Livak and Schmittgen, 2001). All experiments included three independent biological replicates.

### 2.10 Analysis of physiological, biochemical and photosynthetic characteristics under heat stress

To assess the impact of heat stress on physiological activities in *Gossypium barbadense*, a series of key physiological and biochemical parameters were measured. We used a portable photosynthesis system (LI-6400/XT, LI-COR Biosciences) to determine net photosynthetic rate (Pn), stomatal conductance (Cond), intercellular CO_2_ concentration (Ci), and transpiration rate (Tr) between 10:00 and 17:00. Activities of antioxidant enzymes and oxidative stress markers, including superoxide dismutase (SOD, BC1075), peroxidase (POD, BC0095), ascorbate peroxidase (APX, BC0225), and proline (Pro, BC0295) content, were analyzed using commercial assay kits (Solarbio, Beijing, China) according to the manufacturer’s protocols. Samples from flowers, buds, and leaves were collected following the same procedures used for transcriptome sequencing and qRT-PCR validation. All measurements were performed with three biological and technical replicates.

## 3 Results

### 3.1 Identification and characterization of *DnaJ* genes in *Gossypium barbadense*

**Figure 1:**
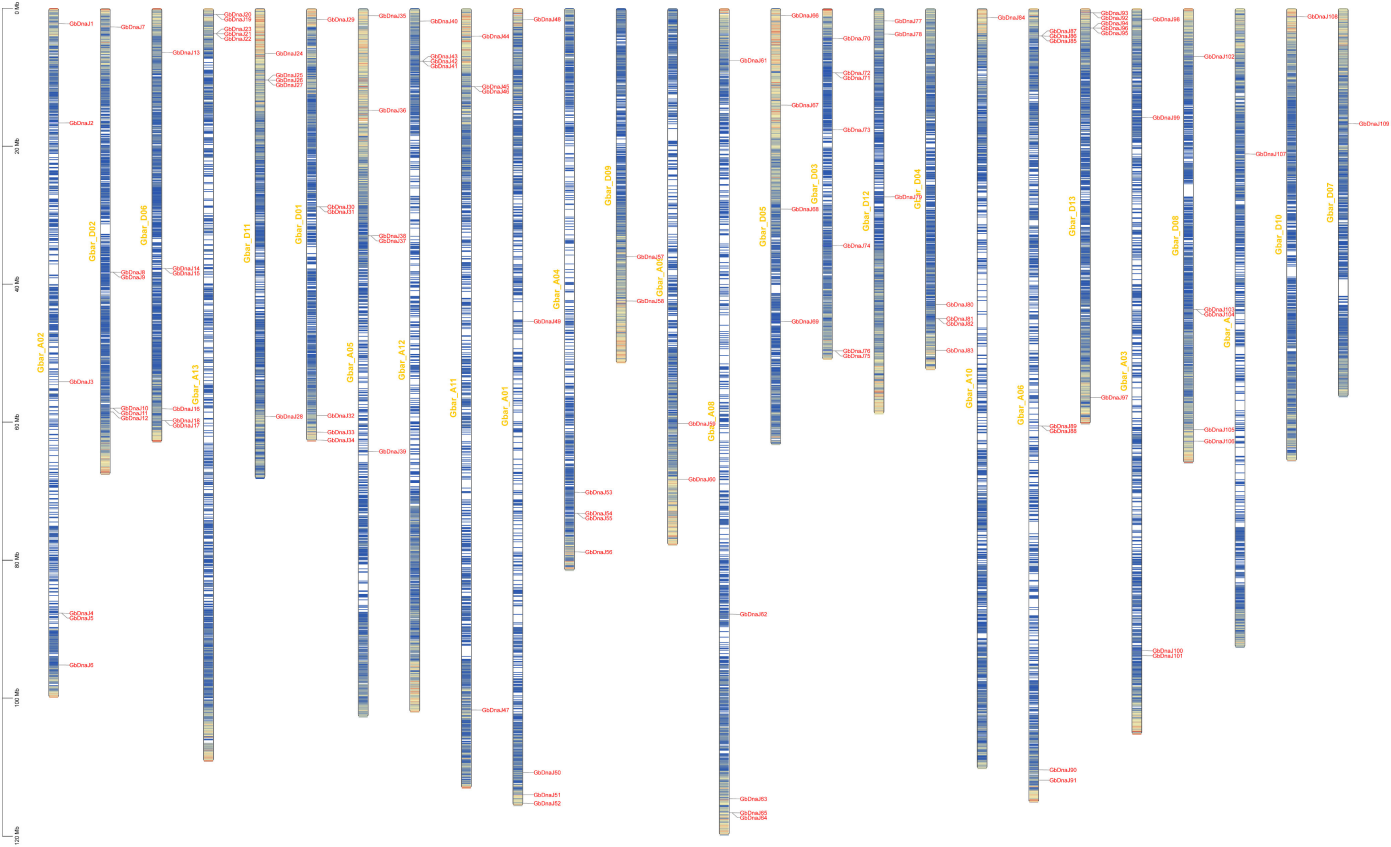
Chromosomal localization of *GbDnaJ* genes.

A total of 109 *GbDnaJ* genes were identified in the *Gossypium barbadense* genome using the Hidden Markov Model (HMM) profile of the *DnaJ* structural domain (PF00226). The presence of a complete *DnaJ* domain in all members was confirmed based on the Pfam and NCBI CDD databases. These genes were systematically named from *GbDnaJ1* to *GbDnaJ109* according to their chromosomal positions (**Figure 1**).

The physicochemical properties of the corresponding proteins were further analyzed, including protein length, molecular weight (MW), theoretical isoelectric point (pI), and predicted subcellular localization. The protein lengths ranged from 103 to 541 amino acids (aa), and the molecular weights varied between 12.47 and 59.92 kDa. The theoretical pI values spanned from 4.66 to 9.79, reflecting a broad distribution of protein charge. Subcellular localization predictions indicated that most GbDnaJ proteins are localized in the nucleus, cytoplasm, and chloroplasts (**Table 1**). This diverse subcellular distribution suggests that the *GbDnaJ* gene family may play various functional roles in different tissues and cellular compartments.

**Table 1:** Physicochemical properties and subcellular localization of GbDnaJ proteins.

Gene name	Gene ID	Protein length (aa)	Molecular weight	PI	Instability index	Aliphatic index	Hydropathicity	Subcellular localization
Gbar_A02G001960.1	GbDnaJ1	189	22558.5	9.79	68.53	63.07	-1.026	nucl
Gbar_A02G008020.3	GbDnaJ2	133	15794.61	4.66	57.28	78.42	-0.823	nucl
Gbar_A02G011450.1	GbDnaJ3	385	43427.77	5.46	39.92	78.83	-0.593	cyto
Gbar_A02G013750.2	GbDnaJ4	109	12694.21	5.31	19.5	77.89	-0.721	cysk
Gbar_A02G013750.1	GbDnaJ5	124	14501.08	4.84	22.05	72.42	-0.822	cysk
Gbar_A02G015410.1	GbDnaJ6	446	48064.79	9.39	43.31	74.93	-0.338	chlo
Gbar_D02G002390.1	GbDnaJ7	189	22389.36	9.59	58.18	65.13	-0.949	nucl
Gbar_D02G012940.4	GbDnaJ8	425	46465.65	8.96	33.9	72.24	-0.344	chlo
Gbar_D02G012940.3	GbDnaJ9	408	44505.43	9.07	34.81	70.96	-0.388	chlo
Gbar_D02G017820.2	GbDnaJ10	411	46797.04	7.61	45.05	71.9	-0.615	nucl
Gbar_D02G017820.1	GbDnaJ11	395	45006	8.08	47.27	71.85	-0.642	nucl
Gbar_D02G018080.1	GbDnaJ12	287	32080.77	9.3	41.59	84.6	-0.571	cyto
Gbar_D06G004970.1	GbDnaJ13	336	36855.25	8.62	38.25	64.73	-0.765	cyto
Gbar_D06G013330.2	GbDnaJ14	507	54073.84	9.04	36.27	68.48	-0.39	chlo
Gbar_D06G013330.1	GbDnaJ15	468	50039.32	8.98	41.51	68.12	-0.356	chlo
Gbar_D06G019300.2	GbDnaJ16	418	46530.59	6.06	35.02	62.75	-0.756	nucl
Gbar_D06G020350.1	GbDnaJ17	385	42612.15	5.78	47.48	77.38	-0.502	cyto
Gbar_D06G020350.2	GbDnaJ18	394	43692.46	6.13	46.62	77.34	-0.519	cyto
Gbar_A13G000950.2	GbDnaJ19	298	33449.04	5.1	36.99	87.48	-0.334	cyto
Gbar_A13G000950.1	GbDnaJ20	399	44539.99	5.21	43.05	77.84	-0.518	cyto
Gbar_A13G003220.3	GbDnaJ21	320	35981.15	5.97	28.76	84.75	-0.443	cyto
Gbar_A13G003220.1	GbDnaJ22	229	25924.44	5.9	22.11	77.12	-0.528	cyto
Gbar_A13G003220.2	GbDnaJ23	333	37619.99	6.29	30	84.65	-0.486	cyto
Gbar_D11G008390.2	GbDnaJ24	423	47431.54	7.23	38.94	59.22	-0.85	nucl
Gbar_D11G012080.1	GbDnaJ25	344	38816.2	6.19	37.74	80.17	-0.52	cyto
Gbar_D11G012080.2	GbDnaJ26	370	41818.83	6.8	38.89	80.59	-0.435	chlo
Gbar_D11G012090.1	GbDnaJ27	344	38742.12	6.19	38.21	80.76	-0.506	cyto
Gbar_D11G029420.1	GbDnaJ28	158	18005.01	5.11	48.47	48.86	-0.82	chlo
Gbar_D01G001910.1	GbDnaJ29	348	38618.2	8.66	32.76	78.99	-0.407	cysk
Gbar_D01G013360.2	GbDnaJ30	290	33411.53	8.64	49.31	57.55	-1.164	nucl
Gbar_D01G013360.1	GbDnaJ31	287	33070.11	8.16	48.88	58.15	-1.146	nucl
Gbar_D01G020500.1	GbDnaJ32	286	32744.83	6.98	49.73	64.48	-0.973	nucl
Gbar_D01G022320.1	GbDnaJ33	490	54601.33	7.19	41.16	88.1	-0.379	chlo
Gbar_D01G023610.1	GbDnaJ34	339	37469.81	9.22	35.57	66.73	-0.623	cyto
Gbar_A05G001160.1	GbDnaJ35	337	37265.17	8.97	36.28	69.41	-0.71	cyto
Gbar_A05G015830.1	GbDnaJ36	344	38047.38	9.22	37.17	68.9	-0.642	cyto
Gbar_A05G028270.2	GbDnaJ37	340	37557.71	8.8	29.71	62.21	-0.763	nucl
Gbar_A05G028270.5	GbDnaJ38	418	46464.55	6.06	36.63	62.99	-0.747	nucl
Gbar_A05G032380.1	GbDnaJ39	406	45303.91	5.86	36.28	71.11	-0.566	nucl
Gbar_A12G001440.1	GbDnaJ40	435	47419.12	9.1	35.53	79.77	-0.363	chlo
Gbar_A12G004210.3	GbDnaJ41	326	36811.08	5.5	30.86	85.61	-0.453	cyto
Gbar_A12G004210.1	GbDnaJ42	331	37314.85	6.29	30.23	86.1	-0.44	cyto
Gbar_A12G004210.2	GbDnaJ43	298	33719.44	5.88	31.49	80.57	-0.56	nucl
Gbar_A11G004790.3	GbDnaJ44	422	47215.33	7.23	36.9	61	-0.813	nucl
Gbar_A11G011530.1	GbDnaJ45	344	38750.14	6.24	38.24	81.6	-0.513	cyto
Gbar_A11G011540.1	GbDnaJ46	344	38741.04	6.11	36.19	79.33	-0.522	vacu
Gbar_A11G028930.1	GbDnaJ47	158	18067.08	4.98	48.98	45.76	-0.822	chlo
Gbar_A01G001780.1	GbDnaJ48	348	38561.03	8.88	34.58	78.99	-0.409	cyto
Gbar_A01G012710.1	GbDnaJ49	287	33178.17	8.16	50.31	56.79	-1.184	nucl
Gbar_A01G019400.1	GbDnaJ50	280	32118.12	6.93	47.71	64.11	-1.009	nucl
Gbar_A01G021240.1	GbDnaJ51	492	54655.14	6.39	41.65	87.54	-0.38	chlo
Gbar_A01G022540.1	GbDnaJ52	340	37322.55	9.23	35.33	64.53	-0.648	cyto
Gbar_A04G009610.3	GbDnaJ53	343	37808.18	9.1	39.98	62.83	-0.652	nucl
Gbar_A04G010450.1	GbDnaJ54	409	46214.49	8.02	40.49	73.5	-0.593	nucl
Gbar_A04G010450.2	GbDnaJ55	332	37043.03	5.47	41.11	79.07	-0.346	nucl
Gbar_A04G013400.1	GbDnaJ56	338	38084.25	5.42	37.45	86.63	-0.48	nucl
Gbar_D09G010880.1	GbDnaJ57	417	46231.23	5.68	38.59	64.53	-0.729	nucl
Gbar_D09G015870.1	GbDnaJ58	482	53711.34	6.85	39.55	89.54	-0.35	chlo
Gbar_A09G011130.1	GbDnaJ59	417	46275.33	5.78	39.64	64.53	-0.731	nucl
Gbar_A09G016160.1	GbDnaJ60	480	53224.6	6.85	42.3	86.67	-0.374	E.R.
Gbar_A08G005860.1	GbDnaJ61	444	48403.9	9.04	37.27	73.94	-0.434	mito
Gbar_A08G013140.1	GbDnaJ62	342	38899.2	6.4	40.7	74.94	-0.628	cyto
Gbar_A08G022220.3	GbDnaJ63	151	17031.95	4.87	36.09	54.3	-0.641	nucl
Gbar_A08G023970.3	GbDnaJ64	103	12471.26	6.91	26.99	88.93	-0.667	cyto
Gbar_A08G023970.2	GbDnaJ65	126	14900.7	5.37	19.63	76.67	-0.866	cyto
Gbar_D05G001160.1	GbDnaJ66	313	34564.9	7.72	34.31	66.65	-0.722	cyto
Gbar_D05G016250.1	GbDnaJ67	344	37960.17	9.14	37.57	66.92	-0.667	cyto
Gbar_D05G029160.2	GbDnaJ68	418	46550.62	6.06	35.63	62.06	-0.767	nucl
Gbar_D05G033450.1	GbDnaJ69	406	45358.05	6	37.13	72.07	-0.551	nucl
Gbar_D03G003760.2	GbDnaJ70	443	47892.61	9.44	44.63	75.67	-0.353	chlo
Gbar_D03G005380.2	GbDnaJ71	110	12880.43	5.31	18.04	77.18	-0.723	cysk
Gbar_D03G005380.1	GbDnaJ72	124	14501.08	4.84	22.05	72.42	-0.822	cysk
Gbar_D03G006970.1	GbDnaJ73	376	42802.59	7.19	38.86	81.76	-0.555	cyto
Gbar_D03G009490.1	GbDnaJ74	354	39826.97	9.15	44.73	74.12	-0.751	cyto
Gbar_D03G017140.1	GbDnaJ75	541	59924.37	8.86	31.2	86.75	-0.379	chlo
Gbar_D03G017140.2	GbDnaJ76	429	47442.2	9.19	34.17	88.02	-0.267	golg
Gbar_D12G001600.1	GbDnaJ77	435	47475.22	9.27	36.37	79.31	-0.381	chlo
Gbar_D12G003030.1	GbDnaJ78	347	38995.55	6.44	30.73	85.24	-0.506	chlo
Gbar_D12G009320.2	GbDnaJ79	443	47687.43	9.21	46.26	73.68	-0.295	chlo
Gbar_D04G014220.2	GbDnaJ80	343	37664.06	9.3	44.51	65.98	-0.627	cyto
Gbar_D04G015100.2	GbDnaJ81	333	37143.15	5.37	38.42	79.7	-0.333	nucl
Gbar_D04G015100.1	GbDnaJ82	410	46341.68	8.3	37.98	74.02	-0.584	nucl
Gbar_D04G018060.1	GbDnaJ83	338	38125.35	5.99	38.31	85.18	-0.491	cyto
Gbar_A10G001710.1	GbDnaJ84	300	35371.72	9.24	47.69	91.4	-0.512	plas
Gbar_A06G003250.1	GbDnaJ85	447	49659.02	7.9	39.95	67.67	-0.486	mito
Gbar_A06G003250.6	GbDnaJ86	448	49845.23	7.9	39.65	67.52	-0.487	mito
Gbar_A06G003250.4	GbDnaJ87	456	50736.22	7.9	39.64	68.05	-0.484	mito
Gbar_A06G012350.2	GbDnaJ88	507	54190.95	8.91	35.92	68.11	-0.408	chlo
Gbar_A06G012350.1	GbDnaJ89	468	50130.43	8.91	40.03	68.33	-0.356	chlo
Gbar_A06G018580.1	GbDnaJ90	418	46475.58	6.25	32.99	64.14	-0.741	nucl
Gbar_A06G019490.1	GbDnaJ91	394	43669.38	5.91	46.06	77.34	-0.519	cyto
Gbar_D13G000860.1	GbDnaJ92	400	44666.15	5.34	45.15	77.15	-0.528	cyto
Gbar_D13G000860.2	GbDnaJ93	298	33458.09	5.25	36.79	88.12	-0.316	cyto
Gbar_D13G003020.3	GbDnaJ94	326	36601.78	5.55	30.82	84.69	-0.446	chlo
Gbar_D13G003020.2	GbDnaJ95	333	37649.04	5.99	29.82	85.83	-0.484	chlo
Gbar_D13G003020.1	GbDnaJ96	296	33569.43	5.96	28.39	81.08	-0.493	chlo
Gbar_D13G022000.2	GbDnaJ97	375	41736.89	8.87	55.37	65.89	-0.599	nucl
Gbar_A03G001380.1	GbDnaJ98	541	59881.33	8.75	31.84	88.54	-0.367	chlo
Gbar_A03G007570.1	GbDnaJ99	354	39877.01	9.05	43.25	73.31	-0.763	cyto
Gbar_A03G015990.2	GbDnaJ100	411	46751.93	7.09	43.03	70	-0.637	nucl
Gbar_A03G016260.1	GbDnaJ101	287	32049.76	9.37	42.71	84.6	-0.569	cyto
Gbar_D08G006110.1	GbDnaJ102	444	48404.97	9.04	36.57	75.92	-0.405	mito
Gbar_D08G013810.1	GbDnaJ103	342	38848.3	6.54	39.15	76.37	-0.586	cyto
Gbar_D08G013810.2	GbDnaJ104	271	30719.13	5.23	39.35	80.96	-0.362	chlo
Gbar_D08G022940.1	GbDnaJ105	151	16921.86	4.89	28.28	61.39	-0.571	chlo
Gbar_D08G024590.1	GbDnaJ106	126	14884.66	5.37	19.03	73.57	-0.909	cyto
Gbar_A07G011900.1	GbDnaJ107	416	46313.4	6.05	33.54	66.8	-0.7	nucl
Gbar_D10G001630.1	GbDnaJ108	300	35329.64	9.18	47.98	91.07	-0.503	plas
Gbar_D07G012290.1	GbDnaJ109	416	46199.26	5.87	34.19	66.35	-0.684	nucl

The table includes gene name(original gene name), gene id(renamed gene name), protein length(aa), molecular weight, theoretical isoelectric point(PI), instability index, aliphatic index, Hydropa thicity, Subcellular localization.

### 3.2 Phylogenetic analysis of the *GbDnaJ* gene family

**Figure 2:**
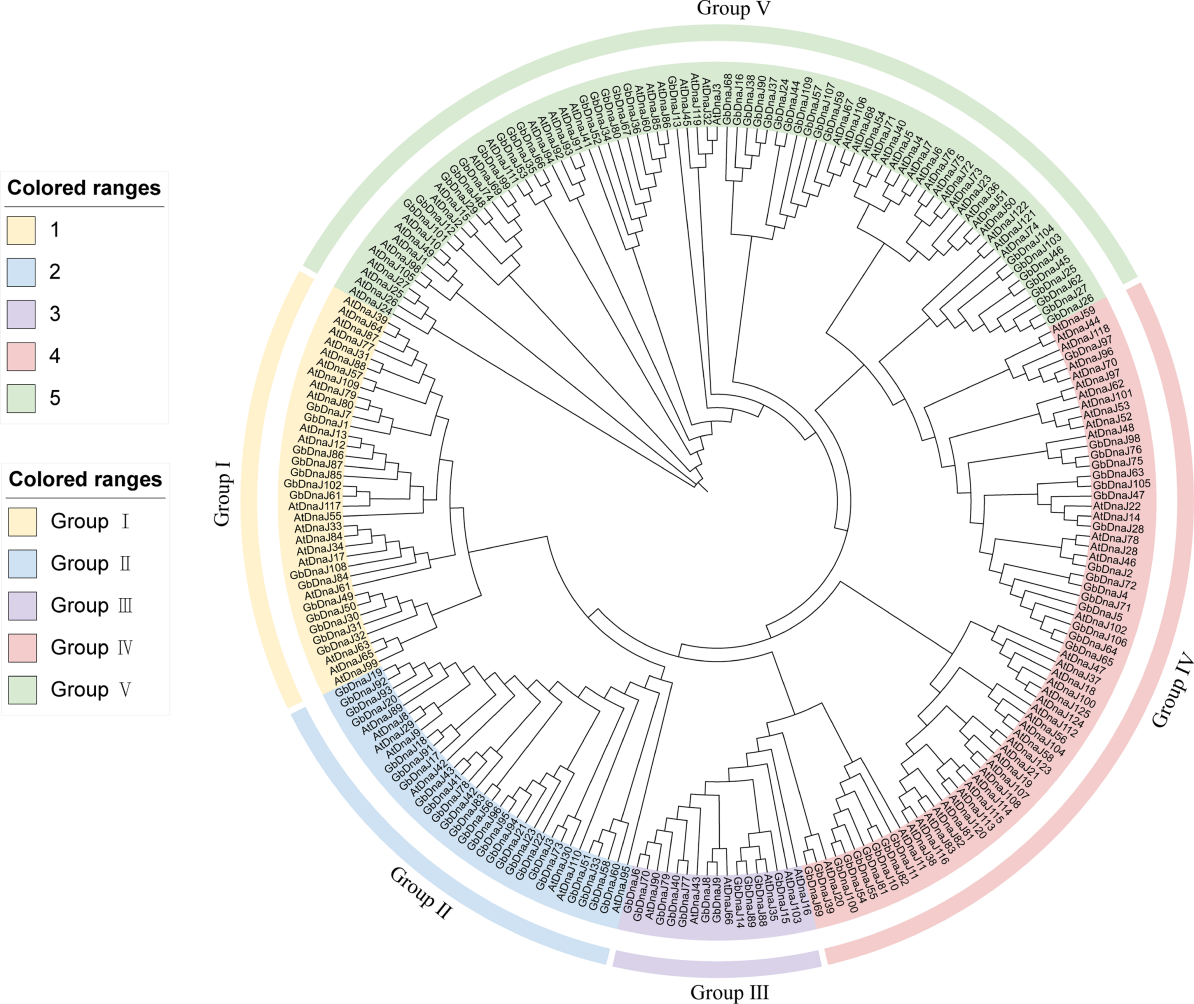
Phylogenetic tree of 109 GbDnaJ and 125 AtDnaJ proteins. Distinct background colors represent different phylogenetic types (Type I-V). The tree was constructed using the maximum likelihood method, illustrating the evolutionary relationships and classification of DnaJ proteins between *Gossypium barbadense* and *Arabidopsis thaliana*. Major clades are highlighted with colored backgrounds corresponding to the five phylogenetic groups.

To understand the phylogenetic relationships of the *GbDnaJ* gene family, the maximum likelihood (ML) phylogenetic tree was constructed using the protein sequences of 109 GbDnaJ and 125 *Arabidopsis thaliana* AtDnaJ proteins(**Figure 2**). The phylogenetic tree revealed that the GbDnaJ members could be classified into five distinct types: Type I, Type II, Type III, Type IV, and Type V. Type I comprised 14 GbDnaJs, Type II contained 25 GbDnaJs, Type III included 11 GbDnaJs, and Type IV consisted of 25 GbDnaJs. The remaining members clustered into Type V, forming the largest group with a total of 34 GbDnaJs.

The phylogenetic analysis indicated that the *DnaJ* gene family has undergone extensive expansion during the evolution of *Arabidopsis thaliana* and *Gossypium barbadense*. This expansion includes both ancient orthologous gene pairs and numerous recent lineage-specific gene duplication events. Some *GbDnaJ* genes clustered within the same clades as *AtDnaJ* genes, suggesting they originate from common ancestral genes and may retain conserved molecular functions. Conversely, the presence of multiple paralogous gene clusters in *Gossypium barbadense*, formed through species-specific duplications, is potentially associated with its unique genomic evolutionary history and environmental adaptation. Certain clades exhibited relatively long branch lengths, indicating that these genes may have undergone strong positive selection or functional diversification. This phylogenetic analysis demonstrates that the *DnaJ* families in *Gossypium barbadense* and *Arabidopsis thaliana* exhibit both functional conservation and functional diversity resulting from species-specific evolution. These findings provide a foundation for subsequent investigations into the roles of *GbDnaJ* genes in the response of *Gossypium barbadense* to abiotic stress.

### 3.3 Chromosomal distribution and gene duplication of *GbDnaJ* genes

**Figure 3:**
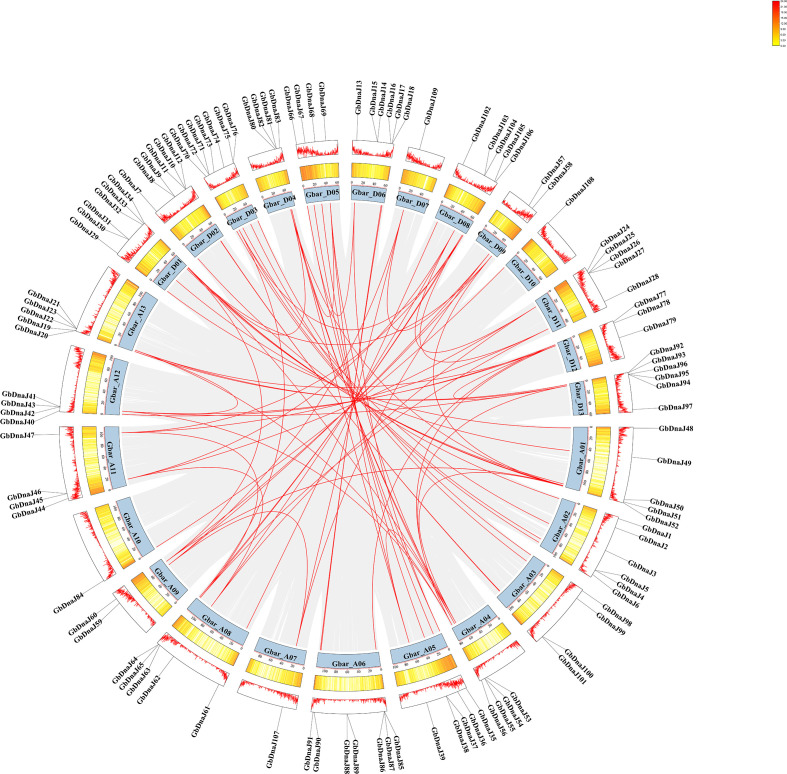
Chromosomal locations and synteny of *GbDnaJ* genes. The innermost circle labeled from Gbar_A01 to Gbar_A13 and Gbar_D01 to Gbar_D13 represents the 26 chromosomes of *Gossypium barbadense*. The numeric scale 0–100 represents the distance of genes along each chromosomes. The colored scale within the circle represents the density of gene distribution across maize chromosomes, with red and yellow indicating the highest and lowest gene densities. This density is also represented by a red line graph, where peak height corresponds to regions of high gene density. The outermost circle is the distribution of 109 *GbDnaJ* genes on chromosomes. Within the central area, the grey lines illustrate gene duplications throughout the maize genome, while the red lines specifically highlight the duplications among the 109 *GbDnaJ* genes.

The 109 *GbDnaJ* genes were unevenly distributed across all 26 chromosomes of *Gossypium barbadense*, with each chromosome containing between one and seventeen genes. Chromosomes A06 and D03 harbored the highest number of genes (7 each), suggesting that these chromosomal regions may have undergone gene duplication events, leading to family expansion. In contrast, chromosomes A07, A10, D07, and D10 contained only a single gene each, representing the lowest densities. Most other chromosomes carried between 4 and 6 genes (**Figure 3**). This chromosome-specific distribution pattern may be associated with the genomic evolutionary history of *Gossypium barbadense*.

Gene duplication plays a major role in gene family expansion during evolution. Tandem duplication refers to the phenomenon where multiple copies of the same gene family are closely arranged and clustered together on a chromosome, whether segmental duplication refers to an evolutionary process in which a DNA segment containing one or more genes are duplicated, resulting in two or more copies that reside in non-adjacent regions of the genome. Analysis of the synteny among *GbDnaJ* family members identified only two tandem duplication events, involving the gene pairs *GbDnaJ45*/*GbDnaJ46* and *GbDnaJ26*/*GbDnaJ27*. Further investigation into segmental duplications within the *GbDnaJ* family revealed a complex evolutionary history. The analysis indicated numerous chromosomal segmental duplication events. The sequence similarity of the identified gene pairs varied widely (47.4%–97.5%), suggesting the occurrence of both ancient and recent duplication events. For instance, within the segmental duplications, *GbDnaJ51* (located on chromosome A01) and *GbDnaJ33* (on D01) displayed 97.5% sequence similarity, forming a highly conserved homologous gene pair indicative of a relatively recent duplication event. Such homologous pairs between the A and D subgenomes reflect the whole-genome duplication events that occurred during the allotetraploidization of *Gossypium barbadense*. In addition to inter chromosomal duplications, intrachromosomal duplications over long distances were also identified. For example, *GbDnaJ51* and *GbDnaJ52* are located on the same chromosome (A01) but are separated by approximately 1.22 Mb, with a sequence similarity of only 47.4%. This suggested that an ancient local duplication event have occurred.

**Figure 4:**
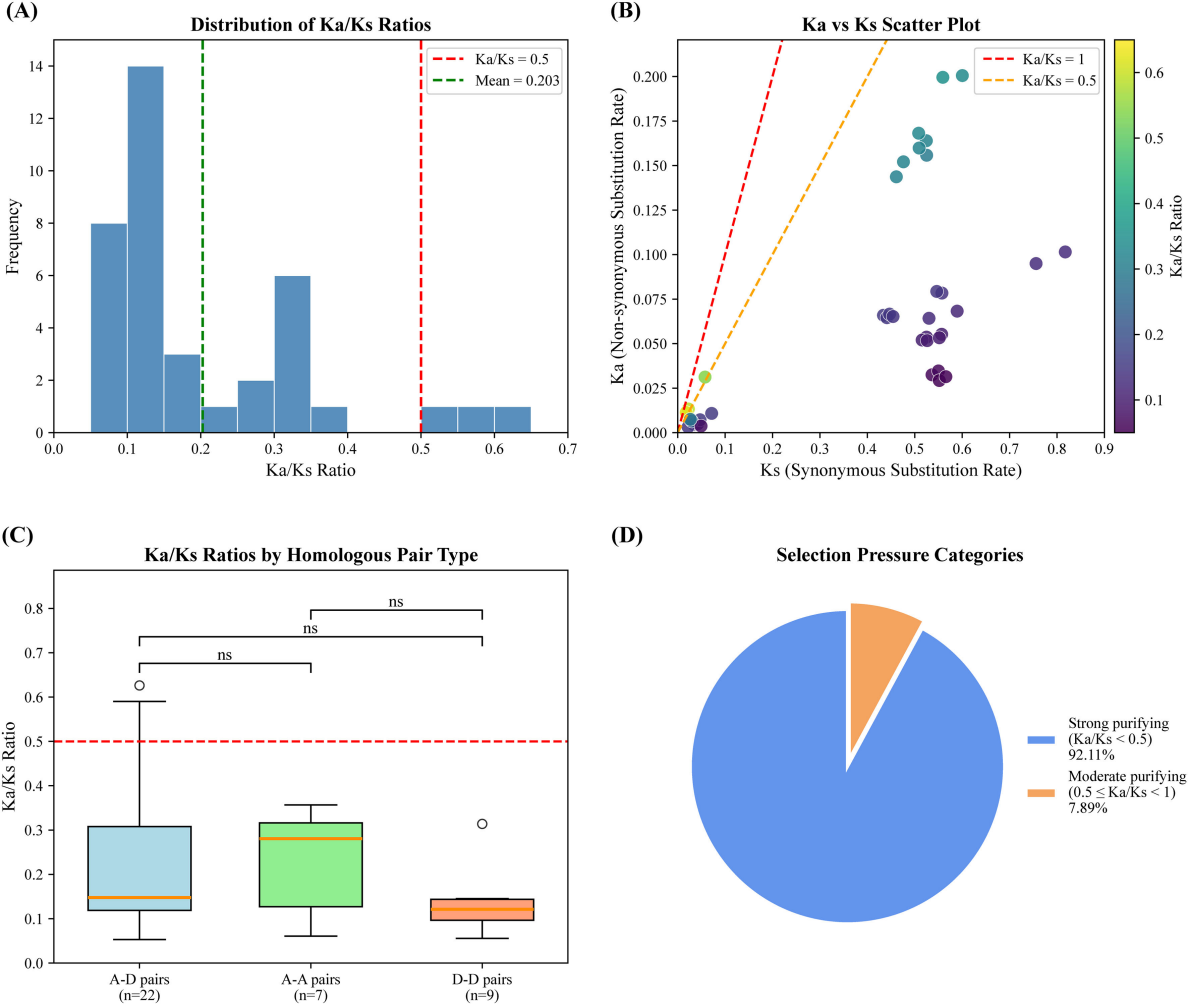
Selection pressure analysis of duplicated *GbDnaJ* genes in *Gossypium barbadense*. **(A)** Distribution of Ka/Ks ratios. The green dashed line represents the mean value, and the red dashed line indicates the neutral evolution threshold. **(B)** Scatter plot of Ka vs Ks. The red dashed line serves as the neutral evolution reference line, while the orange dashed line represents the strong purifying selection reference line. Low Ks values (0.02-0.1) indicate recent duplication events, whereas high Ks values (0.5-0.8) represent ancient duplication events. The color bar displays the corresponding Ka/Ks ratios, with purple indicating low Ka/Ks values (strong purifying selection) and yellow indicating higher Ka/Ks values. **(C)** Box plot of Ka/Ks ratios by homologous pair type. A-D pairs, homologous gene pairs between the A and D subgenomes; A-A pairs, homologous gene pairs within the A subgenome; D-D pairs, homologous gene pairs within the D subgenome. ‘ns’ indicates no statistically significant differences in Ka/Ks ratios were observed among the three groups (p > 0.05). Circles denote outliers; the red dashed line indicates the Ka/Ks=0.5 threshold. **(D)** Selection Pressure Categories.

To investigate the evolutionary patterns among *DnaJ* genes, we calculated the rate of synonymous substitution between duplicate gene pairs (**Figure 4**). The results indicate that *DnaJ* genes were generally under extensive purifying selection, as evidenced by the Ka/Ks ratios of all duplicate gene pairs being less than 1 (Ka/Ks range: 0.0530.626, mean: 0.203 ± 0.146) (**Table 2**) (**Figure 4A**). Further analysis demonstrated that 92.11% of gene pairs showed strong purifying selection (Ka/Ks < 0.5), while only 7.89% of gene pairs fell within the moderate purifying selection range (0.5 ≤ Ka/Ks < 1), and no gene pairs displayed evidence of positive selection (Ka/Ks ≥ 1) (**Figure 4D**). These findings indicate that the *DnaJ* gene family is highly conserved in Gossypium barbadense, with its functions being strictly constrained throughout long-term evolution. Further analysis of selection pressure patterns among different types of duplicated gene pairs revealed subgenome-specific differences (**Figure 4C**). A-D homologous gene pairs (n=22, representing homologous copies generated by whole-genome duplication) displayed a mean Ka/Ks of 0.224, indicating moderate purifying selection. A-A intra-chromosomal homologous gene pairs (n=7) showed comparable values to A-D pairs, with a mean Ka/Ks of 0.226. Notably, D-D intra-chromosomal duplicated pairs (n=9) exhibited significantly lower mean Ka/Ks values (0.133), suggesting that duplicated genes in the D subgenome are subject to stronger functional constraints. Scatter plot analysis of Ka versus Ks revealed that the synonymous substitution rate Ks varied considerably (0.0180.818), reflecting duplication events occurring at different evolutionary times; however, the non-synonymous substitution rate Ka consistently remained at low levels (0.0030.201), further confirming the sustained functional constraints throughout evolutionary history (**Figure 4B**). Collectively, these findings demonstrate that although tandem duplication and segmental duplication events have contributed to the numerical expansion of the *GbDnaJ* gene family, strong purifying selection has constrained sequence divergence and· functional innovation among these duplicated genes.

**Table 2:** Nonsynonymous (Ka), synonymous (Ks)and ka/ks ratio between duplicate gene pairs of *Gossypium barbadense*.

Gene_id	Gene_id	Ka	Ks	Ka/Ks
DnaJ49	DnaJ30	0.013328748	0.022589943	0.590030161
DnaJ50	DnaJ32	0.004551215	0.034324388	0.132594205
DnaJ51	DnaJ60	0.053686001	0.524843792	0.102289484
DnaJ51	DnaJ58	0.055279617	0.556794513	0.0992819
DnaJ52	DnaJ80	0.052063057	0.515611082	0.100973503
DnaJ3	DnaJ73	0.031246227	0.057720781	0.541334107
DnaJ99	DnaJ74	0.004912011	0.04196899	0.117039056
DnaJ56	DnaJ42	0.152125728	0.476228895	0.319438256
DnaJ56	DnaJ23	0.199586126	0.559282133	0.356861259
DnaJ56	DnaJ78	0.143709211	0.461209825	0.311591824
DnaJ56	DnaJ95	0.200560342	0.600434269	0.334025475
DnaJ35	DnaJ13	0.078409213	0.5573131	0.140691495
DnaJ39	DnaJ69	0.003190928	0.022073942	0.144556349
DnaJ107	DnaJ59	0.032522578	0.53646623	0.060623719
DnaJ107	DnaJ57	0.034688263	0.550059572	0.063062739
DnaJ62	DnaJ45	0.065947842	0.434015265	0.151948208
DnaJ62	DnaJ103	0.011342371	0.018114088	0.626162971
DnaJ62	DnaJ26	0.064605136	0.441281955	0.146403303
DnaJ59	DnaJ109	0.029310315	0.551790017	0.053118603
DnaJ60	DnaJ33	0.051724552	0.526202512	0.098297805
DnaJ60	DnaJ58	0.007267279	0.047210129	0.153934735
DnaJ45	DnaJ103	0.066601645	0.446844033	0.149048974
DnaJ47	DnaJ105	0.101511223	0.817504788	0.124172023
DnaJ47	DnaJ28	0.010842746	0.071665733	0.1512961
DnaJ42	DnaJ23	0.163972771	0.52449003	0.312632771
DnaJ42	DnaJ95	0.168193487	0.508363359	0.330852892
DnaJ20	DnaJ92	0.006524735	0.029845405	0.21861775
DnaJ23	DnaJ78	0.155820201	0.52515008	0.296715563
DnaJ33	DnaJ58	0.053324886	0.551887505	0.096622746
DnaJ34	DnaJ80	0.064258446	0.530101292	0.121219183
DnaJ72	DnaJ106	0.068269721	0.589489642	0.11581157
DnaJ66	DnaJ13	0.079346653	0.546469404	0.145198711
DnaJ109	DnaJ57	0.031464896	0.565642081	0.055626865
DnaJ103	DnaJ26	0.065258533	0.454233212	0.143667462
DnaJ105	DnaJ28	0.095000574	0.755955498	0.125669532
DnaJ78	DnaJ95	0.159865174	0.509342062	0.313866035
DnaJ26	DnaJ27	0.003752353	0.049308268	0.076099875
DnaJ45	DnaJ46	0.007503971	0.026738814	0.280639631

**Figure 5:**
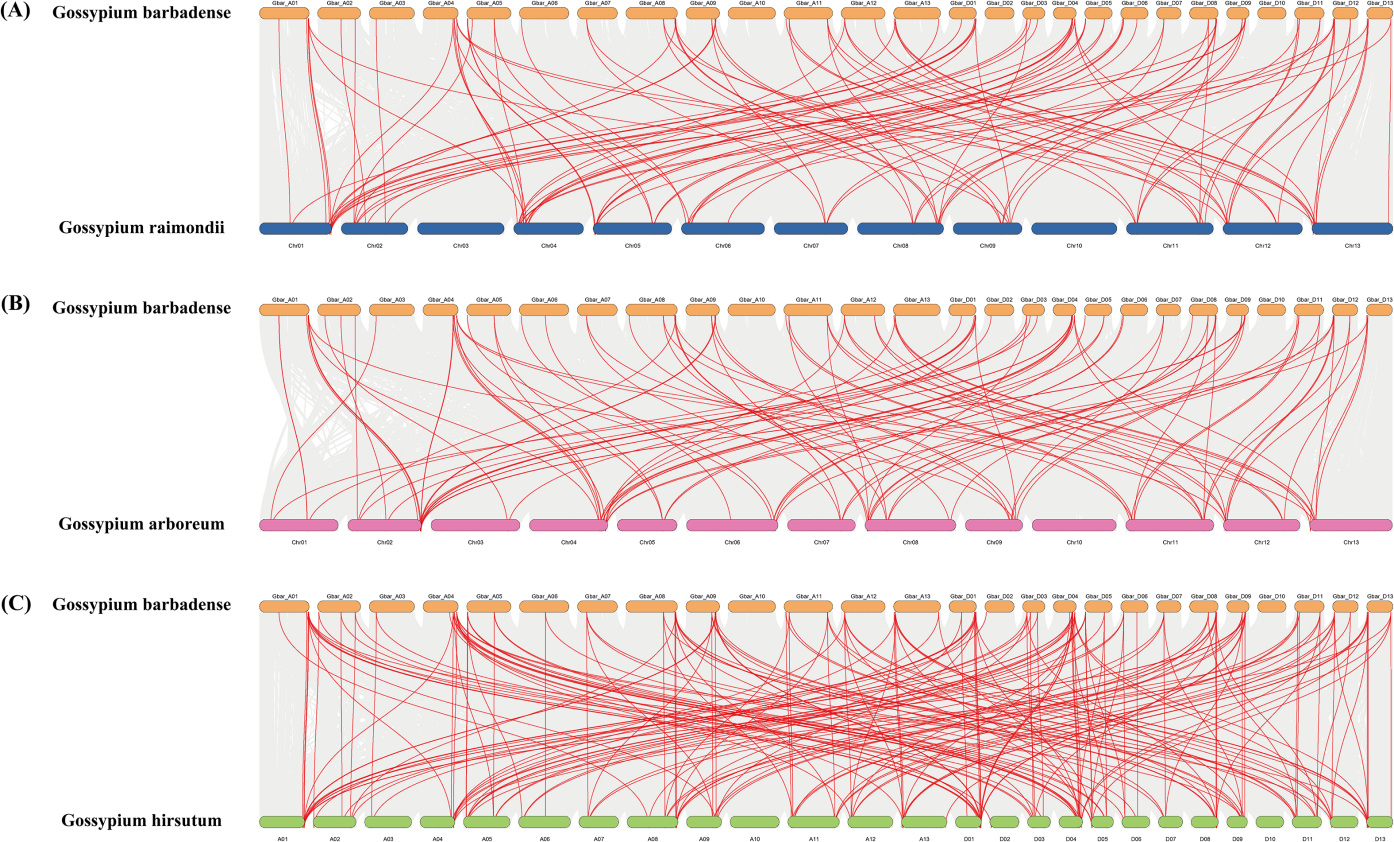
Collinearity analysis of *DnaJ* genes between *Gossypium barbadense, Gossypium hirsutum, Gossypium arboreum* and *Gossypium raimondii*. **(A)***Gossypium barbadense* and *Gossypium raimondii*, **(B)***Gossypium barbadense* and *Gossypium arboreum*, **(C)***Gossypium barbadense* and *Gossypium hirsutum*. The gray lines in the background represent genomic collinearity between *Gossypium barbadense* and the other three cotton species, while the red lines highlight syntenic *DnaJ* gene pairs.

To investigate the evolutionary history of the *DnaJ* gene family in *Gossypium barbadense*, a comparative genomic analysis was conducted using the coding sequences of its 109 *GbDnaJ* genes and the whole genomes of other representative cotton species, including *Gossypium hirsutum*, *Gossypium arboreum* (A-genome donor), and *Gossypium raimondii* (D-genome donor) (**Figure 5**). The analysis revealed 108 syntenic gene pairs between *Gossypium barbadense* and the similarly allotetraploid *Gossypium hirsutum*, a number nearly equivalent to the total count of *GbDnaJ* genes. This high degree of synteny indicates that the *DnaJ* gene family has been extensively retained and is highly conserved in both modern cultivated allotetraploid cotton species, with largely preserved genomic structures and membership since their divergence. In contrast, only 53 and 59 syntenic gene pairs were identified between *Gossypium barbadense* and its putative diploid progenitors, *Gossypium arboreum* (A-genome) and *Gossypium raimondii* (D-genome), respectively.

**Figure 6:**
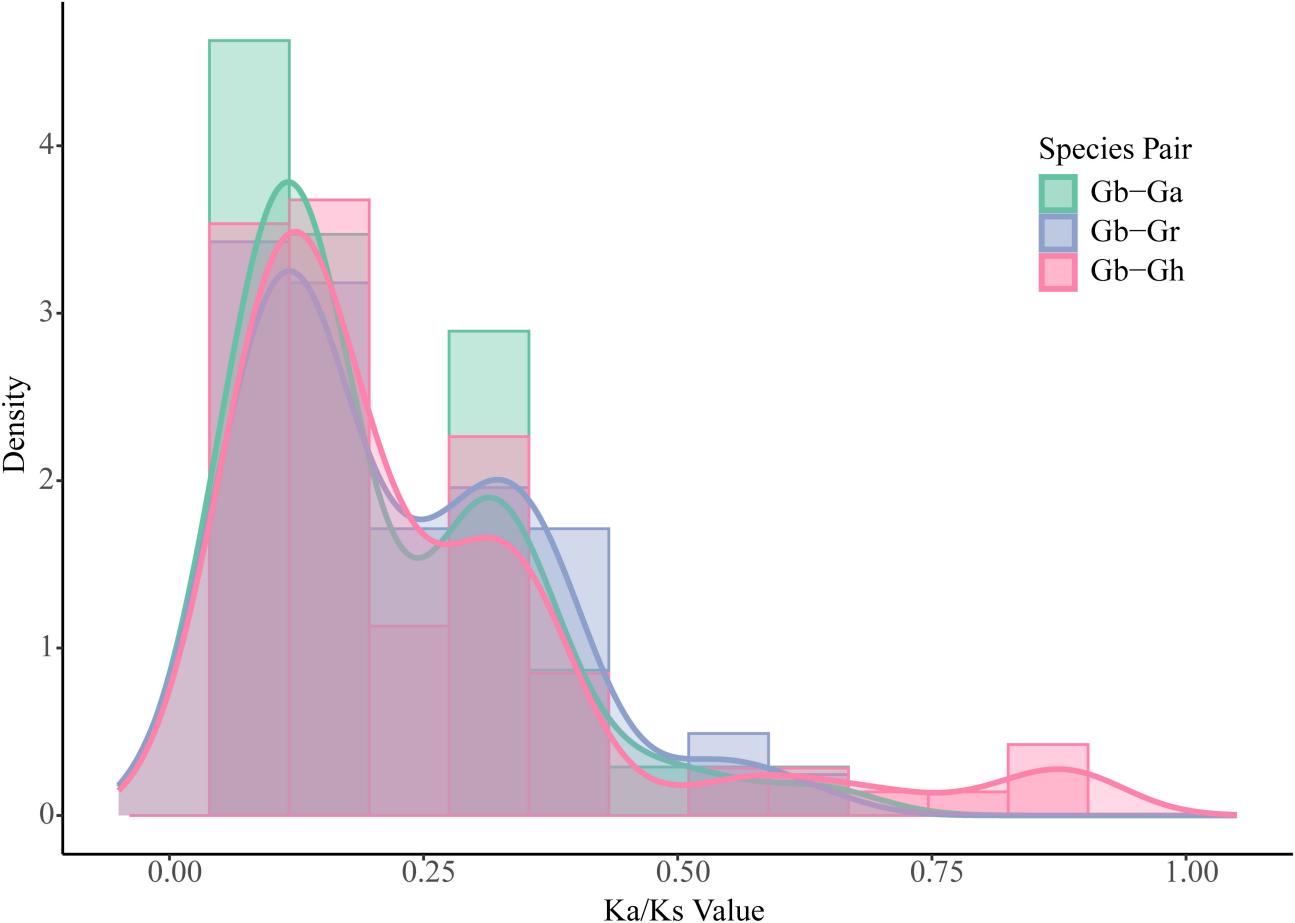
Selection pressure analysis of *DnaJ* genes between *Gossypium barbadense* and related Gossypium species. *Gossypium barbadense*(Gb), *Gossypium arboreum*(Ga), *Gossypium raimondii*(Gr), and *Gossypium hirsutum*(Gh). The histogram with kernel density estimation (KDE) curves shows the Ka/Ks value distribution for three species pairs: Gb-Ga(green), Gb-Gr(blue) and Gb-Gh(pink). The x-axis represents Ka/Ks values, and the y-axis represents density. Most gene pairs (>98.9%) exhibit Ka/Ks < 1, indicating predominant purifying selection across all species comparisons.

To investigate evolutionary patterns among *DnaJ* genes, we calculated the rate of synonymous substitutions between the *DnaJ* family among *Gossypium hirsutum*(Gh), *Gossypium arboreum*(Ga), *Gossypium raimondii*(Gr), and *Gossypium barbadense*(Gb) (**Figure 6**). The results showed that 98.9% of homologous gene pairs between Gb and Gh, Ga, and Gr exhibited Ka/Ks values less than 1 (mean = 0.2494, median = 0.1547), demonstrating that *DnaJ* genes have been generally under purifying selection during evolution, suggesting that *DnaJ* genes may have played important roles in the evolutionary process of species. Ka/Ks analysis revealed similar selection pressure patterns among the three species pairs, with mean Ka/Ks values of 0.2054 and 0.2200 for Gb-Ga and Gb-Gr, respectively, showing no statistically significant differences among groups (Mann-Whitney U test, *p* > 0.05), indicating that both A and D subgenomes experienced comparable evolutionary constraints and selection pressures following polyploidization.

### 3.4 Conserved protein motifs and gene structure analysis of the *GbDnaJ* gene family

**Figure 7:**
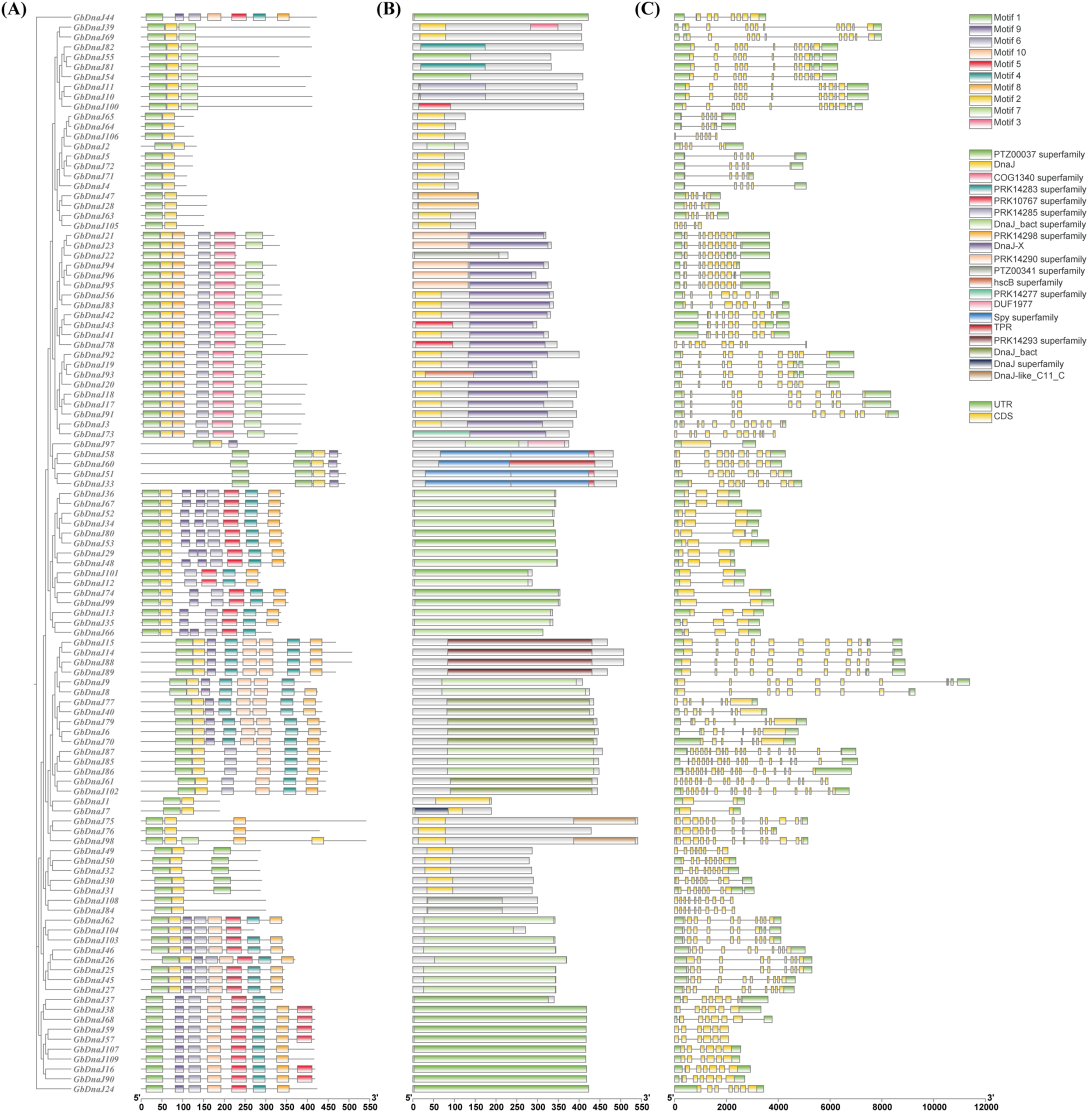
Phylogenetic tree, conserved motifs, and gene structure of *GbDnaJ* members. **(A)** Distribution of conserved protein motifs identified by MEME Suite (motifs 1-10). Each motif is represented by a colored box. **(B)** Conserved GbDnaJ domains as predicted by NCBI CDD. **(C)** Exon-intron structures of *GbDnaJ* genes. Boxes in different colors represent distinct motifs or exons. Black lines indicate non-conserved sequences and introns.

To infer the evolutionary relationships among *GbDnaJ*, the phylogenetic tree of 109 GbDnaJ proteins was constructed. Based on conserved domains, these members were classified into five subgroups (**Figure 7B**). A total of 10 distinct conserved motifs (named motifs 1-10) were identified (**Figure 7A**). Among these, motif 1 was present in all members, while the vast majority contained motif 2 and motif 6. Furthermore, 16 GbDnaJ proteins (GbDnaJ1, GbDnaJ2, GbDnaJ4, GbDnaJ5, GbDnaJ7, GbDnaJ28, GbDnaJ47, GbDnaJ63, GbDnaJ64, GbDnaJ65, GbDnaJ71, GbDnaJ72, GbDnaJ84, GbDnaJ105, GbDnaJ106, GbDnaJ108) contained only two motifs. Subsequently, the distribution of introns and exons in *GbDnaJ* genes was analyzed to explore their structural characteristics (**Figure 7C**). The results revealed substantial variation in the number of exons (ranging from 4 to 21) and introns (ranging from 0 to 20) among members of the gene family. Notably, most *GbDnaJ* genes within the same subgroup generally exhibited similar exon numbers and gene structures.

### 3.5 Cis-acting regulatory elements in *GbDnaJ* promoters

**Figure 8:**
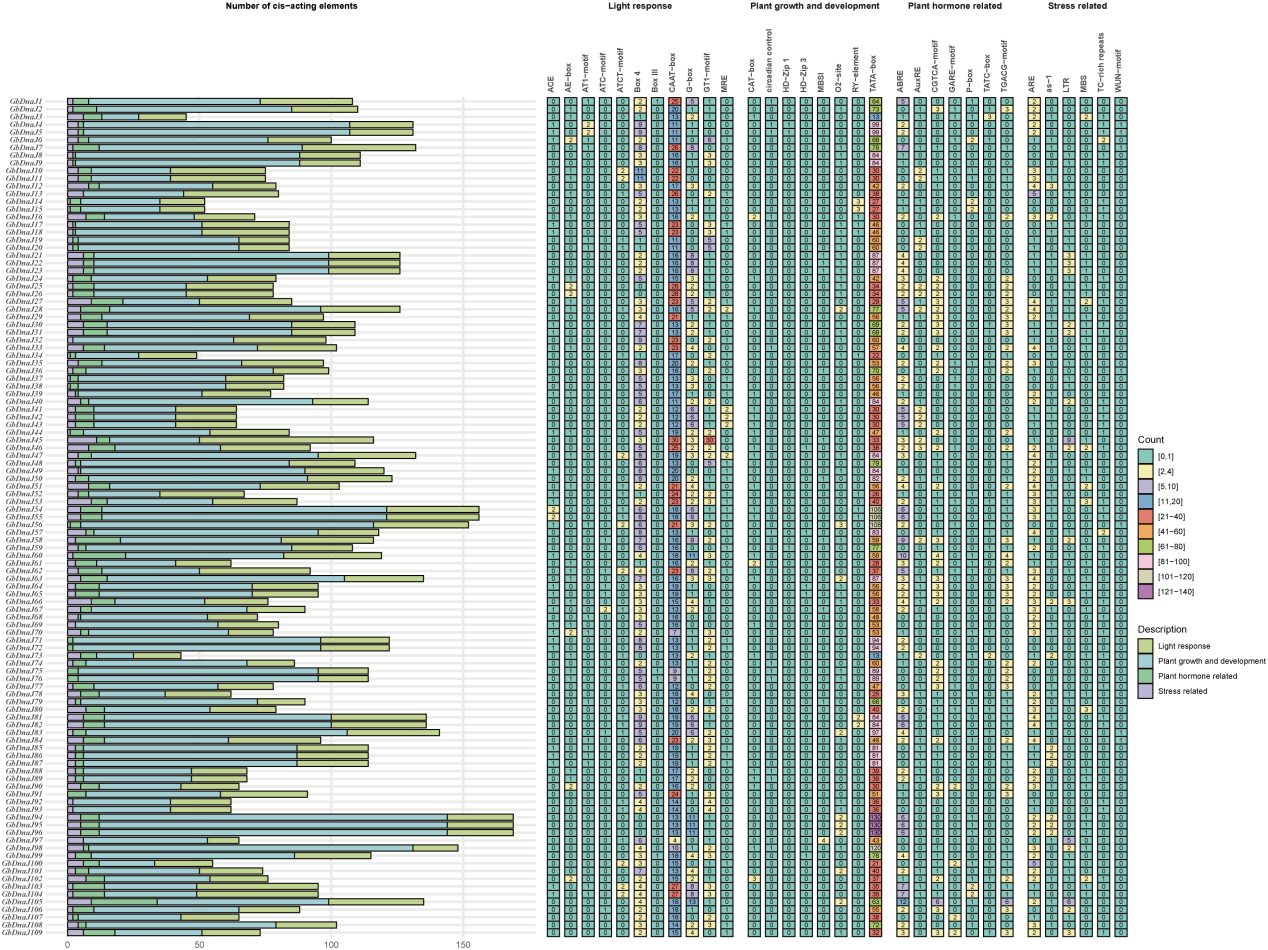
Cis-acting regulatory element number analysis in the *GbDnaJ* gene family. Right panel: The histogram with different colors shows the total number of cis-acting regulatory elements for each category. Left panel: The varying color intensities and numbers in the grid represent the number of different promoter elements within the *GbDnaJ* genes.

Cis-acting regulatory elements in gene promoters serve as binding sites for transcription factors, enabling the integration of external stimuli through the regulation of gene expression. To analyze the upstream regulatory mechanisms of the *GbDnaJ* gene family, conserved cis-acting regulatory elements were identified within the 2000 bp promoter regions upstream of *GbDnaJ* genes using PlantCARE. A total of 32 conserved cis-acting elements were identified and primarily categorized into four functional classes: light responsiveness, plant growth and development, phytohormone response, and stress-related elements (**Figure 8**). Eight elements were associated with plant growth and development: CAT-box, circadian control, HD-Zip 1, HD-Zip 3, MBSI, O2-site, RY-element, and TATA-box. Within the light responsiveness category, several elements were identified, including ACE, AE-box, AT1-motif, ATC-motif, ATCT-motif, Box 4, Box I, CAAT-box, G-box, GT1-motif, and MRE. Notably, the CAAT-box was present in all *GbDnaJ* genes, with GbDnaJ26, GbDnaJ27, and GbDnaJ45 containing the highest numbers of this element (28 and 30, respectively). These results suggest that the expression of *GbDnaJ* genes may be regulated by light. Eight types of cis-acting elements were related to phytohormone responses: ABRE (254 occurrences), AuxRE (58), CGTCA-motif (117), GARE-motif (21), P-box (31), TATC-box (20), and TGACG-motif (117). Among the phytohormone-responsive elements, the ABRE (abscisic acid-responsive element) was the most abundant (254 occurrences), followed by the two MeJA-responsive elements (CGTCA-motif and TGACG-motif, 117 occurrences each), and the gibberellin-responsive P-box element (31 occurrences). Furthermore, it was observed that 32.5%, 19.3%, and 17.8% of the *GbDnaJ* family members contained the ARE (anaerobic induction element), As-1 (activation sequence-1), and LTR (low-temperature responsiveness) elements, respectively. These findings indicate that *GbDnaJ* genes may play important roles in regulating light responses, plant growth and development, phytohormone signaling, and stress responses.

### 3.6 Interacting protein prediction of GbDnaJs and GbaHSP70s

**Figure 9:**
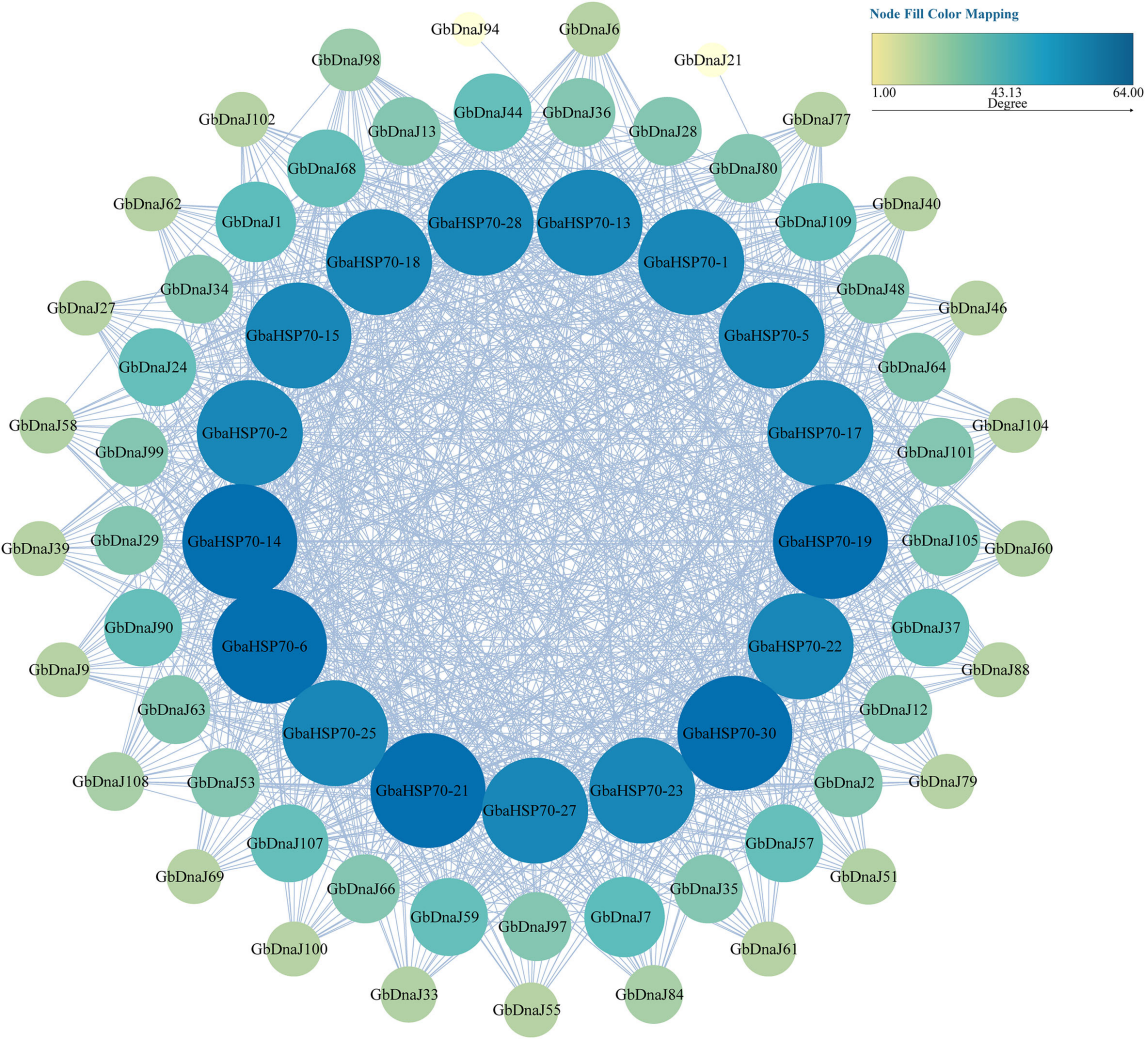
Protein interaction network between GbDnaJs and GbaHSP70s. Nodes represent proteins, and both node size and color are positively correlated with Degree. Edges represent interaction relationships between proteins. Node colors adopt a continuous gradient color scale, where light yellow indicates Degree=1 and dark blue indicates Degree=64.

The potential interactions between GbDnaJs and GbaHSP70s proteins were analyzed using STRING (https://cn.string-db.org/). The results showed that 54 GbDnaJs interacted with 17 GbaHSP70s proteins (**Figure 9**). The confidence level of DnaJ-HSP70 interactions was significantly higher than that of intra-family interactions, which is highly consistent with the functional role of DnaJ as a molecular chaperone co-factor for HSP70. HSP70 proteins exhibited significant centrality in this network, with all 17 HSP70 proteins being hub nodes (Degre>50). Among these, GbaHSP70-6, GbaHSP70-14, GbaHSP70-19, GbaHSP70-21, and GbaHSP70–30 interacted with more than 90% of the proteins in the network. Specifically, GbaHSP70–21 exhibited strong interactions with 7 GbDnaJs, serving as a key responsive node in the GbaHSP70 family. GbDnaJ proteins showed high clustering coefficients, indicating that DnaJ proteins formed relatively tight functional modules. Specifically, GbDnaJ1 (Degree=37) interacted with 12 HSP70 members, acting as a core hub in the interaction network and playing a key role in the high-temperature adaptation of *Gossypium barbadense*. GbDnaJ105 exhibited the highest betweenness centrality, serving as a critical bridge node for information flow in the network.

### 3.7 Expression profiles of *GbDnaJs* under heat stress

**Figure 10:**
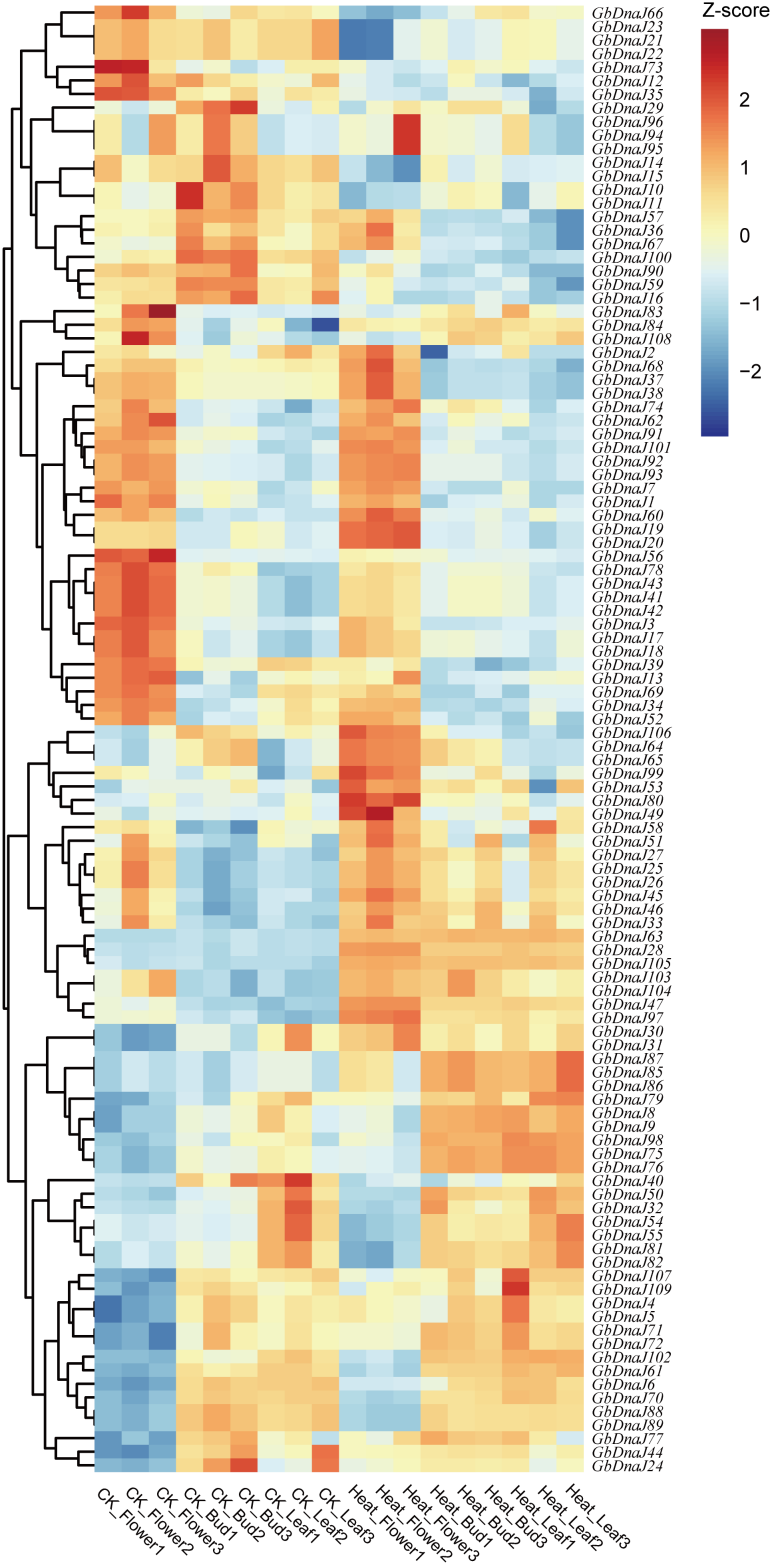
Gene expression profiles of *GbDnaJ* genes under heat stress. Blue and orange scales indicate low and high expression levels, respectively.

Based on RNA-seq data, TPM values of *Gossypium barbadense* tissues (flowers, buds, leaves) under control and 40°C heat stress treatment revealed expression profiles and potential functions of *GbDnaJ* members (**Supplementary Table 2**). A total of 67 genes were upregulated and 42 downregulated (**Figure 10**), including 16 with significant changes in all tissue. In flowers, 10 genes were significantly upregulated, among which *GbDnaJ1*, *GbDnaJ36*, *GbDnaJ57*, *GbDnaJ67*, and *GbDnaJ101* were specifically induced under heat stress. Conversely, *GbDnaJ3*, *GbDnaJ21*, *GbDnaJ56*, and *GbDnaJ59* were significantly downregulated in flowers, with *GbDnaJ3* and *GbDnaJ56* being specifically suppressed. In buds, 7 genes upregulated (*GbDnaJ3*, *GbDnaJ28*, *GbDnaJ47*, *GbDnaJ60*, *GbDnaJ63*, *GbDnaJ81*, *GbDnaJ105*) included stress-specific *GbDnaJ3*, whereas *GbDnaJ1*, *GbDnaJ21*, *GbDnaJ36*, *GbDnaJ57*, *GbDnaJ59*, *GbDnaJ67* downregulated. In leaves, *GbDnaJ28*, *GbDnaJ47*, *GbDnaJ63*, and *GbDnaJ105* were significantly upregulated, while 7 genes were significantly downregulated. These findings indicate that *GbDnaJ* genes play diverse roles in the response of *Gossypium barbadense* to heat stress. Notably, *GbDnaJ1*, *GbDnaJ3*, *GbDnaJ36*, *GbDnaJ56*, *GbDnaJ57*, *GbDnaJ67*, and *GbDnaJ101* were induced across flowers, buds, and leaves, but exhibited distinct expression trends. For instance, *GbDnaJ1*, *GbDnaJ36*, *GbDnaJ57*, and *GbDnaJ67* were upregulated in flowers but downregulated in both buds and leaves after heat stress, suggesting differential regulatory mechanisms for maintaining cellular homeostasis across tissues. *GbDnaJ56* was sharply downregulated in flowers yet upregulated in buds and leaves under heat stress; however, its expression level in flowers remained substantially higher than in buds and leaves both before and after stress, indicating it may be a tissue-specific gene that negatively regulates the heat stress response in *Gossypium barbadense*. Collectively, these findings demonstrate that *GbDnaJ* genes play diverse roles in the response of *Gossypium barbadense* to heat stress, and their tissue-specific expression patterns contribute to the functional complexity of this gene family.

### 3.8 Quantitative expression analysis of *GbDnaJs* under heat stress

**Figure 11:**
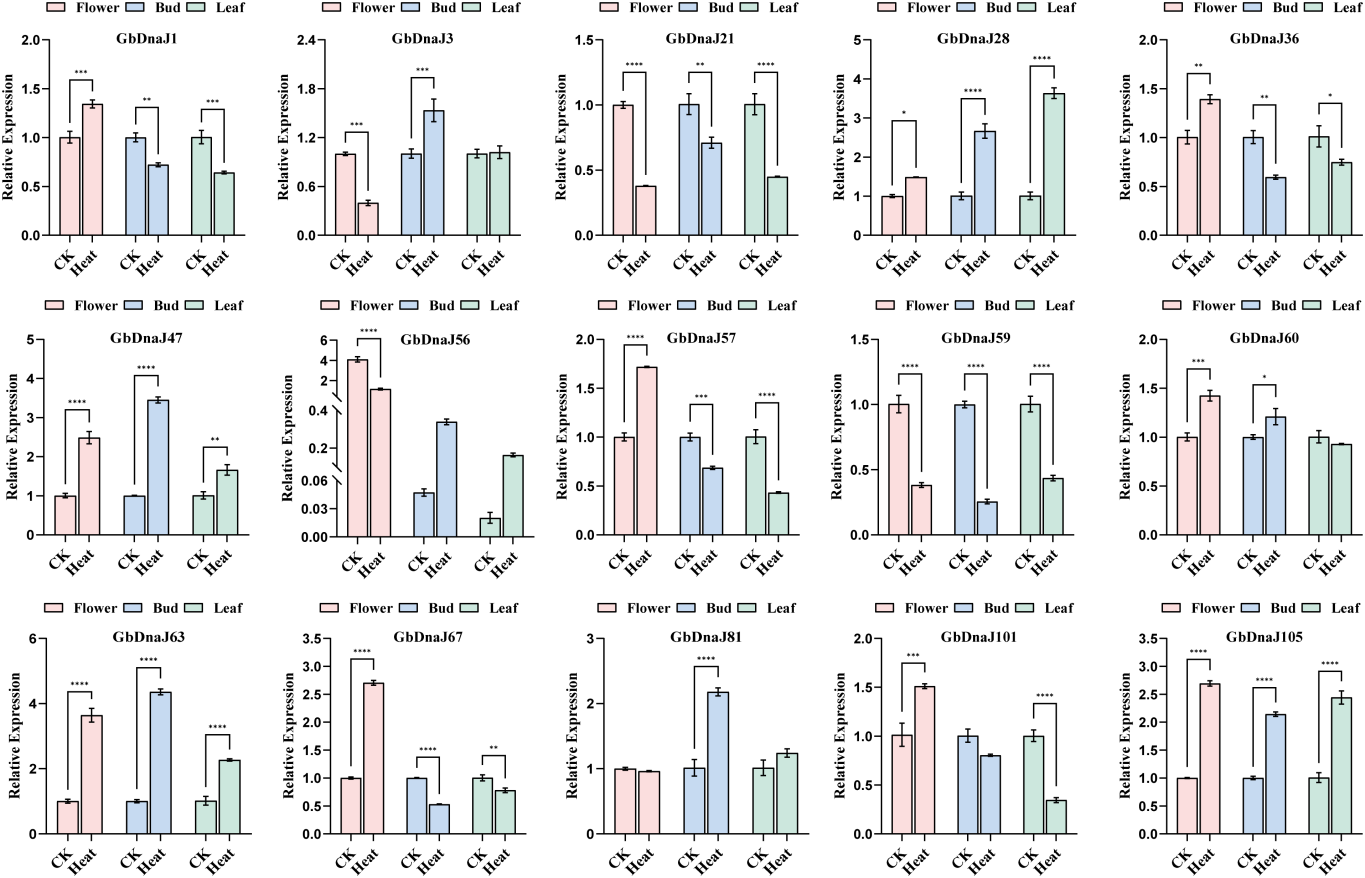
qRT-PCR analysis of 15 selected *GbDnaJ* genes. Expression profiles(qRT-PCR) of *GbDnaJ* genes under control (CK, normal temperature) and 40°C heat stress conditions. Comparative relative expression levels across different tissues are shown. The asterisks indicate statistically significant differences (*p < 0.05; **p < 0.01; ***p < 0.001; ****p < 0.0001) based on t-test. Data without significant differences are not labeled in the figures (P > 0.05).

To further validate the response of *GbDnaJ* genes to heat stress, we analyzed their expression by qRT-PCR (**Figure 11**). Fifteen highly responsive members (*GbDnaJ1, GbDnaJ3, GbDnaJ21, GbDnaJ28, GbDnaJ36, GbDnaJ47, GbDnaJ56, GbDnaJ57, GbDnaJ59, GbDnaJ60, GbDnaJ63, GbDnaJ67, GbDnaJ81, GbDnaJ101*, and *GbDnaJ105*) were selected for this analysis(|log2FoldChange| > 2 && pvalue < 0.01).

Among the upregulated genes in flowers, heat stress induced the highest expression of GbDnaJ63 (>3-fold), followed by GbDnaJ67 and GbDnaJ105 (>2-fold); the remaining upregulated genes showed 1–2-fold increases. In buds, seven genes (GbDnaJ3, GbDnaJ28, GbDnaJ47, GbDnaJ60, GbDnaJ63, GbDnaJ81, GbDnaJ105) were upregulated, with GbDnaJ63 exhibiting the strongest induction (>4-fold), followed by GbDnaJ47 (>3-fold). Conversely, *GbDnaJ1, GbDnaJ21, GbDnaJ36, GbDnaJ57, GbDnaJ59*, and *GbDnaJ67* were significantly downregulated in buds, with *GbDnaJ59* showing the most pronounced decrease. In leaves, *GbDnaJ28, GbDnaJ47, GbDnaJ63*, and *GbDnaJ105* were significantly upregulated, with *GbDnaJ28* showing the highest induction (>3-fold). Meanwhile, *GbDnaJ1, GbDnaJ21, GbDnaJ36, GbDnaJ57, GbDnaJ59*, and *GbDnaJ101* were significantly downregulated, with *GbDnaJ59* and *GbDnaJ101* exhibiting the most substantial reductions. Furthermore, distinct expression patterns were observed across tissues. *GbDnaJ21, GbDnaJ28, GbDnaJ47, GbDnaJ59, GbDnaJ63*, and *GbDnaJ105* displayed similar expression trends in flowers, buds, and leaves. *GbDnaJ1, GbDnaJ36, GbDnaJ56, GbDnaJ57, GbDnaJ67, GbDnaJ81*, and *GbDnaJ101* showed comparable patterns in leaves and buds, while *GbDnaJ60* exhibited similar expression in flowers and buds. Particularly noteworthy was *GbDnaJ56*, which was upregulated in buds and leaves but downregulated in flowers, with its expression level in flowers being substantially higher (>10-fold) than in buds and leaves both before and after stress treatment. These results align with transcriptomic analysis, supporting the hypothesis that *GbDnaJ* genes play tissue-specific roles in the heat stress response. This enhances understanding of adaptation mechanisms in *Gossypium barbadense* under heat stress.

### 3.9 Analysis of physiological, biochemical and photosynthetic characteristics under heat stress

**Figure 12:**
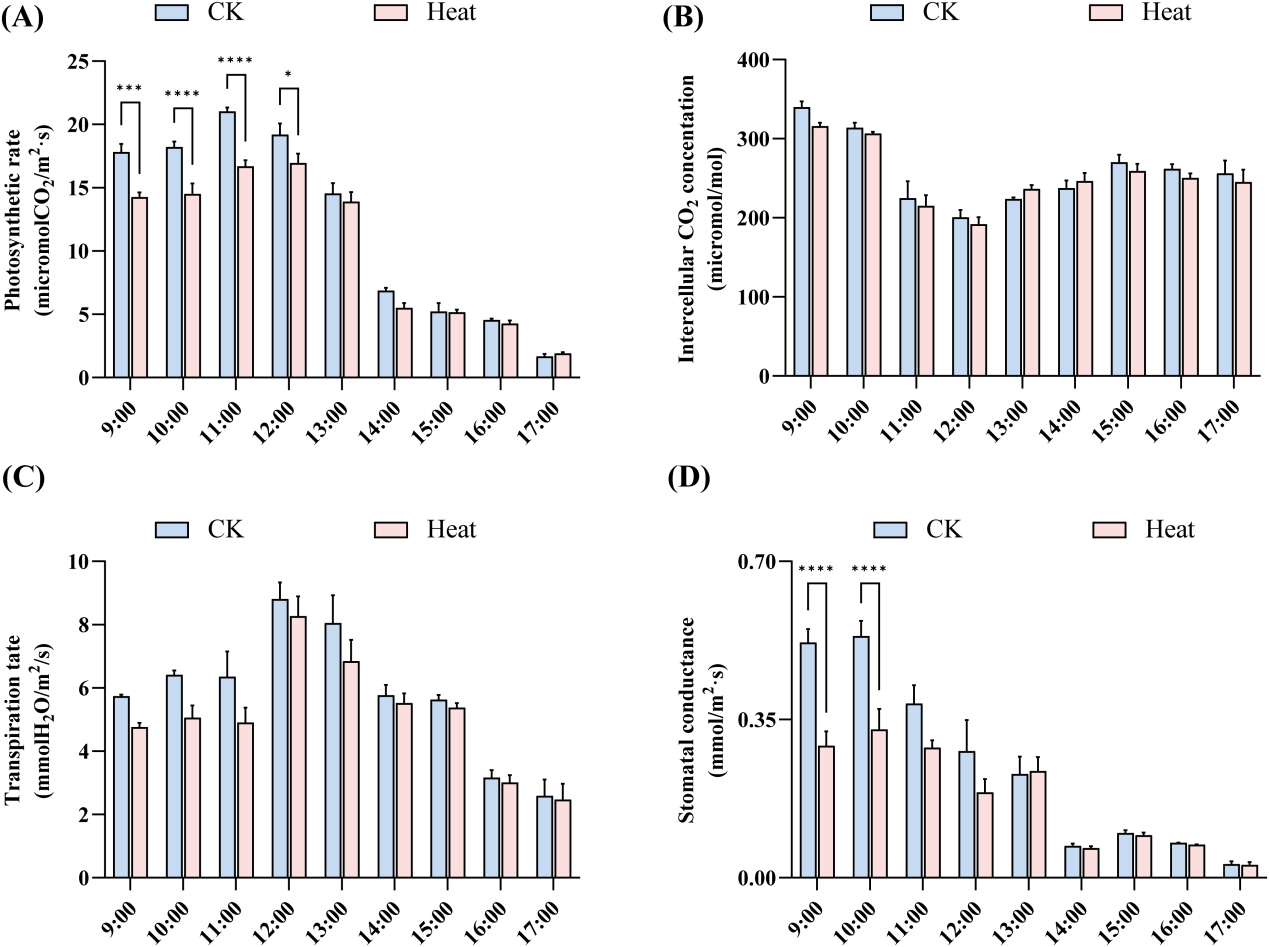
Vairation in photosynthetic parameters of Gossypium barbadense under heat stress. **(A)** Photosynthetic rate (Pn), **(B)** Intercellular CO₂ concentration (Ci), **(C)** Transpiration rate (Tr), and **(D)** Stomatal conductance (Gs) under control and heat stress conditions. The asterisks indicate statistically significant differences (*p < 0.05; ***p < 0.001; ****p < 0.0001) based on t-test. Data without significant differences are not labeled in the figures (P > 0.05).

Under normal temperature conditions, the photosynthetic rate increased gradually during the morning, peaked at 11:00 (21.03 μmol m^−2^ s^−1^), then progressively declined with a significant reduction by 14:00 and minimum values at 17:00. Heat stress consistently suppressed photosynthetic rates, particularly during the morning (9:00-12:00), though this suppression attenuated during afternoon hours (**Figure 12A**). Transpiration rates followed patterns similar to photosynthesis (**Figure 12C**). Under control conditions, stomatal conductance remained higher in the morning (0.52 mol m^−2^ s^−1^ at 9:00) before gradually decreasing after 12:00. Heat-stressed plants maintained lower stomatal conductance throughout the day, with more pronounced suppression during morning hours (0.29 mol m^−2^ s^−1^ at 9:00, 44% reduction) and diminished differences in the afternoon (**Figure 12D**). Both groups showed initial decreases followed by increases in intercellular CO_2_ concentration, with a rebound observed at 15:00 (**Figure 12B**).

**Figure 13:**
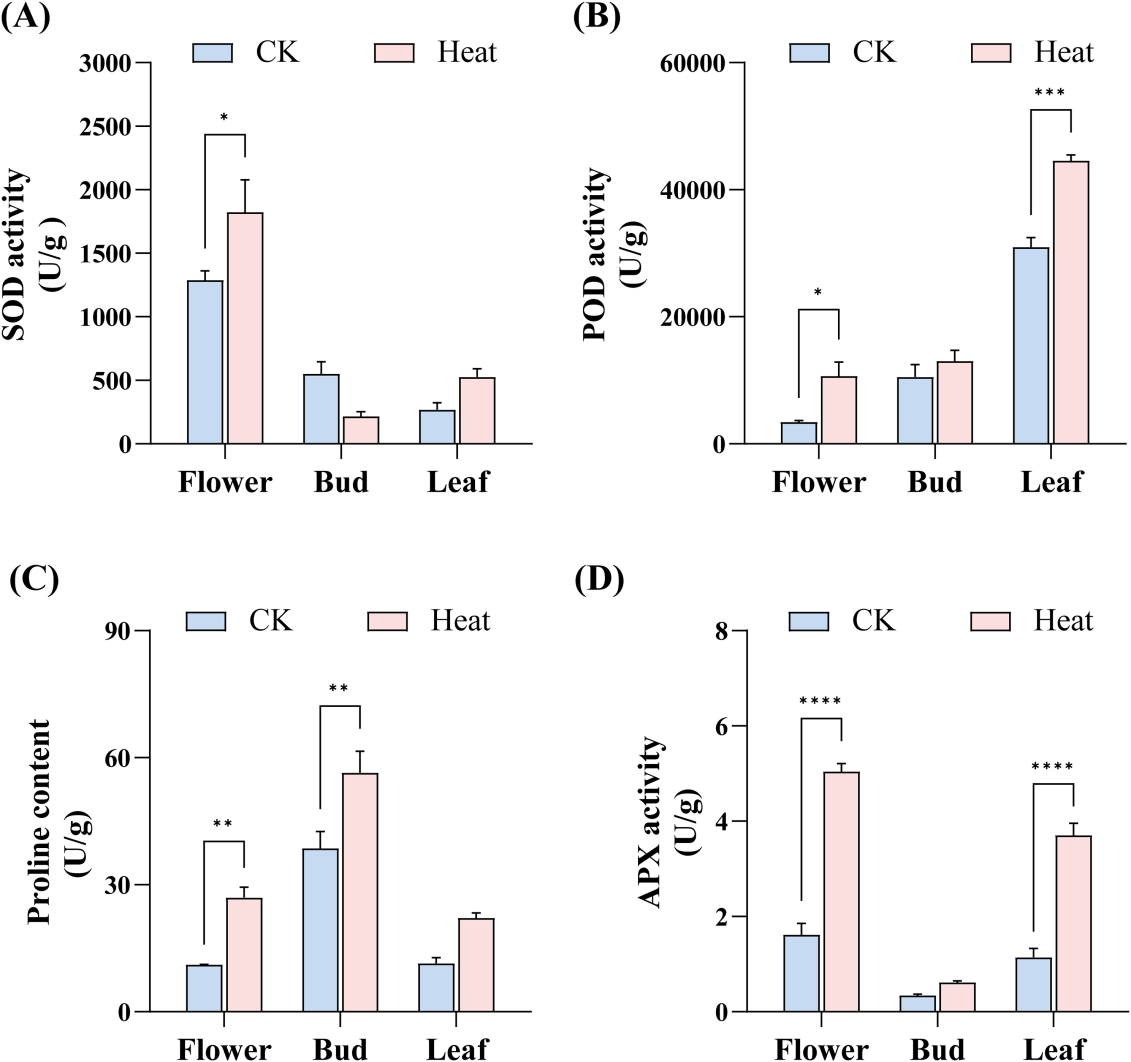
Vairation in physiological and biochemical parameters of *Gossypium barbadense* under heat stress. **(A)** SOD activity, **(B)** POD activity, **(C)** Proline content, and **(D)** APX activity in flowers, buds, and leaves. The asterisks indicate statistically significant differences (*p < 0.05; **p < 0.01; ***p < 0.001; ****p < 0.0001) based on t-test. Data without significant differences are not labeled in the figures.

The contents of SOD (**Figure 13A**), POD (**Figure 13B**), Pro (**Figure 13C**), and APX (**Figure 13D**) in various tissues basically increased after heat stress. APX increased significantly in flowers and leaves (**Figure 13D**), while POD showed marked elevation in leaves (**Figure 13B**). The abundance of light-responsive elements in the *DnaJ* gene family suggests potential disruption of light-responsive pathways under high light intensity, potentially contributing to stomatal closure and photosynthetic inhibition. Concurrently, stress-responsive elements enable thermal induction of *DnaJ* expression, potentially mitigating heat damage through protection of Photosystem II and electron transport chains, reduction of reactive oxygen species accumulation, maintenance of cellular homeostasis, and facilitation of protein refolding.

## 4 Discussion

### 4.1 Identification of the *DnaJ* gene family

DnaJ protein, also known as HSP40, is a class of heat shock proteins widely present in plants and responsive to both biotic and abiotic stresses. DnaJ was initially identified for its ability to stimulate the ATPase activity of the bacterial HSP70 homolog DnaK (Yochem and Uchida, 1978). Numerous DnaJ homologs have since been discovered in both prokaryotes and eukaryotes; for instance, 66 DnaJ homologs have been identified in *Escherichia coli* and 22 in *Saccharomyces cerevisiae* (Walsh et al., 2004). Many studies have reported the important roles of the *DnaJ* gene family in plant growth, development, and stress responses (Petitjean et al., 2012; Chen et al., 2021b; Hu et al., 2022; Zhang et al., 2023). Previous researchs have shown that the expression levels of *DnaJ* genes in plants can change under various stress conditions (Fan et al., 2017; Singh et al., 2024). Recent studies have further demonstrated the significance of DnaJ proteins in plant stress responses. For example, overexpression of *Castanea mollissima CmDnaJ27* in *Nicotiana tabacum* significantly reduced its tolerance to both cold and heat stresses (Yu et al., 2025). In this work, we identified 109 *GbDnaJ* genes in *Gossypium barbadense* through bioinformatic methods. These genes are unevenly distributed across all 26 chromosomes and exhibit diverse physicochemical properties, with significant variations in size, gene structure, and expression patterns. The subcellular localization of a protein is closely related to its function; therefore, understanding protein localization is crucial for functional studies. In plants, DnaJ proteins are localized to different compartments such as the cytoplasm, nucleus, chloroplasts, mitochondria, and endoplasmic reticulum (Silver and Way, 1993; Cyr et al., 1994; Schlicher and Soll, 1997). For instance, the plasma membrane-localized OsDnaJ15 in rice interacts with OsBAG4 to promote the DNA-binding activity of OsMYB106, thereby upregulating *OsHKT1* expression and enhancing salt tolerance (Liu et al., 2023) Regarding chloroplast-localized proteins, tomato SlCDJ2 protects the CO_2_ assimilation capacity under heat stress by maintaining low proteolytic activity, thereby preventing the accelerated degradation of Rubisco (ribulose-1,5-bisphosphate carboxylase/oxygenase) (Wang et al., 2015). In *Arabidopsis thaliana*, AtJ11 interacts with the maize protein Bsd2, and AtJ20 protects the photosynthetic apparatus under high-temperature stress (Chen et al., 2010; Orme et al., 2001). Our subcellular localization predictions for GbDnaJ proteins indicated that the majority are localized to the nucleus, cytoplasm, and chloroplasts, suggesting their potential roles in the response of *Gossypium barbadense* to abiotic stresses.

### 4.2 Phylogenetic analysis of the *GbDnaJ* gene family

The phylogenetic relationships of gene family members help elucidate their evolutionary history, classification, functional prediction, as well as processes of adaptation and speciation in organisms and genes (Li et al., 2024). Early J-proteins were classified into three types (Type A, B, and C) based on the bacterial DnaJ classification (Georgopoulos et al., 1980), all possessing the J-domain for interaction with HSP70. Subsequent research on *Arabidopsis thaliana* revealed that some activities of DNAJ proteins do not require the J-domain, leading to the expansion of DnaJ types into three new categories (Type D, E, and F). DnaJD contains a J-like domain and comprises 15 members. DnaJE, the largest and most complex component of DnaJ-related proteins, consists of 33 members, all containing a zinc-finger domain similar to DnaJA. DnaJF possesses C-terminal domains resembling those of DnaJA/B but lacks both the J-domain and the zinc-finger domain, with three members identified in *Arabidopsis thaliana.* Consistent with previous studies, this study identified 109 GbDnaJ proteins and classified them into five types based on phylogenetic analysis, with Type V being the most abundant (**Figure 1**). Types I and IV showed a close evolutionary relationship, indicating their relative conservation in evolution and function. 10 conserved motifs were identified among the 109 GbDnaJ proteins (**Figure 5**). All DnaJ proteins contained motif 1, while the vast majority contained motifs 2 and 6. In contrast, motif 7 was found only in a few members of Type III. The classification of *GbDnaJ* members into several subgroups based on conserved domains suggests that they may perform diverse functions.

### 4.3 Duplication events of *GbDnaJs* during evolution

Gene duplication serves as a critical mechanism in evolution, facilitating organismal adaptation to environmental changes during growth and development, and is an essential driver of gene evolution and expansion (Magadum et al., 2013; Clark, 2023). Gene duplication provides additional genetic material for functional innovation, enabling plants to better cope with environmental stresses. Among various duplication mechanisms, tandem duplication is a form of genomic DNA replication and rearrangement, whitch constitutes a major factor in gene family expansion (Liang and Schnable, 2018). In the genomes of *Arabidopsis thaliana* and *Oryza sativa*, 15–20% of genes are organized in tandem repeats and gene clusters, which are considered vital for evolution, enhanced disease resistance, and improved responses to abiotic stresses (Hanada et al., 2018; Ji et al., 2024). Following duplication, duplicated copies can evolve novel functions or specialize in specific stress responses, thereby equipping plants with a broader repertoire of adaptive mechanisms. In the present study, chromosomal localization and gene structure analyses revealed that gene duplication events have occurred during the expansion and evolution of the *Gossypium barbadense* genome. Chromosomal localization demonstrated that all *GbDnaJ* genes are unevenly distributed across the 26 chromosomes of *Gossypium barbadense* (**Figure 1**). Gene duplication events included two tandem duplication events and 468 segmental duplication events. These findings suggest that segmental duplication likely served as the primary mechanism for the expansion of the *GbDnaJ* gene family during its evolution. These duplications have contributed to the functional expansion and diversification of *GbDnaJ* genes, enabling them to adapt to diverse environmental challenges.

### 4.4 *GbDnaJ* genes play important roles in heat stress and fiber development of *Gossypium barbadense*

Promoters are crucial regulatory elements that control the initiation and level of gene expression. Studying promoters is essential for understanding organismal growth, development, and defense systems (Andersson and Sandelin, 2020). Transcriptional regulation of genes can be influenced by cis-acting elements within the promoter region that mediate responses to various stimuli (Yang et al., 2012). To investigate the biological functions of *GbDnaJ* genes, this study analyzed the cis-regulatory elements in the promoters of *GbDnaJ* family genes (**Figure 8**). The results indicated that the types of cis-acting elements vary among individual *GbDnaJ* genes, primarily including responses to light, plant growth and development, stress, and hormones such as auxin (IAA), abscisic acid (ABA), jasmonic acid (JA), and gibberellin (GA). Therefore, GbDnaJ genes are likely involved in stress responses regulated and fiber development by multiple hormones.

Cotton fiber development is a complex biological process that involves multiple stages including fiber initiation, elongation, secondary wall synthesis, and maturation. The initiation mechanisms of lint and fuzz fibers involve genes such as MYB transcription factors (TFs) (Walford et al., 2011; Zang et al., 2022) and phyto-hormones such as auxin (Davière and Achard, 2016; Chen et al., 2021a; Mishra et al., 2022), ABA (Davière and Achard, 2016; Niu and Fu, 2022), GA (Beasley et al., 1974; Zang et al., 2022), brassinosteroid (BR) (Hu et al., 2016; Manghwar et al., 2022) and ethylene (ETH) (Zhang et al., 2011; Mishra et al., 2022). Our analysis revealed that multiple *GbDnaJ* genes contain cis-regulatory elements associated with hormone signaling pathways that are known to regulate fiber development which suggests the potential involvement in fiber initiation and elongation of *GbDnaJs*.

Cis-acting regulatory elements largely determine tissue-specific gene expression patterns. In this work, we examined the expression profiles of *GbDnaJ* genes in different tissues, including flowers, buds, and leaves. The results showed significant upregulation of 5 genes and downregulation of 4 genes in flowers; upregulation of 7 genes and downregulation of 6 genes in buds; and upregulation of 4 genes and downregulation of 7 genes in leaves. Some *GbDnaJ* genes exhibited tissue-specific expression. For instance, *GbDnaJ56* showed high expression levels in flowers but low levels in other tissues, whereas *GbDnaJ54* was highly expressed in buds and leaves but low in flowers. Combined with the analysis of cis-regulatory elements, we found that *GbDnaJ54* contains numerous ABRE and G-box elements, suggesting it might be strongly activated by ABA signaling and thereby participate in the response to heat stress. Conversely, *GbDnaJ56* contains GA-responsive elements, potentially functioning in promoting floral organ elongation and anther development. These results demonstrate that *GbDnaJ* genes play diverse roles in plant growth and development.

Within the *GbDnaJ* genes family, 72% of the members possess ABRE elements. ABRE elements respond to ABA signals under stress conditions by activating the expression of genes in the ABA pathway, mediating the transcription of downstream target genes, thereby enabling plants to adapt to and withstand adverse environments. They play a key role in coordinating responses to reduced water availability and other environmental factors, as well as in various developmental processes (Fujita et al., 2013). In addition, the cis-acting element analysis revealed that *GbDnaJ* promoters are enriched with other phytohormone-responsive elements, including AuxRE (58), P-box (31), and MeJA-responsive elements (117). Notably, these hormones are key regulators of cotton fiber development: auxin and gibberellin promote fiber initiation and elongation, while ABA acts as a negative regulator (Beasley et al., 1974; Davière and Achard, 2016; Mishra et al., 2022).

Similarly, jasmonic acid (JA) plays an important role in plant adaptation to high light and high-temperature stress. As a major plant growth regulator, JA participates in numerous signal transduction pathways, involving gene networks, regulatory proteins, signaling intermediates, enzymes, proteins, and other molecules that protect cells from the detrimental effects of various environmental stresses (Raza et al., 2021). For example, in *Arabidopsis thaliana*, the expression of many transcripts involved in JA biosynthesis is upregulated under high-temperature and high-light stress. JA-deficient mutant plants are more sensitive to high-temperature stress, exhibiting significantly reduced survival rates (Balfagón et al., 2019). In pea plants pretreated with high concentrations of methyl jasmonate (MeJA) (50, 100, and 200 µM) under heat stress, morphological and physiological functions were impaired, while JA content was upregulated. Treatment of *Aquilaria sinensis* cell suspension cultures with heat stress significantly upregulated the expression of genes related to JA biosynthesis (Shahzad et al., 2015). High temperature can suppress the expression of the JA biosynthesis gene allene oxide cyclase 2 (*GhAOC2*), reducing JA content and leading to male sterility in the heat-sensitive cotton line H05. Exogenous application of MeJA at the early bud stage can improve male fertility in cotton under heat stress (Khan et al., 2023). In this study, we found that 59% of *GbDnaJ* genes contain JA-related elements (CGTCA-motif and TGACG-motif), indicating their potential involvement in the heat response of *Gossypium barbadense*.

We also observed that 32.5% of *GbDnaJ* genes contain anaerobic response elements (ARE), which are associated with reactive oxygen species (ROS) signaling. ROS serve as critical signaling molecules during cotton fiber initiation and elongation (Jan et al., 2022). Fiber development in *Gossypium barbadense* occurs predominantly during summer, when temperatures frequently exceed 35°C, coinciding with the critical period of fiber elongation (5–20 days post-anthesis). Our physiological data demonstrated that ascorbate peroxidase (APX) activity was significantly elevated in flowers and leaves under heat stress. APX is a key enzyme for ROS scavenging, which participates in ROS homeostasis regulation related to fiber development. As molecular co-chaperones, GbDnaJ proteins may protect APX and other ROS-scavenging enzymes from heat-induced denaturation, thereby maintaining the precise ROS balance required for normal fiber development. Furthermore, 19.3% and 17.8% of *GbDnaJ* family members contain As-1 (Activating Sequence-1) and LTR (Low Temperature Responsive) elements, respectively. These results suggest that *GbDnaJs* can respond sensitively to heat stress signals and play important roles in stress responses by interacting with corresponding transcription factors.

In summary, these findings provide a comprehensive analysis of *GbDnaJs*, and provided reference for improving fiber quality under heat stress conditions.

## 5 Conclusion

DnaJ proteins play crucial roles in protein translation, folding, unfolding, translocation, and degradation. In this study, 109 *GbDnaJ* genes were identified and systematically characterized through bioinformatic analysis and expression profiling. These genes were distributed across all 26 chromosomes. Phylogenetic analysis showed that these GbDnaJ proteins cluster into five distinct types, with similar gene structures in each type, reflecting functional conservation. Cis-acting regulatory elements analysis indicated that this gene family can respond to multiple hormonal signaling pathways, and may be involved in cotton fiber development. Synteny analysis suggested that these genes have likely undergone whole-genome duplication events and large-scale segmental duplications. The protein interaction predictions with GbaHSP70 revealed that HSP70 proteins exhibited significant centrality in the network, whereas DnaJ proteins demonstrated high clustering coefficients. The core interaction module (GbDnaJ1-GbaHSP70-21) provides targets for future research on molecular breeding for thermotolerance in *Gossypium barbadense*. Gene expression analysis demonstrated that under heat stress, 15 GbDnaJ genes showed significant induction in flowers, buds, or leaves, indicating their important roles in the growth and response of *Gossypium barbadense* to heat stress. In summary, these results provide a comprehensive analysis of *GbDnaJs* and serve as a valuable reference for future research on thermotolerance in *Gossypium barbadense*. In addition, these results provide a valuable resource for further investigation of *DnaJ* gene function in *Gossypium barbadense*.

## Data Availability

The data presented in the study are deposited in the NCBI repository, accession number “PRJNA1392845". Further inquiries can be directed to the corresponding author/s.

## Supplementary material

Supplementary Data Sheet 1 Protein sequences of the *GbdnaJ* gene family.

Supplementary Data Sheet 2 Cis-Acting regulatory elements drawing code.

Supplementary Data Sheet 3 Heatmap drawing code.

Supplementary Table 1 Primer sequences used for qRT-PCR analysis of *GbDnaJ*. The table includes gene identifiers, primer forward and reverse sequences, amplicon size (bp), and melting temperature (Tm).

Supplementary Table 2 Transcriptome expression profiles (TPM values) of *GbDnaJ* genes. This table provides TPM values for each gene across biological replicates, and statistical significance (padj) for comparisons between heat-stressed and control conditions(CK) in flowers, buds and leaves.

Supplementary Table 3 Meteorological data on the sampling day. This table provides the meteorological data from the sampling date.

Supplementary Table 4 Sequences of the GbaHSP70s. This table provides the HSP70 protein sequences of *Gossypium barbadense* which derived from previously reported studies.
