# Supplementary material for: Genome-wide identification and expression profiling of DnaJ gene family in Gossypium barbadense reveals candidate thermotolerance genes
Source: Front Plant Sci. 2026 Jan 20;16:1728216. doi: 10.3389/fpls.2025.1728216 (PMC12865410; doi:10.3389/fpls.2025.1728216)
Supplement: Supplementary Data Sheet 1 — Protein sequences of the GbdnaJ gene family. [file Supplementaryfile1.zip › Supplementary Material/Data Sheet 1.PDF]

>GbDnaJ1
MRCSYGLTAIPGTDARFFSPETSFLQLRRTFQPTRIKFGSFKIKAKIDEARKEMSFYELL
GISERGTSLEIKQAYKQLARKYHPDVSPPDRVEEYTERFIRVQEAYETLSDPRRRALYDK
DLALGIHLAFSARRRYQYDEDLEDRSEWKNRWQSQLSELKRRSRNRDAGGNMSWGARMRR
QREELSKEL
>GbDnaJ2
MRVDWDDLPEEPQQPEQDSSLNFEFFSLVSKPKDYYKILEVDYDATEDAIRSNYIRLALK
WHPDKQKDDGDSATSRFQEINEAYQVLIDPVKRSEYDKKGMLHVYDYDIIEYLNRYKGLI
LTCNGLGIRQSTW
>GbDnaJ3
MVKETEYYDVLGVSPSASVEEIRKAYYLKARQVHPDKNPNDPHAAERFQVLGEAYQILSD
PVQRDAYDRNGKYSISRDTMLDPTAVFALLFGSELFEDYIGHAAAASMASSELANETDNP
EKLNDKLKAVQKEREEKLAKSLRDSLNQYVRGDKDGFIQHAESEARRLSDTAFGVDILHT
IGYIYSRQASQELGKKAIYLGVPFLAEWVRHKGHFWKSQITAAKGAFQLLQLQEDMRKQF
KMDGSGAENDVEAHLRLNKETLMSSLWKLNVVDIEVTLVHVCQMVLRENNVKKEELKARA
LALKILGKTFQEQEARNGGTSRRKNVAEMDDDDDGSSSDSSSEEESPGALSYRTPFLTQG
IGRLFRCLCNPAFDVDDDEIVFKSK
>GbDnaJ4
MEGNDNSTSKDYYKILEVDYDATDENIRLSYRKLALKWHPDKHKGDSAVTAKFQEINEAY
NVLIDPDKRFEYDLTGIYEIDKYTLREYLARFKGMILTCNGLGISHTST
>GbDnaJ5
MEGNDNSTSKDYYKILEVDYDATDENIRLSYRKLALKWHPDKHKGDSAVTAKFQEINEAY
NVLIDPDKRFEYDLTGIYEIDKYTLREYLARFKGMILTCNGLGISHTSTWTEQLTDRNEF
AEEG
>GbDnaJ6
MAIIPCGSTFVAQWHIRPQLTTRSYVPNRIMTARLGVTSTMSYLRASNSGLFARDSLPLL
SFVGPSQTSHHRRGARFIVRAETDYYTVLGVSRNASKSEIKSAYRKLARSYHPDVNKDPG
AETKFKEISNAYEVLSDDEKRSLYDKYGEAGLKGAGMGVGDFSNPFDLFESLFEGMGGMG
GMGGMGMGGRSSRNRAVDGQDEYYSLVLNFKEAVFGVEKEIEITRLESCGTCNGSGAKPG
TTPSKCTTCGGQGQVISSARTPLGVFQQVMTCSSCGGTGEISTPCNTCSGDGRVRRTKRI
SLKVPAGVDSGSRLRVRSEGNAGRRGGSAGDLFVVIEVIPDPVLKRDDTNILYTCKVSYI
DAILGTTIKVPTVDGMVDLKIPAGTQPNTTLVMAKKGVPVLNKTNMRGDQLVRVQVEIPK
RLSSEEKKLIEELADLSKGKTASSRR
>GbDnaJ7
MRCSYGLTAIPGTDARFLSPETSFLKLRRTFQPTRIKFGSFKIKAKIDEARKEMSFYELL
GISETGTSLEIKQAYKQLARKYHPDVSPPDRVEEYTERFIRVQEAYETLSDPRTRALYDK
DLALGIHLAFSARRRYQYDEDLEDRSEWKNRWQSQLSELKRRSMNRDAGGNMSWGARMRR
QREELSKEL
>GbDnaJ8
MATSYPLYNCGTRQNSFLIPGSGSRTSVLGFRWSESQFFCKTHAPSYRLDIIKRKCRHNA
VIRAAASGSDYYSTLNVSRGATLQEIKTSYRSLARKYHPDINKTPGAEEKFKEISAAYEV
LSDNEKRSLYDRFGEAGLQGEYDGSGDYSAAVDPFEVYNAFFGGSDGFFSGMGEPGGFNF
NLRNNGSNDLDIWYELHLNFEESIFGGEREIMVSYLETCNDCGGTGAKTSSCVKSCTDCG
GKGGSTKSKRTPFGVAIEVSTCSSCGGKGKIITDKCRRCSGCCKVKVKRSMRIIIPPGVA

DGFTKRIRGEGNVDKKSGFAGDLFVVLRIGAKQGIWRDGLNLYSKINVDYTEAILGTVVK
VETVEGIKDLRIPCGIQPGDAVKLSRLGVPDVNKPSVRGDHHFFVNVLIPKDISSIPIFT
RVARH
>GbDnaJ9
MATSYPLYNCGTRQNSFLIPGSGSRTSVLGFRWSESQFFCKTHAPSYRLDIIKRKCRHNA
VIRAAASGSDYYSTLNVSRGATLQEIKTSYRSLARKYHPDINKTPGAEEKFKEISAAYEV
LSDNEKRSLYDRFGEAGLQGEYDGSGDYSAAVDPFEVYNAFFGGSDGFFSGMGEPGGFNF
NLRNNGSNDLDIWYELHLNFEESIFGGEREIMVSYLETCNDCGGTGAKTSSCVKSCTDCG
GKGGSTKSKRTPFGVAIEVSTCSSCGGKGKIITDKCRRCSGCCKVKVKRSMRIIIPPGVA
DGFTKRIRGEGNVDKKSGFAGDLFVVLRIGAKQGIWRDGLNLYSKINVDYTEAILGTVVK
VETVEGIKDLRIPCGIQPGDAVKLSRLGVPDVNKPSVRVTKNANLLRK
>GbDnaJ10
MLPHRSKSEKNDGMAKQLRRDPYEVLGVSRNSTDQEIKSAYRKMALKYHPDKNGNDPVAA
DMFKEVTFSYNILSDPDKRHQYDTAGFEAVEAENQELELDLSSLGAVNTMFAALFSKLGV
PIKTTVSATVLEEALNGVVTIQTLLLGQPVSRKVEKQCAHFYSVEITEEEARDGFVCRVQ
SSDKSKFKLLYFDLEENGGLSLALQEDSAKTGKVTSAGMYFLGFPVYRLDQTVNSVAAAK
DPDTAFFKKLDGFQPCEITELKPGTHFFAVYGDNFFKSVSYTIEAICTAPFIEEKENLRA
VEAEILSKRVELSKFETEYREVLAQFTEMTTRYTKEMQEIDELLKQRNEIHASYTMIPPS
KRSSSRSRSKGVSREAKEGEVRDRKHSTRDRTKKKRWYNIHLKIDKRKQPC
>GbDnaJ11
MLPHRSKSEKNDGMAKQLRRDPYEVLGVSRNSTDQEIKSAYRKMALKYHPDKNGNDPVAA
DMFKEVTFSYNILSDPDKRHQYDTAGFEAVEAENQELELDLSSLGAVNTMFAALFSKLGV
PIKTTVSATVLEEALNGVVTIQTLLLGQPVSRKVEKQCAHFYSVEITEEEARDGFVCRVQ
SSDKSKFKLLYFDLEENGGLSLALQEDSAKTGKVTSAGMYFLGFPVYRLDQTVNSVAAAK
DPDTAFFKKLDGFQPCDNFFKSVSYTIEAICTAPFIEEKENLRAVEAEILSKRVELSKFE
TEYREVLAQFTEMTTRYTKEMQEIDELLKQRNEIHASYTMIPPSKRSSSRSRSKGVSREA
KEGEVRDRKHSTRDRTKKKRWYNIHLKIDKRKQPC
>GbDnaJ12
MGVDYYNILKVSRNATDDDLRKSYKRLARKWHPDKNLVNNKEAEAKCKQIFEAYNVLSDP
LKRQIYDLHGEQGLNSAESSSPNGFSAGGVGGMADKFDQRNGQGYKKASPVETQLLCSLE
ELYKGGRRRMRISRSIPGEFGKLKTVEEILKIDIKPGWKKGTKITFPEKGNQEPGFTPSD
LIFVVDEKPHAIFKRDGNDLIATLKISLLEALTGTILSLTTLDGRTLPISVTDIVNPGHE
VVIPNEGMPISKEPSKRGHLKIQFDIIFPSKLSAEQKCDLRRALSQR
>GbDnaJ13
MGVDYYNVLQVDRNATEDDLKKAYRKLAMKWHPDKNPNNKKEAEANFKRISEAYEVLSDP
QRRAIYNQHGEEGLKDVPPPGSSGPSYGNGTGGPNGFNPRNAEDIFAEFFGSSPFGFGST
GPGRSSRFQSEGGKFGGGFGCTDNNFRSYNDTTAPRKPPPVESKLPCTLEELYTGSTRKM
KISRTIVNASGRQAQESEILTIDVKPGWKKGTKITFPDKGNEQPNQLPADLVFVIDEKPH
DLYKRDGNDLIVNNRVSLAEALGGTTINLTTLDGRNLSLSVTDIINPGYELVVAREGMPI
AKEPGNRGDLKIKFDVKFPTRLTPEQQAGLKRALGG
>GbDnaJ14
MAATTSLSLLPSSLGFPNERPSSTSQSSSYSCSCSVFFNAGTRLRSHDSFACVTFPSSSS
TCSWRFNNRAGTHRFGTTVVAASGDYYATLGVPKSASGKEIKAAYRRLARQYHPDVNKEP
GATEKFKEISAAYEVLSDDKKRALYDQYGEAGVKSAVGGQSSAYTTNPFDLFETFFGPSM

GGFPGMDQTGFGTSRRSTVSKGDDIRYDITLEFSEAIFGAEKEFELSHLETCEVCLGTGA
KVGSKMRICSTCGGRGQVMRTEQTPFGLFSQVSVCPNCGGDGEVISENCRKCSGKGRVRV
KKNIKVKVPPGVSAGSILRVAGEGDAGPKGGPPGDLYAYLDVQEVPGIQRDGINLLSTVS
ISYLDAILGSVVKVKTVEGVTDLQIPPGTQPGDVLVLARKGAPKLNKPSIRGDHLFTIKV
NIPNRISAKERELLEELSSLSNTNGSRSRTRPRTQPATATKTSGSKVSTDGEKTEAAAAD
ENDTWTKLKKFAGSIANGVAKWLKDNL
>GbDnaJ15
MAATTSLSLLPSSLGFPNERPSSTSQSSSYSCSCSVFFNAGTRLRSHDSFACVTFPSSSS
TCSWRFNNRAGTHRFGTTVVAASGDYYATLGVPKSASGKEIKAAYRRLARQYHPDVNKEP
GATEKFKEISAAYEVLSDDKKRALYDQYGEAGVKSAVGGQSSAYTTNPFDLFETFFGPSM
GGFPGMDQTGFGTSRRSTVSKGDDIRYDITLEFSEAIFGAEKEFELSHLETCEVCLGTGA
KVGSKMRICSTCGGRGQVMRTEQTPFGLFSQVSVCPNCGGDGEVISENCRKCSGKGRVRV
KKNIKVKVPPGVSAGSILRVAGEGDAGPKGGPPGDLYAYLDVQEVPGIQRDGINLLSTVS
ISYLDAILGSVVKVKTVEGVTDLQIPPGTQPGDVLVLARKGAPKLNKPSIRGDHLFTIKV
NIPNRISAKERELLEELSSLSNTNGSRSRTRPRTQPATATFTYSCSQN
>GbDnaJ16
MFGRAPKKSDNSKYYEILGVSKNASQDDLKKAYRKAAIKNHPDKGGDPEKFKELAQAYEV
LSDPEKREIYDQYGEDALKEGMGSGGGGHDPFDIFQSFFGGNPFGAGGSSRGRRQRRGED
VIHPLKVSLEDLYNGTSKKLSLSRNVICSKCKGKGSKSGASMKCSGCQGSGMKVSIRHLG
PSMIQQMQHPCNECKGTGETINDKDRCPQCKGDKVVQEKKVLEVIVEKGMQNGQRITFPG
EADEAPDTVTGDIVFVLQQKDHPKFKRKGDDLFVEHTLALTEALCGFQFILTHLDGRQLL
IKTQPGEVVKPDQFKAINDEGMPIYQRPFMRGKLFIHFTVDFPDSLTPDQCKALEAVLPP
KASVQLTDMELDECEETTMHDVNIEEEMRRKQAQAQQEAYEEDEDMHGGAQRVQCAQQ
>GbDnaJ17
MVKETDFYDILGVSPSATESEIKKAYYMKARQVHPDKNPNDPQAAQNFQVLGEAYQVLSD
PAQRQAYDAHGKAGISTEAIIDPAAIFAMLFGSELFEEYIGQLAMASMASLDIFTEGEQF
DAKKVQEKMKVVQKEREDKLAQLLKDRLNQYVQGNKADFVNHAEAEVSRLSSAAYGVDML
NTIGYIYARQAAKELGKKAIYLGVPFVAEWFRNKGHFIKSQVTAATGAIALLQLQEDMKK
QLSAEGNYSEEELEEYMQSHKKLMIDSLWKLNVADIEATLSRVCQMEELRARAKGLKTLG
KIFQSVKSTNGNESDPVLGNNARHKLDGVEPSYNSGSPNVSTKSSSREELSPSPLAPQSP
YVEAPNFVNAQLPRPTAPPGAQRHP
>GbDnaJ18
MVKETDFYDILGVSPSATESEIKKAYYMKARQVHPDKNPNDPQAAQNFQVLGEAYQVLSD
PAQRQAYDAHGKAGISTEAIIDPAAIFAMLFGSELFEEYIGQLAMASMASLDIFTEGEQF
DAKKVQEKMKVVQKEREDKLAQLLKDRLNQYVQGNKADFVNHAEAEVSRLSSAAYGVDML
NTIGYIYARQAAKELGKKAIYLGVPFVAEWFRNKGHFIKSQVTAATGAIALLQLQEDMKK
QLSAEGNYSEEELEEYMQSHKKLMIDSLWKLNVADIEATLSRVCQMVLHDQKCKKEELRA
RAKGLKTLGKIFQSVKSTNGNESDPVLGNNARHKLDGVEPSYNSGSPNVSTKSSSREELS
PSPLAPQSPYVEAPNFVNAQLPRPTAPPGAQRHP
>GbDnaJ19
MVKETEYYDVLGVSPSATEAEIKKAYYIKARQVHPDKNPNDPLAAQNFQVLGEAYQVLSD
PTQRQAYDAYGKSGISAEAIIDPAAIFAMLFGSELFEEYIGQLAMASMASLDIFTEGEQV
DPKKLQEKMKVVQKEREEKLAQILKDRLNQYVQGSKEDFANHAEAEVSRLSNAAYGVDML
NTIGYIYARQAAKELGKKAIYLGVPFIAEWFRDKGHYIKSQVTATTGAIALLQLQEEMKK

QLNAEGNYTEEELEEYMQSHKKILTDSLWKLNVADIEATLSRVCQMVVSLVSSFTAVR

>GbDnaJ20

MVKETEYYDVLGVSPSATEAEIKKAYYIKARQVHPDKNPNDPLAAQNFQVLGEAYQVLSD
PTQRQAYDAYGKSGISAEAIIDPAAIFAMLFGSELFEEYIGQLAMASMASLDIFTEGEQV
DPKKLQEKMKVVQKEREEKLAQILKDRLNQYVQGSKEDFANHAEAEVSRLSNAAYGVDML
NTIGYIYARQAAKELGKKAIYLGVPFIAEWFRDKGHYIKSQVTATTGAIALLQLQEEMKK
QLNAEGNYTEEELEEYMQSHKKILTDSLWKLNVADIEATLSRVCQMVLQDGNCKREELRA
RAKGLKTLGRIFQRAKSANGSESETVESSTVHTLDGSELSYDSSSLNASSRSLNQEELSH
STFASQSPYVEAPNFVDTQFTYNFPRPTAPPGAQRTSLN

>GbDnaJ21

MTSDTSYYDILGVKVSASAAEIKKAYYLKARQVHPDKNPGDPKADEKFAALSEAYQVLSD
PDKREHYDKNGKDGIIPGSMLDPSAVFGMAFGSDYFDEYVGTLAMATLSSLEVEFEESSV
DKEARTQKIREKMEVLQKEREDKLIVTLKNRLQPFVDGQTDEFIYWANSEAQRLSKAAFG
EAMLHTIGYIYIRKGASELGKDKRYMKVPFIAEWVRDKGHRVKSQVMAASGAVSLIQIQE
ELKKVNQGEKKDENLMKALEDKREAMLQSLWKVNVVDIETTLSRVCLAVLQDPDASKDVL
ILRAKALKKLGSIFQVCSSE

>GbDnaJ22

MTSDTSYYDILGVKVSASAAEIKKAYYLKARQVHPDKNPGDPKADEKFAALSEAYQVLSD
PDKREHYDKNGKDGIIPGSMLDPSAVFGMAFGSDYFDEYVGTLAMATLSSLEVEFEESSV
DKEARTQKIREKMEVLQKEREDKLIVTLKNRLQPFVDGQTDEFIYWANSEAQRLSKAAFG
EAMLHTIGYIYIRKGASELGKDKRYMKVPFIAEWVRDKGHRVKSQVLLV

>GbDnaJ23

MTSDTSYYDILGVKVSASAAEIKKAYYLKARQVHPDKNPGDPKADEKFAALSEAYQVLSD
PDKREHYDKNGKDGIIPGSMLDPSAVFGMAFGSDYFDEYVGTLAMATLSSLEVEFEESSV
DKEARTQKIREKMEVLQKEREDKLIVTLKNRLQPFVDGQTDEFIYWANSEAQRLSKAAFG
EAMLHTIGYIYIRKGASELGKDKRYMKVPFIAEWVRDKGHRVKSQVMAASGAVSLIQIQE
ELKKVNQGEKKDENLMKALEDKREAMLQSLWKVNVVDIETTLSRVCLAVLQDPDASKDVL
ILRAKALKKLGSIFQGVKVRYSREDSLRHEIDC

>GbDnaJ24

MFGRVPKKSDNTKFYEVLGVPKSASQDELKKAYKKAAIKNHPDKGGDPEKFKELAHAYEV
LSDPEKREIYDQYGEDALKEGMGGGGSSHNPFDIFESFFGGGAFGGGGSSRGRRQKRGED
VVHALKVSLEELYNGTTKKLSLSRNAICSKCKGKGSKSGASSRCYGCQGTGMKITTRQIG
LGMIQQMQHVCPECRGSGEMISDRDRCPQCKGNKVTQEKKVLEVHIEKGMRDGQKITFEG
QADEAPDTITGDIVFVMEQKKHPKFDRRFGDELYVDHKLSLTEALCGFQFALTHLDGRQL
LIKSNPGEVVKPGQYKAINDEGMPHHQRPFMKGKLVIQFHVIFPESGVFSPEQCRKIESV
LPMRPSKHLTDMELDDCEETTLHDVSAEEVKRRKEQRQHRHREAYDEDDDDDESAPRVQC
AQQ

>GbDnaJ25

MALPRSKLLFLLFALSFAIVAIAGKSYYDILQVPKGASDEQIKRAYRKLALKYHPDKNPG
NEEANKRFADINNAYEVLSDSEKRGIYDRYGEEGLKQHATSGGRGGMGVNIQDIFSSFFG
GGTVEEEERIVKGDDVIVELDATLEDLYMGGTLKVWREKNILKPAPGKRRCNCRNEVYHK
QIGPGMFQQMTEQVCEQCQNVKYEREGYFVTVDIEKGMQDGQEVVFYEDGEPIIDGEPGD
LKFRIHTAPHDRFRREGNDLHTTVTITLVQALVGFDKTIKHLDDHLVEIGSKGITKPKEV
RKFKGEGMPLHFSNKKGDLFVTYEVLFPTSLAEDQKAKIKSILG

>GbDnaJ26
MALPRSKLLFLLFALSFAIVAIAGYASISPVSLSLKKCVHICSWWCFCCRKSYYDILQVP
KGASDEQIKRAYRKLALKYHPDKNPGNEEANKRFADINNAYEVLSDSEKRGIYDRYGEEG
LKQHATSGGRGGMGVNIQDIFSSFFGGGTVEEEERIVKGDDVIVELDATLEDLYMGGTLK
VWREKNILKPAPGKRRCNCRNEVYHKQIGPGMFQQMTEQVCEQCQNVKYEREGYFVTVDI
EKGMQDGQEVVFYEDGEPIIDGEPGDLKFRIHTAPHDRFRREGNDLHTTVTITLVQALVG
FDKTIKHLDDHLVEIGSKGITKPKEVRKFKGEGMPLHFSNKKGDLFVTYEVLFPTSLAED
QKAKIKSILG
>GbDnaJ27
MALPRSKLLFLLFALSFAIVAIAGKSYYDILQVPKGASDEQIKRAYRKLALKYHPDKNPG
NEEANKRFADINNAYEVLSDSEKRGIYDRYGEEGLKQHAASGGRGGMGVNIQDIFSSFFG
GGSVEEEERIVKGDDVIVELDATLEDLYMGGTLKVWREKNILKPAPGKRRCNCRNEVYHK
QIGPGMFQQMTEQVCEQCQNVKYEREGYFVTVDIEKGMQDGQEVVFYEDGEPIIDGEPGD
LKFRIHTAPHDRFRREGNDLHATVTITLVQALVGFDKTIKHLDDHLVEIGSKGITKPKEV
RKFKGEGMPLHFSNKKGDLFVTYEVLFPTSLAEDQKAKIKSILG
>GbDnaJ28
MDREGGSHGGSCYYSVLGIRKDASFSDIRTAYRKLAMKWHPDRYARNPGIAGEAKRRFQQ
IQEAYSVLSDESKRSMYDAGLYDPLEEEDQDFCDFMQEMMSMMNNVKDEGDSFEDLQRMF
AEMVDGISFDINTDPTVTKTARVTASKGNAARRNSSRC
>GbDnaJ29
MGLDYYKILKVSSNVNDEDLKKAYKRLAMKWHPDKNPLNNEDAEAKFKQIVEAYEVLSDP
QKRAVYDRYGEEGLKGVAPPAESGGASFFSTGYGIPTTFQFNQQNVADEIFSELFGAFGG
GMRGTRFSSSLFGDDIFGGGGGGRGGVRFSGSVYGDDIFGSTNPGALRKAAPMENWLHCS
LEELFKGTTKKMKICREIVHISGKIMQVEEILTINVRPGWKKGTKITFAEKGNERPNIIP
ADLIFIIEEKPHNVFTREGNDLIVAQKISLVDALYGCTVHLTTLDGRNLTIPIHNVIHPC
YEEVVPREGMPIQKDPSKRGNLRIKFDVKFPTRLTPEQKSGIKKLLGP
>GbDnaJ30
MGKRKKSRVSRDEDEEDEVVQEEEGNHSSSNEKSLYEILGVEKAASQQEIKKAYYKLALR
LHPDKNPGDEEAKEKFQQLQKVISILSDEEKRAVYDQTGCVDDTDLAGDVVENLKSFFRT
MYKKVTDADIEEFEANYRGSDSEKKDLIDLYRKFKGNMNKLFCSMLCSDPKLDSHRFKDM
LDEAIAAGELKESKAYKKWAKKVAEMKPPTSPLRRKGKSNKQPESDLFAIISQRRNERKD
RFDSMFSSLVSKYGGNAESEPTEEEFEAAKRKVESKKASNNSKRKRQGKR
>GbDnaJ31
MGKRKKSRVSRDEDEEDEVVQEEEGNHSSSNEKSLYEILGVEKAASQQEIKKAYYKLALR
LHPDKNPGDEEAKEKFQQLQKVISILSDEEKRAVYDQTGCVDDTDLAGDVVENLKSFFRT
MYKKVTDADIEEFEANYRGSDSEKKDLIDLYRKFKGNMNKLFCSMLCSDPKLDSHRFKDM
LDEAIAAGELKESKAYKKWAKKVAEMKPPTSPLRRKGKSNKQPESDLFAIISQRRNERKD
RFDSMFSSLVSKYGGNAESEPTEEEFEAAKRKVESKKASNNSKRKRQ
>GbDnaJ32
MGKRKNSGVSRDEEEEVEVENHSSSNEKSLYEVLNVAKTASQQEIKKAYYKLALRLHPDK
NPGDEEAKEKFQQLQKVISILGDEEKRAVYDQTGCVDDADLAGDVVENLKTFFRAMYKKV
TEADIEEFEVNYRGSDSEKKDLFDLYKKCKGNMNKLFCSMLCSDPKLDSHRFKDLLDEAI
AAGELKETKAYGKWANKVSEMKPPTSPLRRKEKSVKQSESDLLAIISQRRSERKDRFDSM
FSTLVSKYGGNADSEPTEEEFEAARRKVESRKASNKSKHKLHQALF

>GbDnaJ33
MLKSLSLNWAFNSMAWKGLVYTAFILNFVLICQHLLLQPLVSALDENLGNAAELFERVSQ
SIKVKRYSEALNDLNAAIEADPALSEAYMRRASLLRQLCRYEESEKSYKKFLELKPRNSV
AEKELSQLHQAQSALETAFGLFESKDYTKALDYVDKVVLIFSPACSKAKMLKLKLLLAAK
DYSSVISESGFILKEDENNLEALLLRGHAYYYLADHDVAQRHYQKGLRLDPEHGELKKAY
FRLKNLLKKTKSAEDNANKGKLRVAVEDYKGALALDPNHLAHNVHLHLGLCKVLVRLGRG
KDALSSCSEALNIDKELLEALVQRGEAKLLTEDWEGAVEDLKSAAEKSPQDMNIREALMR
AEKALKMSKRKDWYKILGVSKTASVAEIKRAYKKLALQWHPDKNVDNREEAEAQFREIAA
AYEVLGDEEKRTRYDRGEDIEDIGMGGGGFNPFGGGGGGQHFTFTFDGGFPGGFGGGFPG
GGGGGFGFNF
>GbDnaJ34
MGVDYYKILQVDRNAKDEDLKKAYRKLAMKWHPDKNPNNKKDAEAKFKQISEAYDVLSDP
QKRAVYDQYGEEGLKGQMPPPGAGGFGGGYSGGPTTFRFNTRNPDDIFSDFFGFSSPFGM
GDMGGPEVATRFPRGMFSEDIFGSFRGGAGEGSTTMPRKGAPIDRPLPCSLEDLYKGTTK
KMKISRDVMDGSGRPTTVEEILTIDIKPGWKKGTKITFPEKGNEQRGVIPSDLVFIIDEK
PHSVFKRDGNDLIVTQKISLVEALTGYTAQLITLDGRNLTVPINNIISPTYEEVVKGEGM
PIPKDPSKKGNLRIKFNIKFPTKLTAEQKTGIKRLIASP
>GbDnaJ35
MGVDYYNVLKVEKNATDDDLKKSYRKLAMKWHPDKNPNDKKEAETKFKQISEAYEVLSDP
QKRAVYDQYGEEGLKDMPPPGSSRPPFGKGTGGPNGFNPRNAEDIFAEFFGSSPFGLGSS
GPTRSARFHSDGAKFGGFNSTDNIFRSHNEATAPRKPPPVESKLPCSLEELYTGSTRKMK
ISRTVVNAAGRQVQESEILTVDVKPGWKKGTKITFPDKGNEQPNQLPADLVFVIDEKPHD
LYKRDGNDLVVNQRVLLAEALGGTTVNLVTLDGRNLSLPVTDIISPGYELVIAREGMPIA
KETGNRGDLRIKFEVKFPTKLTPEQREGLKRALVGVK
>GbDnaJ36
MGVDYYKILQVDKNAKDDDLKKAYRKLAMKWRPDKNPNNKKEAEAKFKQISEAYEVLSDP
QKRAIYDQYGEEGLKGQVPPQDAGGPGGATFFQTGDGPNVFRFNPRNANDIFAEFFGYSS
PFGGMGGSGMRGSSRSFGGMFGDDIFSSFGEGRPMSQNPRKPPPIENTLPCSLEDLYKGT
TKKMKISREIADAIGKTLPVQEILTIDIKPGWKKGTKITFPEKGNEQPNTIPADLVFIID
EKPHSTFTRDGNDLVVTQKISLAEALTGYTVHLTTLDGRSLNIPINSVIHPNYEEVVPKE
GMPIPKDPSKRGNLRIKFNIKFPTRLTAEQKSGIKKLLGPSGVL
>GbDnaJ37
MFGRAPKKSDNTKYYEILGVPKTASQDDLKKAYRKAAIKNHPDKGGDPEKFKELAQAYEV
LSDPEKREIYDQYGEDALKEGMGGGGGAHDPFDIFQSFFGGNPFGGGGSSRGRRQRRGED
VIHPLKVSLEDLYNGISKKLSLSRNIICSKCKGKGSKSGASMKCSGCQGSGMKVSIRHLG
PSMIQQMQHPCNDCKGTGETINDKDRCPQCKGEKVVQEKKVLEVNVEKGMQNGQKITFPG
EADEAPDTVTGDIVFVLQQKDHPKFKRKGDDLFVEHTLTLTEAVCGFQFILTHLDGRQLL
IKTHPGEVVKPDQCKAINDEGMPMYQRHSRLCYLRGPQSS
>GbDnaJ38
MFGRAPKKSDNTKYYEILGVPKTASQDDLKKAYRKAAIKNHPDKGGDPEKFKELAQAYEV
LSDPEKREIYDQYGEDALKEGMGGGGGAHDPFDIFQSFFGGNPFGGGGSSRGRRQRRGED
VIHPLKVSLEDLYNGISKKLSLSRNIICSKCKGKGSKSGASMKCSGCQGSGMKVSIRHLG
PSMIQQMQHPCNDCKGTGETINDKDRCPQCKGEKVVQEKKVLEVNVEKGMQNGQKITFPG
EADEAPDTVTGDIVFVLQQKDHPKFKRKGDDLFVEHTLTLTEAVCGFQFILTHLDGRQLL

IKTHPGEVVKPDQCKAINDEGMPMYQRPFMRGKLYIHFTVDFPDSLAPEQCKALEAVLPS
RASVQLTDMELDECEETTLYDVNIEEEMRRKQAQAAQEAYEEDDDMHGGAQRVQCAQQ
>GbDnaJ39
MRSKKMEGTSAPSLRRDPYEVLCVSRDSSDQEIKTAYRKLALKYHPDKNANNPEASELFK
EVAYSYSILSDPEKRRQYDTAGFEAVEDSMDMEIDLSNLGTVNTMFAALFSKLGVPIKTT
ISANVLEEALNGTVTVRPLPIGTSVSGKVDKQCAHFFGVTINDEQAECGIVVRVTSTAQS
KFKLLYFEHDINGGYGLALQEDSEKTGKVTSAGMYFLHFQVYRMDSTVNALAIAKDPESA
FFKRLEGLQPCEVSELKSGTHIFAVYGDNFFKTATYTIEALCAKSYEDTTEKLKDIESQI
LRKRNELRQFETEYRKALARFQEVTNQYTQEKQSVDELLKQRDSIHATFTVTRPPSGISN
LSNGSSSKVPGETESPTEDGNSDGKDKSGKKKWFNLNLMGSDKKLG
>GbDnaJ40
MATAIPCGNTVVTGLGPRLLSSLNTNKWRISQARVSTEIEALSFSSSSIFPQNLTHLLFN
SWSPKIPCHHLARRLIVKAARDYYSILGVSKNASKSDIKSAYRKLARNYHPDVNKEAGAE
QKFKEISEAYEVLSDDEKRCIYDRYGKDGLKGSTMDMGDFTNPFDLFSSFFDMDIRNRGA
RNMAADGEDLICNLVLTFKEAVFGVEKEIDVSRLDNCTTCDGSGAKSGTKAYTCTTCGGQ
GQVVSSSRTPLGVFQQVMTCSACSGTGETFTPCDKCGGDGRERKSKKISLKVPAGVDSGS
RLRVRSEGNAGKRGGAPGDLFVVIEVIPDPVLKRDDTNILYTCKISYIDAILGTTVKVPT
VDGSADLKIPAGIQPGTTLVMSKKGVPLLNKGRMRGDQLVRVQVEIPRQLSDEERRLVEE
LANLKKTKTLNGSRR
>GbDnaJ41
MVKDTAYYDVLDVNVDASAAEIKKAYYLKARLVHPDKNPGDPKAAENFQALGEAYQVLSD
PEKREAYDKHGKAGVQPDSMLDPSAVFGMLFGSEFFEEYVGQLALASLATVETEIDDDSL
DKDARMQKLQEKMKTVQKEREEKLITLLKNRLEPFVEGQTDEFINWANSEARQLSKAAFG
EAMLHTIGYIYTRKAAKELGKDKRYMKVPFLAEWVRDKGHRIKSQVMAASGAVSLIQIQE
DLKKANQGENREENIMKTFEDKKDAMLQSLWQINVVDIESTLSHVCLAVLKDPSVSKEVL
VLRAKALKKLGAIFQVWPSLPEFSEQ
>GbDnaJ42
MVKDTAYYDVLDVNVDASAAEIKKAYYLKARLVHPDKNPGDPKAAENFQALGEAYQVLSD
PEKREAYDKHGKAGVQPDSMLDPSAVFGMLFGSEFFEEYVGQLALASLATVETEIDDDSL
DKDARMQKLQEKMKTVQKEREEKLITLLKNRLEPFVEGQTDEFINWANSEARQLSKAAFG
EAMLHTIGYIYTRKAAKELGKDKRYMKVPFLAEWVRDKGHRIKSQVMAASGAVSLIQIQE
DLKKANQGENREENIMKTFEDKKDAMLQSLWQINVVDIESTLSHVCLAVLKDPSVSKEVL
VLRAKALKKLGAIFQLLTVERTACAMKTTKR
>GbDnaJ43
MVKDTAYYDVLDVNVDASAAEIKKAYYLKARLVHPDKNPGDPKAAENFQALGEAYQVLSD
PEKREAYDKHGKAGVQPDSMLDPSAVFGMLFGSEFFEEYVGQLALASLATVETEIDDDSL
DKDARMQKLQEKMKTVQKEREEKLITLLKNRLEPFVEGQTDEFINWANSEARQLSKAAFG
EAMLHTIGYIYTRKAAKELGKDKRYMKVPFLAEWVRDKGHRIKSQVMAASGAVSLIQIQE
DLKKANQGENREENIMKTFEDKKDAMLQSLWQINVVDIESTLSHVCLAVSSKRKKKTV
>GbDnaJ44
MFGRVPKKSDNTKFYEVLGVPKSASQDELKKAYKKAAIKNHPDKGGDPEKFKELAHAYEV
LSDPEKRDIYDQYGEDALKEGMGGGGSSHNPFDIFESFFGGGAFGGGGSSRGRRQKRGED
VVHALKVSLEELYNGTAKKLSLSRNAICSKCKGKGSKSGAFSRCYGCQGTGMKITTRQIG
LGMIQQMQHVCPECRGSGEMISDRDRCPQCKGNKVTQEKKVLEVHIEKGMRGGQKITFEG

QADEAPDTITGDIVFVLEQKKHPKFDRKFGDDLYVDHNLSLTEALCGFQFALTHLDGRQL
LIKSNPGEVIKPGQYKAINDEGMPHHQRPFMKGKLVIQFHVIFPESGVFSPEQCRKIESI
LPMRPSKHLTDMELDDCEETTLHDVSAEEVKRRKERQHRHREAYDEDDDDDESAPRVQCA
QQ
>GbDnaJ45
MALPRSKLLFLLFALSLAIVAIAGKSYYDILQVPKGASDEQIKRAYRKLALKYHPDKNPG
NEEANKRFADINNAYEVLSDSEKRGIYDRYGEEGLKQHAASGGRGGMGVNIQDIFSSFFG
GGSVEEEEKIVKGDDVIVELDATLEDLYMGGTLKVWREKNILKPAPGKRRCNCRNEVYHK
QIGPGMFQQMTEQVCEQCQNVKYEREGYFVTVDIEKGMQDGQEVVFYEDGEHIIDGEPGD
LKFRIHTAPHDRFRREGNDLHTTVTITLVQALVGFDKTIKHLDDHLVEIGSKGITKPKEV
RKFKGEGMPLHFSNKKGDLFVTYEVLFPTSLAEDQKAKIKSILG
>GbDnaJ46
MALPRSKLLFQLFALSFAIVAIAGKSYYDILQVPKGASDEQIKRAYRKLALKYHPDKNPG
NEEASKRFADINNAYEVLSDSEKRGIYDRYGEEGLKQHAASGGRGGMGVNIQDIFSSFFG
GGSVEEEERIVKGDDVIVELDATLEDLYMGGTLKVWREKNILKPAPGKRHCNCRNEVYHK
QIGPGMFQQMTEQVCEQCQNVKYEREGYFVTVDIEKGMQDGQEVVFYEDGEPIIDGEPGD
LKFRIHTAPHDRFRREGNDLHTTVTITLVQALVGFDKTIKHLDDHLVEIGSKGITKPKEV
RKFKGEGMPLHFSNKKGDLFVTYEVLFPTSLAEDQKAKIKSILG
>GbDnaJ47
MDREGGSHGGSCYYSVLGIRKDASFSDIRTAYRKLAMKWHPDRYARNPGIAGEAKRRFQQ
IQEAYSVLSDESKRSMYDAGFYDPLEEEDQDFCDFMQEMMSMMNNVKDEGDSFEDLQRMF
AEMVDGISFDINTDPTVTKTARVTPSKGNAAKRNSSWC
>GbDnaJ48
MGLDYYKILKVSSNVNDEDLKKAYKRLAMKWHPDKNPLNNEDAEAKFKQIVEAYEVLSDP
QKRAVYDRYGEEGLKGVAPPPASGGASFFSTGYGIPTTFQFNQQNVADEIFSEFFGAFGG
GLRGTRFSSSLFGNDIFGGGGGGRGGVRFSGSVFGDDIFGSTNQGALRKAAPMENWLPCS
LEELYKGTTQKMKISREIVHISGNIMQVEEILTINVRPGWKKGTKITFAEKGNQRPNVIP
ADLIFIIEEKPHNVFTREGNDLIVAQKISLVDALYGCTVHLTTLDGRNLTIPIHNVIHPC
YEEVVPREGMPIQKDPSKRGNLRIKFDLKFPTRLTPEQKSGIKKLLGP
>GbDnaJ49
MGKRKKSRASRDEDEEDEVVQEEEGNHSSSNEKSLYEILGVEKAASQQEIKKAYYKLALR
LHPDKNPGDEEAKEKFQQLQKVISILGDEEKRAVYDQTGCVDDTDLAGDVVENLKSFFRT
MYKKVTDADIEEFEANYRGSDSEKKDLIDLYRKFKGNMNKLFCSMLCSDPKLDSHRFKDM
LDESIAARELKESKAYKKWAKKVSEMKPPTSSLRRKGKSNKQPESDLFAIISQRRNERKD
RFDSMFSSLVSKYGSNAESEPTEEEFEAAKRKVESKEASNKSKRKRQ
>GbDnaJ50
MGKRKNSGVSRDEEEEVEVENHSSSNEKSLYEVLNVAKTASQQEIKKAYYKLALRLHPDK
NPGDEEAKEKFQQLQKVISILGDEEKRAVYDQTGCVDDADLAGDVVENLKTFFRAMYKKV
TEADIEEFEVNYRGSDSEKKDLFDLYKKCKGNMNKLFCSMLCSDPKLDSHRFKDLLDEAI
AAGELKETKAYRKWANKVSEMKPPTSPLRRKEKSVKQSESDLLAIISQLRNERKDRFDSM
FSTLVSKYGGNADSEPTEEEFEAARRKVESRKASNKSKHK
>GbDnaJ51
MLKSSSLNWAFNSMAWNGLVYTAFILNFVLICQHLLLQPLVSALDGNLGNAAELFERVSQ
SIKVKRYSEALNDLNAAIEADPALSEAYMRHASLLRQLCRYEESEKSYKKFLELKPRNSV

AEKELSQLHQAQSALETAFSLFESKDYTKALDYVDKVVLVFSPACSKAKMLKLKLLVAAK
DYSSVISESGFILKEDENNLEALLLRGHAYYYLADHDVAQRHYQKGLRLDPEHGELKKAY
FRLKNLLKKTKSAEDNANKGKLRIAVEDYKGALALDPNHLAHNVHLHLGLCKVLVRLGRG
KDALSSCSEALNIEEELLEALVQRGEAKLLTEDWEGAVEDLKSAAEKSPQDMNIREALMR
AEKALKLSKRKDWYKILGVSKTASVAEIKRAYKKLALQWHPDKNVDNREEAEAQFREIAA
AYEVLGDEEKRTRYDRGEDIEDIGMGGGGFNPFGGGGGGQHFTFTFDGGFPGGFGGGFPG
GDGGGGGFGFNF
>GbDnaJ52
MGVDYYKILQVDRNAKDEDLKKAYRKLAMKWHPDKNPNNKKDAEAKFKQISEAYDVLSDP
QKRAVYDQYGEEGLKGQMPPPGAGGFGGGDSGGPTTFRFNTRNPDDIFSEFFGFSSPFGM
GDMGGPRAGASGFPRGMFGEDIFGSFRGGAGEGSTTMPRKGAPIDRPLPCSLEDLYKGTT
KKMKISRDVMDGSGRPTTVEEILTIDIKPGWKKGTKITFPEKGNEQRGVIPSDLVFIIDE
KPHSVFKRDGNDLIVTQKISLVEALTGYTAQLTTLDGRNLTVPINNIISPTYEEVVKGEG
MPIPKDPSKKGNLRIKFNIKFPTKLTAEQKTGIKRLIGSP
>GbDnaJ53
MGVDYYKILQVDRNANDEDLKKSYRKLAMKWHPDKNPKSKKEAEAKFKQISEAYDVLSDP
QKRAVYDQYGEEGLKGQMPPPGAGGFPGGADGGSGPTMFRFNPRNPEDIFSEFFGFSSPF
GGMGDMGGSRAGMSGFPRGMFREDIFGSFRGGAGEGSTTMLCKGPAIEQPLPCSLEDLYK
GTTKKMKISRDVTDASGRPSTEEEILTIQIKPGWKKGTKITFPEKGNEQRGVIPSDLVFI
IDEKPHSVFKRDGNDLMLTQKISLVEALTGYTAQLTTLDGRTLTVPINNIINPTYEEVVK
GEGMPIPKEPSKKGNLRIKFNVKFPTKLTTEQKTGLKRLISSP
>GbDnaJ54
MPPHRSKSEKNDGMAKHLRRDPYEVLGVLRNSTDQEIKSAYRKMALKYHPDKNGNDPVAA
DMFKEVTFSYNILSDPDKRRQYDTAGFEAVEADNQELELDLSSLGAVNTIFAALFSKLGV
PIKTTVSATVLEEALNGAVNIRPLQLGQPISRKVEKQCAHFYSVTITEEARDGFVCRVQS
SDKSKFKLLYFELEENGGLSLALQEDSAKTGKVTSAGMYFLGFPVYCLDQTTNSVAAAKD
PDAAFFKKLDGFQPCEITELKPGKHVFAVYGDNFFKSVSYTIEAICAAPFIEEKENLRAV
EAEILSKRAELSKFETEYREVLAQFTEMTGRYTKEMQEIDELLKQRNEIHASYTTIPLIK
RSSSRKKSKAASKEAEEDGEVRDRKPSTRDRTKKKWYNIPLKVDKRKPC
>GbDnaJ55
MPPHRSKSEKNDGMAKHLRRDPYEVLGVLRNSTDQEIKSAYRKMALKYHPDKNGNDPVAA
DMFKEVTFSYNILSDPDKRRQYDTAGFEAVEADNQELELDLSSLGAVNTIFAALFSKLGV
PIKTTVSATVLEEALNGAVNIRPLQLGQPISRKVEKQCAHFYSVTITEEARDGFVCRVQS
SDKSKFKLLYFELEENGGLSLALQEDSAKTGKVTSAGMYFLGFPVYCLDQTTNSVAAAKD
PDAAFFKKLDGFQPCEITELKPGKHVFAVYGDNFFKSVSYTIEAICAAPFIEEKENLRAV
EAEILSKRAELSKFETEYREVSIPLTQCSCLY
>GbDnaJ56
MEKDTSYYDILGVSVDASIPEIKKAYYLNARRVHPDKNLDDPKAADKFHALGEAYQVLTD
PEKREAYDKRGKTGVIPDTMLDPTAVFGMLFGSDFFEDYVGQLAMATLSAIEVESDSLLD
EEAHRKKLEENMQAFQKQREDKLIEILKNRLQPFVEGQTNEFIQWANSEARDLSKAAFGE
AMLHTIGYIYTRKAARELGKDGSCMQVPFLAEWVRDKGHRIKSQVKAAKGAVSLIQIQDE
LRRLNQGADREENILKALEEKKDAMLQSLWQINVVDIESTLSNVCLEVLRDASVSEEVLV
LRAKGMKKLGAIFQGAKSAYSRENSLRRVNVETETAGS
>GbDnaJ57

MFGRAPKKSNNTRYYEILGVSNNASQDDLKKAYKKAAIKNHPDKGGDPEKFKELAQAYEV
LSDPEKREIYDQYGEDALKEGMGGGAGAHDPFDIFSSFFGGSPFGGGSSRGRRQRRGEDV
VHPLKVSLEDLYLGTSKKLSLSRNVICSKCNGKGSKSGASMTCPGCQGSGMKVSIRQLGP
SMIQQMQHPCNECKGTGETINDKDRCPQCKGEKVVQEKKVLEVIVEKGMQNGQKITFPGE
ADEAPETVTGDIVFVLQQKDHPKFKRKGEDLFLEHTLSLTEALCGFQFVITHLDGRQLLI
KSNPGEVVKPDSCKAINDEGMPLYQRPFMKGKLYIQFTVEFPDSLGPDQVKALEAILPPK
PTSQLSDMELDECEETTLYDVNIEEEMRRKQQQAAQEAYEEDEDMHGGAQRVQCAQQ
>GbDnaJ58
MLKTLSLSWAFNSMAWRGLLYTVFILHFVLVCQLLLLQPLVSALDGKPGNAAELFESVSQ
NIKVKRYSEALNDLNAAIETDPALSEAYFHRASILRQLCRYEESEKSYKKFLELKPGNSI
GEKELSQLRQAQSALETAFSLFDSRDHTKGLEYLDKVVLVFSPACSKAKILKAKLLLAAK
YYSSVISETGFILKEDENNLEALLLRGQAYYYLADHDVAQRHYQKGLRLDPEHSELKKAY
FGLKNLLKKTKSAEDNVNKGKLRLAVEDYKGALALDPDHLAHNVHLHLGLCKVLVKLGRG
KDASISCSEALNIDGELLEALVQRGEAKLLTEDWEGAVDDLKSAAQKSPQDMNIREALMR
AEKALKMSKRKDWYKILGVSKTSSVAEIKRAYKKLALQWHPDKNVDNREEAEAQFREIAA
AYEVLGDDEKRAKYDRGEDIEDMGMGGGGFNPFGGGGGQQFTFTFDGGFPGGFGGGGFGF
NF
>GbDnaJ59
MFGRAPKKSNNTRYYEILGVSKNASQDDLKKAYKKAAIKNHPDKGGDPEKFKELAQAYEV
LSDPEKREIYDQYGEDALKEGMGGGAGAHDPFDIFSSFFGGSPFGGGSSRGRRQRRGEDV
VHPLKVSLEDLYLGTSKKLSLSRNVICSKCNGKGSKSGASMTCPGCQGSGMKVSIRQLGP
SMIQQMQHPCNECKGTGETINDKDRCPQCKGEKVVQEKKVLEVIVEKGMQNGQKITFPGE
ADEAPETVTGDIVFVLQQKDHPKFKRKGEDLFLEHTLSLTEALCGFQFVITHLDGRQLLI
KSNPGEVVKPDSCKAINDEGMPLYQRPFMKGKLYIQFTVEFPDSLSPDQVKALEAILPPK
PTSQLSDMELDECEETTLYDVNIEEEMRRKQQQAAQEAYEEDEDMHGGAQRVQCAQQ
>GbDnaJ60
MLKTLSLSWAFNSMAWRGLLYTVFILHFVLVCLQPLVSALGGKPGNAAELFESVSQNIKV
KRYSEALNDLNAAIETDPALSEAYFHRASILRQLCRYEESEKSYKKFLELKPGNSVGEKE
LSQLRQAQSALETAFSLFDSRDHTKALEYLDRVVLVFSPACSKAKILKAKLLLAAKDYSS
VISETGFILKEDENNLEALLLRGQAYYYLADHDVAQRHYQKGLRLDPEHSELKKAYFGLK
NLLKKTKSAEDNVNKGKLRLAVEDYKGALALDPNHLAHNVHLHLGLCKVLVKLGRGKDAS
SSCSEALNIDGELLEALVQRGEAKLLTEDWEGAVDDLKSAAQKSPQDMNIREALMRAEKA
LKMSKRKDWYKILGVSKTSSVAEIKRAYKKLALQWHPDKNVDNREEAEAQFQEIAAAYEV
LGDDEKRAKYDRGEDIEDMGMGGGGFNPFGGGGGGGQQFTFTFDGGFPGGFGGGGFGFNF
>GbDnaJ61
MVGSNGVRLIHCLLRRSHSLSSTLFHHPNSTLISGGSRSFTAGLGNPVNVIGKHTPQYAK
TRNCLVFGPFNVNLGAKRLIHGTAPLSARDYYDTLGVSKNATASEIKKAYFGLAKKLHPD
VNKDDPEAEKKFQEVSKAYEVLKDENKRAEYDQVGHEAFEQQQNNSGFSEEDFNPFKFHN
FHDIFNVQDIFRNQMGGEDIKVAIELSFMEAVQGCSKTVTFQAPVLCQACGGEGVPPGVK
PERCRHCGGSGMLSINKGFMSIRSTCPHCGGSGQFVSKLCKSCNGARLVKGPKTVKLDIM
PGVDNNETLKVYGSGGADPDRTHPGDLYVTIKVRQDPVFRREGANIHVDAVLSVVQAILG
GTIQVPTLTGDVVLKVRPGTQPGQKVVLKNKGIKTRNSYSFGDQYVHFNVSIPKNLTGRQ
RELIEEFAREEQGESDKRAAGAAG
>GbDnaJ62

MALPRTKRFFLLCAFSYAIVAIAEKNYYEILQVPKGASDEQIKRAYRKLALKYHPDKNPG
NEEANKRFADINNAYEVLSDSQKRSIYDRYGEEGLKQHAARAGGGMGVNMHDIFSSFFGG
GPIEEERIVKGDDVTVELEATLEDLYMGSTLKVWREKNILKPAPGKRPCNCRNEVYHRQI
GPGMFQQMTEQVCEQCQNVKFEREGFNVTIDIEKGMQDGQEVVFYEDGEPIVDGEPGDLK
FRIRTAPHDRFRREGDDLHATVTITLVQALVGFEKTIKHLDDHLVNVGSKDITKHKEVRK
FKGEGMPLHSSKKKGDLYVTYDVLFPTSLTEDQKSKIKEVLG
>GbDnaJ63
MDREGGSHGGSCYYTILGIRKDASFSDIRTAYRKLALKWHPDRYVTNPAVAGEAKLRFQQ
IQEAYSVLSNGSKRSMYDASLYDPSADDDQDFCDFMQEMISMMNNVKDEGDSFEDLQKMF
ADMVGSGDFMSFNVNTDPTETKKVHITASKT
>GbDnaJ64
MEGNHNSTPKDYYKILEVEYDATDEKIRLNYRKLALKWHPDKHKGDSSVTAKFQEINEAY
NVLIDLDKRFEYDITGIYEIDKYTLRVRIHVNRMIFWSRIVML
>GbDnaJ65
MEGNHNSTPKDYYKILEVEYDATDEKIRLNYRKLALKWHPDKHKGDSSVTAKFQEINEAY
NVLIDLDKRFEYDITGIYEIDKYTLREYLARFKGMILTCNGLGIPHTSIWTQQLRETNEY
ADDEKG
>GbDnaJ66
MGVDYYNVLKVEKNATDDDLKKSYRKLAMKWHPDKNPNDKKEAETKFKQISEAYEVLSDP
QKRAVYDQYGEEGLKDMPPPGSSRPPFGKGTSGPNGFNPRNAEDIFAEFFGSSPFGFGSS
GPTGSARFHSDGAKFGGFNSTDNIFRSHNEATAPRKPPPVESKLPCSLEELYTGSTRKMK
ISRTVVNAAGRQVQESEILTIDVKPGWKKGTKITFPDKGNEQPNQLPADLVFVIDEKPHD
LYKRDGNDLVVNQRVLLEEALGGTTVNLVTLNGRNLSLPVTDIIGPGYELVIAREGMPIA
KETGNRGDLRIKF
>GbDnaJ67
MGVDYYKILQVDKNAKDDDLKKAYRKLAMKWHPDKNPNNKKEAEAKFKQISEAYEVLSDP
QKRAIYDQYGEEGLKGQVPPQDAGGPGGATFFQTGDGPNVFRFNPRNANDIFAEFFGYSS
PFGGMGGSGMRGSSRSFGGMFGDDIFSSFGEGRPMSQNPRKPPPIENTLPCSLEDLYKGT
TKKMKISREIADASGKTLPVQEILTIDIKPGWKKGTKITFPEKGNEQPNTIPADLVFIID
EKPHSTFTRDGNDLVVTQKISLAEALTGYTVHLTTLDGRSLNIPINSVIHPNYEEVVPKE
GMPIPKDPSKRGNLRIKFNIKFPTRLTAEQKSGIKKLLGPSGGL
>GbDnaJ68
MFGRAPKKSDNTKYYEILGVPKTASQDDLKKAYRKAAIKNHPDKGGDPEKFKELAQAYEV
LSDPEKREIYDQYGEDALKEGMGGGGGAHDPFDIFQSFFGGNPFGGGGSSRGRRQRRGED
VIHPLKVSLEDLYNGTSKKLSLSRNIICSKCKGKGSKSGASMKCSGCQGSGMKVSIRHLG
PSMIQQMQHPCNDCKGTGETINDKDRCPQCKGEKVVQEKKVLEVNVEKGMQNGQKITFPG
EADEAPDTVTGDIVFVLQQKDHPKFKRKGDDLFVEHTLTLTEALCGFQFILTHLDGRQLL
IKTHPGEVVKPDQFKAINDEGMPMYQRPFMRGKLYIHFTVDFPDSLAPEQCKALEAVLPP
RTSVQLTDMELDECEETTLYDVNIEEEMRRKQAQAAQEAYEEDDDMHGGAQRVQCAQQ
>GbDnaJ69
MRSKKMEGTSAPSLRRDPYEVLCVSRDSSDQEIKTAYRKLALKYHPDKNANNPEASELFK
EVAYSYSILSDPEKRRQYDTAGFEAVEDSMDMEIDLSNLGTVNTMFAALFSKLGVPIKTT
ISANVLEEALNGTVTVRPLPIGTSVSGKVDKQCAHFFGVTINDEQAECGIVVRVTSTAQS
KFKLLYFEHDINGGYGLALQEDSEKTGKVTSAGMYFLHFQVYRMDSTVNALAIAKDPESA

FFKRLEGLQPCEVSELKAGTHIFAVYGDNFFKTATYTIEALCAKSYEDTTEKLKDIESQI
LRKRNELRQFETEYRKALARFQEVTNRYTQEKQSVDELLKQRDSIHATFTVTRPPSGISN
LSNGSSSKVPVETESPTEDGNSDGKDKSGKKKWFNLNLMGSDKKLG
>GbDnaJ70
MAIIPCGSTFVAQWHIRPQLTTRSYVPNRIMTARLGVTSTMSYLRASNSGLFARDSLPLL
SFVRPSQTSHHRRGARSIVRAETDYYTVLGVSRNASKSEIKSAYRKLARSYHPDVNKDPG
AETKFKEISNAYEVLSDDEKRSLYDKYGEAGLKGAGMGMGDFSNPFDLFESLFEGMGGMG
GMGMGGRSSRNRAVDGQDEYYSLVLNFKDAVFGVEKEIEITRLESCGTCNGSGAKPGTTP
SKCTTCGGQGQVISSARTPLGVFQQVMTCSSCGGTGEISTPCNTCSGDGRVRRTKRISLK
VPAGVDSGSRLRVRSEGNAGRRGGSAGDLFVVIEVIPDPVLKRDDTNILYTCKVSYIDAI
LGTTIKVLTVDGMVDLKIPAGTQPNTTLVMAKKGVPVLNKTNMRGDQLVRVQVEIPKRLS
SEEKKLIEELADLSKGKTASSRR
>GbDnaJ71
MEGNDNSTSKDYYKILEVDYDATDENIRLSYRKLALKWHPDKHKGDSAVTAKFQEINEAY
NVLIDPDKRFEYDLTGIYEIDKYTLREYLARFKGMILTCNGLGISHTSTW
>GbDnaJ72
MEGNDNSTSKDYYKILEVDYDATDENIRLSYRKLALKWHPDKHKGDSAVTAKFQEINEAY
NVLIDPDKRFEYDLTGIYEIDKYTLREYLARFKGMILTCNGLGISHTSTWTEQLTDRNEF
AEEG
>GbDnaJ73
MVKETEYYDVLGVSPSASEEEIRKAYYLKARQVHPDKNPNDPHAAERFQVLGEAYQILSD
PIQRDAYDRNGKYSITRDTMLDPTAVFALLFGSELFEDYIGHAAVASMASSELANETDNP
EKLNDKLKASAVQKEREEKLAKSLGDFLNQYVRGDKDGFMQHAESEARRLSDTAFGVDIL
HTIGYIYSRQASQELGKKAIYLGVPFLAEWVRHKGHFWKSQITAAKGKNITPGAFQLLQL
QEDMRKQFKMGGSGAENDVEAHLRLNKETLMSSLWKLNVVDIEVTLVHVCQMVLRENNVK
KEELKARALALKILGKTFQEQEARNGGTSRRKNRSRQEHFRIELLFSLRHGIGRLFRCLC
NPAFDVDDDEIVFKSK
>GbDnaJ74
MGVDYYNILKVNRHANEEDLKKAYKRLAMIWHPDKNPSYKRPEAEAKFKLLSEAYDVLSD
PMKRQIYDLYGEEALKSGQFPPPNQSHASTSASYPRGAGHYYNNSNNNQRQQQPNTGPFR
FKPRDADDIYEELFGAEANGGRGNRGFREGHFRNSNGYGTSSTTSNGELRKAAAVENVLH
CSLEELYKGAKKKMRIARNVFDPSVSGKFRTLEEILTIEIKPGWKRSTKITFPEKGNEEP
GVIPADVIFVIEEKPHATYKRDGNDLVVNQEITLLEALTGRTLDLTTLDGRSLVIPLTEI
VKPGAEIVVPNEGMPISKEAGRKGNLRIKLDVKYPSRLTTEQKSELRRVLASVS
>GbDnaJ75
MKEDDGTGPPNRELYALLHLSPEASDEEIRRAYRQWAQVYHPDKYQAPHMKEIATENFQR
ICEAYEILSDENKRQIYDIYGMEGLNSGLELGPKLNKVEEIKEQLEKLKRMKEQQKMSAL
FLPKGSIVANLSLPGFLDGDGIMRGMAMASAVQSQLSKSSALSLSGNLGVEENSGAGAAS
AVFRHQIASDSTIEFMGSVGLGSLIGVQMTRQLSLHSTATLGIAKSFHDGSINLSNVWTR
QLSDTASGNIELLLGPQSSIGVGWQKKDQNTSAAGEVKFGTNSFGISARYSRRFSSKSHG
RIAGRIGSAALEVEVGGGRKVSDFSTVRMLYTIGIRGIFWRFELHRGGQKLLIPILLSRD
LNPVLATGAFVVPTSIYFILKKFVFKPYYLKREKQKALENMERTATQVQEARKAAAKAQQ
LLENVANRKRNKQQETGGLIITKAIYGNHKALKKGDELRETNDELASQVLDVTVPLNFLV
NDTGQLKLHDGVKKSGIMGFCDPCPGEPKQLHMEYTYHGERYEVAVDDYEELIIPQIAHR

V
>GbDnaJ76
MKEDDGTGPPNRELYALLHLSPEASDEEIRRAYRQWAQVYHPDKYQAPHMKEIATENFQR
ICEAYEILSDENKRQIYDIYGMEGLNSGLELGPKLNKVEEIKEQLEKLKRMKEQQKMSAL
FLPKGSIVANLSLPGFLDGDGIMRGMAMASAVQSQLSKSSALSLSGNLGVEENSGAGAAS
AVFRHQIASDSTIEFMGSVGLGSLIGVQMTRQLSLHSTATLGIAKSFHDGSINLSNVWTR
QLSDTASGNIELLLGPQSSIGVGWQKKDQNTSAAGEVKFGTNSFGISARYSRRFSSKSHG
RIAGRIGSAALEVEVGGGRKVSDFSTVRMLYTIGIRGIFWRFELHRGGQKLLIPILLSRD
LNPVLATGAFVVPTSIYFILKKFVFKPYYLKREKQKALENMERTATQIWGYDLQARNVLL
PYISDVTSF
>GbDnaJ77
MATAIPCGNTVVTGLRPRLLSSLNTNKWRISQARVSTEIGALSFSSSSIFPQNLTHLLFN
SWSPKIPYHHLARRLIVKAARDYYSILGVSKNGSKSDIKSAYRKLARNYHPDVNKEAGAE
QKFKEISEAYEVLSDDEKRSIYDRYGKDGFKGSTMDMGDFSNPFDLFSSLFDMDIRNRGA
RNMAADGEDLICNLVLTFKEAVFGVEKEIDVSRLDNCTTCDGSGAKPGTKAYTCTTCCGQ
GQVVSSSRTPLGVFQQVMTCSACSGTGETFTPCNKCGGDGRERKSKKISLKVPAGVDSGS
RLRVRSEGNAGKRGGAPGDLFVVIEVIPDPVLKRDDTNILYTCKISYIDAILGTTVKVPT
VDGSADLKIPAGVQPGTTLVMSKKGVPLLNKGKMRGDQLVRVQVEIPRQLSDEERRLVEE
LANLKKTKTLNGSRR
>GbDnaJ78
MVKDTAYYDVLGVNVDASAAEIKKAYYLKARLVHPDKNPGDPKAAENFQALGEAYQVLSD
PEKREAYDKHGKAGVQPDSMLDPSAVFGMLFGSEFFEEYVGQLALASLATVETEIDDDSL
DKDARMQKLQEKMKTVQKEREEKLITLLKNRLEPFVEGQTDEFINWANSEARRLSKADFG
EAMLHTIGYIYTRKAAKELGKDKHYMKVPFLAEWVRDKGHRIKSQVMAASGAVSLIQIQE
DLKKANQGENREENIMKTLEDKKDAMLQSLWQINVVDIESTLSHVCLAVLKDPSVSKEVL
VLRAKALKKLGAIFQGAKAAHSRENSLRRENDKAIKASREWLSSLCG
>GbDnaJ79
MMAIIHYGSTCVAQCGIRSLFTIRSYVPNRLITAQSGVKSTSYLGAPSSSLFSRDSFPLL
SYMGSSQTSHCRRGARFIVRAETDYYTVLGVSRNASKSEIKSAYRKLARSYHPDVNKDPG
AEQKFKEISNAYEVLSDDEKRSLYDKYGEAGLKGAGMGMGDFSNPFDLFESLFEGMGGMG
GMGMGGSSSRNRAVDGQDKYYSLVLNFKEAVFGVEKEIEITRLESCGTCNGSGAKPGTTA
SKCTTCGGQGQVISSARTPLGVFQQVMTCSSCGGMGEISTPCNTCSGDGRVRKTKRISLK
VPAGVDSGSRLRVRSEGNAGRRGGSPGDLFVVIQVILDPVLKRDDTNILYTCKVSYMDAI
LGMTIKVPTVDGMVDLKIPAGTQPNTTLVMAKRGVPVLNKSNMRGDQLVCVQVEIPKQLS
SEERKLIEELSDLSKGKTASSRR
>GbDnaJ80
MGVDYYKILQVDRNANDEDLKKAYRKLAMKWHPDKNPKSKKEAEAKFKQISEAYDVLSDP
QKRAVYDQYGEEGLKGQMPPPGAGGFPGGADGGSGPTMFRFNPRSPEDIFSEFFGSSSPF
GGMGDMGGSRAGMSGFPRGMFRGDIFGSFRGGAGEGSTTMLRKGPAIEQPLPCSLEDLYK
GTTKKMKISRDVIDASGRPSTEEEILTIQIKPGWKKGTKITFPEKGNEQRGVIPSDLVFI
IDEKPHSVFKRDGNDLILTQKISLVEALTGYTAQLTALDGRTLTVPINNIINPTYEEVVK
GEGMPIPKEPSKKGNLRIKFNIKFPTKLTTEQKTGLKRLISSP
>GbDnaJ81
MPPHRSKSEKNDGIAKHLRRDPYEVLGVLRNSTDQEIKSAYRKMALKYHPDKNGNDPVAA

DMFKEVTFSYNILSDPDKRRQYDTAGFEAVEADNQELELDLSSLGAVNTMFAALFSKLGV
PIKTTVSATVLEEALNGAVIVRPLQLGQPISRKVEKQCAHFYSVTITEDEARDGFVCRVQ
SSDKSKFKLLYFELEENGGLSLALQEDSAKTGKVTSAGMYFLGFPVYCLDQTTNSVAAAK
DPDAAFFKKLDGFQPCEITELKPGKHVFAVYGDNFFKSVSYTIEAICAAPFIEEKENLRA
VEAEILSKRAELSKFETEYREVSIPLTQCSCLY
>GbDnaJ82
MPPHRSKSEKNDGIAKHLRRDPYEVLGVLRNSTDQEIKSAYRKMALKYHPDKNGNDPVAA
DMFKEVTFSYNILSDPDKRRQYDTAGFEAVEADNQELELDLSSLGAVNTMFAALFSKLGV
PIKTTVSATVLEEALNGAVIVRPLQLGQPISRKVEKQCAHFYSVTITEDEARDGFVCRVQ
SSDKSKFKLLYFELEENGGLSLALQEDSAKTGKVTSAGMYFLGFPVYCLDQTTNSVAAAK
DPDAAFFKKLDGFQPCEITELKPGKHVFAVYGDNFFKSVSYTIEAICAAPFIEEKENLRA
VEAEILSKRAELSKFETEYREVLAQFTEMTGRYTKEMQEIDELLKQRNEIHASYTTIPLI
KRSSSRKKSRAASKEAKEDGEVRDRKPSTRDRTKKKWYNIPLKVDKRKPC
>GbDnaJ83
MEKDTSYYDILGVNVDASIPQIKKAYYLKARRVHPDKNPDDPKAADKFHALGEAYQVLTD
PEKREAYDKRGKTTVIPDTMLDPTAVFGMLFGSDFFEDYVGQLAMATLSAIEVESNSLLD
KEAHRKKLEENMQAFQKQREDKLIEILKNRLQPFVEGHTNEFIQWANSEARDLSKAAFGE
AMLHTIGYIYTRKAARELGKDGSCMHVPFLAEWVRDKGHHIKSQVKAASGAVSLIQIQDE
LRRLTQGADREENILKALEEKKDAMLQSLWQINVVDIESTLSNVCLEVLRDASVSEEVLV
LRAKGMKKLGTIFQGAKSAYSRENSLRRVNVETETAGS
>GbDnaJ84
MAPPGTIRWCALVLTLFLSLYISPSIAIYCDEDDCYDLLGVSQSANASEIKKAYYKLSLK
YHPDKNPDPESRKLFVKIANAYEILKDEATREQYDYAIAHPEEVFYNTARYYRAYYGHKT
DTRAVLVGVLLILSVFQYFNRLTRYNQAVDMVKKTPAYKNRLRALELERSGGTTNKKKSN
RQIIKKKEEDLSNELELDIKGAEKPSIWELIGVRFILLPYTIGKLLLWYGCWFWRYKVKQ
APYSWEDAAYLTRNSLRVPLDAWLNIDESTKEDLLQRRLWIKSNLDSYLSEMRKEHKRRR
>GbDnaJ85
MGRFNWLRLCRRHWVSTMPVEWTIDGGDGFRKFSTIFQSLNRERALHSCSFVTGKPLDLM
TPGMSLKERYFHSTGSHFSAKQDYYEILGVPENATRDEIKKAYHALAKKYHPDANKNNPS
AKRKFQEITDAYETLQNPEKRREYDTMRAGSSEDMGYGANGAEGFGFYGASGAKGFRYTY
QTNFSDSFSKIFSEIFQDEMDQFAPDIQTEILLSFSEAAKGCTKDLQFDAFVTCDSCDGR
GYPPNAKVKICSICRGSGTITIPPFTSTCHACKGSGRIIKEYCMSCQGSGVVEGVKEIKV
TIPAGVDSGDTIRVPEAGNIRRQGSQLGNLFIKIKVAADPVFTRDGADVYVDSNISFTQA
ILGGAVEVPTLSGKIQVKIPKGCQHGHLLALRGKGLPKHGFLAYHGDQYVRFRVNLPIEI
NNRQRAILEELAKEEINNENESTDEGN
>GbDnaJ86
MGRFNWLRLCRRHWVSTMPVEWTIDGGDGFRKFSTIFQSLNRERALHSCSFVTGKPLDLM
TPGMSLKERYFHSTGSHFSAKQDYYEILGVPENATRDEIKKAYHALAKKYHPDANKNNPS
AKRKFQEITDAYETLQNPEKRREYDTMRAGSSEDMGYGANGAEGFGFYGASGAKGFRYTY
QTNFSDSFSKIFSEIFQDEMDQFAPDIQTEILLSFSEAAKGCTKDLQFDAFVTCDSCDGR
GYPPNAKVKICSICRGSGTITIPPFTSTCHACKGSGRIIKEYCMSCQGSGVVEGVKEIKV
TIPAGVDSGDTIRVPEAGNIRRQGSQLGNLFIKIKVAADPVFTRDGADVYVDSNISFTQA
ILGGAVEVPTLSGKIQVKIPKGCQHGHLLALRGKGLPKHGFLAYHGDQYVRFRVNLPIEI
NNRQRAILEELAKEEINNENESTDEGNW

>GbDnaJ87
MGRFNWLRLCRRHWVSTMPVEWTIDGGDGFRKFSTIFQSLNRERALHSCSFVTGKPLDLM
TPGMSLKERYFHSTGSHFSAKQDYYEILGVPENATRDEIKKAYHALAKKYHPDANKNNPS
AKRKFQEITDAYETLQNPEKRREYDTMRAGSSEDMGYGANGAEGFGFYGASGAKGFRYTY
QTNFSDSFSKIFSEIFQDEMDQFAPDIQTEILLSFSEAAKGCTKDLQFDAFVTCDSCDGR
GYPPNAKVKICSICRGSGTITIPPFTSTCHACKGSGRIIKEYCMSCQGSGVVEGVKEIKV
TIPAGVDSGDTIRVPEAGNIRRQGSQLGNLFIKIKVAADPVFTRDGADVYVDSNISFTQA
ILGGAVEVPTLSGKIQVKIPKGCQHGHLLALRGKGLPKHGFLAYHGDQYVRFRVNLPIEI
NNRQRAILEELAKEEINNENESTDEGNWLYQQLSTG
>GbDnaJ88
MAATTSLSLLPSSLGFPNERPSSTSQSSSYSCSCSVFFNAGTRLRSHDSFACVTFPSSSS
TCYWRFNNRAGTHRFGTTVVAASGDYYATLGVPKSASGKEIKAAYRRLARQYHPDVNKEP
GATEKFKEISAAYEVLSDDKKRALYDQYGEAGVKSAVGGQSSAYTTNPFDLFETFFGPSM
GGFPGMDQTGFGTSRRSTVSKGDDIRYDITLEFSEAIFGAEKEFELSHLETCEVCLGTGA
KVGSKMRICSTCGGRGQVMRTEQTPFGLFSQVSVCPNCGGDGEVISENCRKCSGKGRIRV
KKNIKVKVPPGVSAGSILRVAGEGDAGPKGGPPGDLYAYLDVQEVPGIQRDGINLLSTVS
ISYLDAILGSVVKVKTVEGVTDLQIPPGTQPGDVLVLARKGAPKLNKPSIRGDHLFTIKV
DIPNRISAKERELLEELSSLSNTNGSRSRTRPRTQPATATKTSGSKVSTDGEKTEEAAAD
ENDPWTKLKKFAGSIANGAAKWLKDNL
>GbDnaJ89
MAATTSLSLLPSSLGFPNERPSSTSQSSSYSCSCSVFFNAGTRLRSHDSFACVTFPSSSS
TCYWRFNNRAGTHRFGTTVVAASGDYYATLGVPKSASGKEIKAAYRRLARQYHPDVNKEP
GATEKFKEISAAYEVLSDDKKRALYDQYGEAGVKSAVGGQSSAYTTNPFDLFETFFGPSM
GGFPGMDQTGFGTSRRSTVSKGDDIRYDITLEFSEAIFGAEKEFELSHLETCEVCLGTGA
KVGSKMRICSTCGGRGQVMRTEQTPFGLFSQVSVCPNCGGDGEVISENCRKCSGKGRIRV
KKNIKVKVPPGVSAGSILRVAGEGDAGPKGGPPGDLYAYLDVQEVPGIQRDGINLLSTVS
ISYLDAILGSVVKVKTVEGVTDLQIPPGTQPGDVLVLARKGAPKLNKPSIRGDHLFTIKV
DIPNRISAKERELLEELSSLSNTNGSRSRTRPRTQPATATFTYSCSQN
>GbDnaJ90
MFGRAPKKSDNTKYYEILGVSKNASQDDLKKAYRKAAIKNHPDKGGDPEKFKELAQAYEV
LSDPEKREIYDQYGEDALKEGMGSGGGGHDPFDIFQSFFGGNPFGAGGSSRGRRQRRGED
VIHPLKVSLEDLYNGTSKKLSLSRNVICSKCKGKGSKSGASMKCTGCQGSGMKVSIRHLG
PSMIQQMQHPCNECKGTGETINDKDRCPQCKGDKVVQEKKVLEVIVEKGMQNGQRITFPG
EADEAPDTVTGDIVFVLQQKDHPKFKRKGDDLFVEHTLALTEALCGFQFILTHLDGRQLL
IKTQPGEVVKPGQFKAINDEGMPIYQRPFMRGKLYIQFTVDFPDSLTPDQCKALEAVLPP
KASVQLTDMELDECEETTLHDVNIEEVMRRKQTQAQQEAYDEDEDMHGGAQRVQCAQQ
>GbDnaJ91
MVKETDFYDILGVSPSATESEIKKAYYMKARQVHPDKNPNDPQAAQNFQVLGEAYQVLSD
PAQRQAYDAHGKAGISTEAIIDPAAIFAMLFGSELFEEYIGQLAMASMASLDIFTEGEQF
DAKKVQEKMKVVQKEREDKLAQLLKDRLNQYVQGNKADFVNHAEAEVSRLSSAAYGVDML
NTIGYIYARQAAKELGKKAIYLGVPFVAEWFRNKGHFIKSQVTAATGAIALLQLQEDMKK
QLSAEGNYSEEELEEYMQSHKKLMIDSLWKLNVADIEATLSRVCQMVLQDQNCKKEELRA
RAKGLKTLGKIFQSVKSTNGNESDPVLGNNARHKLDGVEPSYNSGSPNVSTKSSTRDELS
PSPLAPQSPYVEAPNFVNAQLPRPTAPPGAQRHP

>GbDnaJ92
MVKETEYYDVLGVSPSATEAEIKKAYYIKARQVHPDKNPNDPLAAQNFQVLGEAYQVLSD
PTQRQAYDAYGKSGISAEAIIDPAAIFAMLFGSELFEEYIGQLAMASMASLDIFTEGEQV
DPKKLQEKMKVVQKEREEKLAQILKDRLNQYVQGSKENFVNHAEAEVSRLSNAAYGVDML
NTIGYIYARQAAKELGKKAIYLGVPFIAEWFRDKGHYIKSQVTATTGAIALLQLQEEMKK
QLSAEGNYTEEELEEYMHSHKKILTDSLWKLNVADIEATLSRVCQMVLQDGNCKREELRA
RAKGLKTLGRIFQRAKSANGSESETVESSNTVHTLDGSEPSYDSSSLNASSRSLNQEELS
RSTFASQSPYVEAPNFVDTQFTYNFPRPTAPPGAQRTSLN
>GbDnaJ93
MVKETEYYDVLGVSPSATEAEIKKAYYIKARQVHPDKNPNDPLAAQNFQVLGEAYQVLSD
PTQRQAYDAYGKSGISAEAIIDPAAIFAMLFGSELFEEYIGQLAMASMASLDIFTEGEQV
DPKKLQEKMKVVQKEREEKLAQILKDRLNQYVQGSKENFVNHAEAEVSRLSNAAYGVDML
NTIGYIYARQAAKELGKKAIYLGVPFIAEWFRDKGHYIKSQVTATTGAIALLQLQEEMKK
QLSAEGNYTEEELEEYMHSHKKILTDSLWKLNVADIEATLSRVCQMVVSLVSSFTAVR
>GbDnaJ94
MTSDTSYYDILGVKVSASAAEIKKAYYLKARQVHPDKNPGDPKADEKFAALSEAYQVLSD
PDKREDYDKNGKDGIIPGSMLDPSAVFGMAFGSDYFDEYVGTLAMATLSSLEVEFEESLV
DKEARTQKIREKMEVLQKEREDKLIVTLKNRLQPFLDGQTDEFIYWANGEARRLSKAAFG
EAMLHTIGYIYVRKGASELGKDKRYMKVPFIAEWVRDKGHRVKSQVMAASGAVNLIQIQE
ELKKVNQGEKKDENLMKALEDKREAMLQSLWKVNVVDIETTLSRVCLAVLQDPDASKDVL
ILRAQALKKLGSIFQVCSSEESCNSA
>GbDnaJ95
MTSDTSYYDILGVKVSASAAEIKKAYYLKARQVHPDKNPGDPKADEKFAALSEAYQVLSD
PDKREDYDKNGKDGIIPGSMLDPSAVFGMAFGSDYFDEYVGTLAMATLSSLEVEFEESLV
DKEARTQKIREKMEVLQKEREDKLIVTLKNRLQPFLDGQTDEFIYWANGEARRLSKAAFG
EAMLHTIGYIYVRKGASELGKDKRYMKVPFIAEWVRDKGHRVKSQVMAASGAVNLIQIQE
ELKKVNQGEKKDENLMKALEDKREAMLQSLWKVNVVDIETTLSRVCLAVLQDPDASKDVL
ILRAQALKKLGSIFQGVKVRYSREDSLRHEIDC
>GbDnaJ96
MTSDTSYYDILGVKVSASAAEIKKAYYLKARQVHPDKNPGDPKADEKFAALSEAYQVLSD
PDKREDYDKNGKDGIIPGSMLDPSAVFGMAFGSDYFDEYVGTLAMATLSSLEVEFEESLV
DKEARTQKIREKMEVLQKEREDKLIVTLKNRLQPFLDGQTDEFIYWANGEARRLSKAAFG
EAMLHTIGYIYVRKGASELGKDKRYMKVPFIAEWVRDKGHRVKSQVMAASGAVNLIQIQE
ELKKVNQGEKKDENLMKALEDKREAMLQSLWKVNVVDIETTLSRVCLATLMHPRMF
>GbDnaJ97
MDGNKDDALKCLKIGKDALDAGDRARALKFLTKARRLDPSLHIENLLSAAEGGKSDDRPA
SEPVGSAKDPSGSSPSKSSDQPSIRRRNIPNGSAASASASSPASAAGGTYTEEQIVIVKQ
IRKKKDYYEILGLEKSCSVDDIRKAYRKLSLKVHPDKNKAPGAEEAFKAVSKAFQCLSNE
ESRKKYDLVGSDEPVYERRASPFRGGGNGFSGFYDTDFDADEIFRNFFFGGMPPATTQFR
SFNFGPGMGARMGDQGSTGFNIRMLIQLLPVLLILLFSFLPSSEPVFSLSRSYPYEYKFT
TKNGVNYYVRSTKFEQDYPTNSVERVRIEERVERDYYSVLAQNCRFELQRQQWGFIRETP
HCDLLEKFQSAAAAA
>GbDnaJ98
MKEDDGTGPPNRELYALLHLSPEASDEEIRRAYRQWAQVYHPDKYQAPHMKEIATENFQR

ICEAYEILSDENKRQIYDIYGMEGLNSGLELGPKLNKVEEIKEQLEKLKRMKEQQKMSAL
FLPKGSIVANLSLPEFLDGDGIMRGMAMSSAVQSQLSKSSALSLSGNLGVEENSGAGAAS
AVFRHQIASGSTIEFMGSVGLGSLIGVQMTRQLSLHSTATLGIAKSFHDGSINLSNVWTR
QLSDTASGNIELLLGPQSSIGVGWQKKDQNTSAAGEVKFGTNSFGISARYTHRFSSKSHG
RIAGRIGSAALEVEVGGGRKVSDFSTVRMLYTIGIRGIFWRFELHRGGQKLLIPILLSRD
LNPVLATGALVVPTSIYFILKKFVFKPYYLKREKQKALENMERTATQVQEARKAAAKAQQ
LLENVANRKRNKQQEIGGLVITKAIYGNHKALKKGDELRETNDELASQVLDVTLPLNFLV
NDSGQLKLHDGVKKSGIMGFCDPCPGEPKQLHVEYTYHGERYEVAVDDYEELIIPQIAHR
V
>GbDnaJ99
MGVDYYNILKVNRHANEEDLKKAYKRLAMIWHPDKNPSYKRPEAEAKFKLLSEAYDVLSD
PMKRQIYDLYGEEALKSGQFPPPNQSHASTSASYPRGAGHYYNNSNNNQRQQQPNTDSFR
FKPRDADDIYEELFGAEANGGRGNRGFREGHFRNSNGYGTSSTTSNGELRKAAAVENVLH
CSLEELYKGAKKKMRIARNVFDPSVSGKFRTLEEILTIEIKPGWKRGTKITFPEKGNEEP
GVIPADVIFVIEEKPHATYKRDGNDLVVNQEITLLEALTGRTLDLTTLDGRSLMIPLTEI
VKPGAEIVVPNEGMPISKEAGRKGNLRIKLDVKYPSRLTTEQKSELRRVLASVS
>GbDnaJ100
MPPHRSKSEKNDGMAKHLCRDPYEVLGVSRNSTDQEIKSAYRKMALKYHPDKNGNDPVAA
DMFKEVTFSYNILSDPDKRNQYDTAGFEAVEAENQELELDLSSLGAVNTMFAALFSKLGV
PIKTTVSATVLEEALNGVVTIQPLLLGQPVSRKVEKQCAHFYSVEITEEEARDGFVCRVQ
SSDKSKFKLLYFDQEENGGLSLALQEDSAKTGKVTSAGMYFLGFPVYRLDQTVNSVAAAK
DPDTAFFKKLDGFQPCEITELKPGTHFFAVYGDNFFKSVSYTIEAICTAPFIEEKENLRA
VEAEILSKRVELSKFETEYREVLAQFTEMTTRYTKEMQEIDELLKQRNEIHASYTMIPPS
KRSSSRNRSKGVSREAKEGEVRDRKHSTRDRTKKKRWYNIHLKIDKRKQPC
>GbDnaJ101
MGVDYYNILKVSRNATDDDLRKSYKRLARKWHPDKNLVNNKEAEAKCKQIFEAYNVLSDP
LKRQIYDLHGEQGLNSAESSSPNGFGAGGVGGMANKFDQRNGQGYKKASPVETQLLCSLE
ELYKGGRRRMRISRSIPGEFGKLKTVEEILKIDIKPGWKKGTKITFPEKGNQEPGFTPSD
LIFVVDEKPHAIFKRDGNDLIATLKISLLEALTGTILSLTTLDGRTLPISVTDIVNPGHE
VVIPNEGMPISKEPSKRGHLKIQFDIIFPSKLSAEQKCDLRRALSQR
>GbDnaJ102
MVGSNGVRLIHCLLRRSHSLSSTLFHHPNSTLISGGSRSFTAGLGNPVNVIGKHTPLYAK
TRNCLVFGLFNVNLGAKRLIHGSAPLSARDYYDTLGISKNATASEIKKAYFGLAKKLHPD
VNKDDPEAEKKFQEVSKAYEVLKDENKRAEYDQVGHEAFEQQQNNSGFSEEDFNPFKFHN
FHDIFNVQDIFRNQMGGEDIKVAIELSFMEAVQGCSKTVTFQAPVLCQACGGEGVPPGVK
PERCRHCGGSGMLSINKGFMSIRSTCPHCGGSGQFVSKLCKSCNGARLVKGPKTVKLDIM
PGVDNNETLKVYGSGGADPDRTHPGDLYVTIKVRQDPVFRREGANIHVDAVLSVVQAILG
GTIQVPTLTGDVVLKVRPGTQPGQKVVLKNKGIKTRNSYSFGDQYVHFNVSIPKNLTGRQ
RELIEEFAREEQGESDKRAAGAAG
>GbDnaJ103
MALPRTKRFFLLCAFSYAIVAIAEKNYYEILQVPKGASDELIKRAYRKLALKYHPDKNPG
NEEANKRFADINNAYEVLSDSEKRSIYDRYGEEGLKQHAARAGGGMGVNMQDIFSSFFGG
GPMEEEERIVKGDDVIVELEATLEDLYMGSTLKVWREKNILKPAPGKRPCKCRNEVYHRQ
IGPGMFQQMTEQVCEQCQNVKFGREGFNVTIDIEKGMQDGQEVVFYEDGEPIVDGEPGDL

KFRIRTAPHDRFRREGDDLHATVTITLVQALVGFEKTIKHLDDHLVNVSSKDITKHKEVR
KFKGEGMPLHSSKKKGDLYVAYDVLFPTSLTEDQKSKIKVLG
>GbDnaJ104
MALPRTKRFFLLCAFSYAIVAIAEKNYYEILQVPKGASDELIKRAYRKLALKYHPDKNPG
NEEANKRFADINNAYEVLSDSEKRSIYDRYGEEGLKQHAARAGGGMGVNMQDIFSSFFGG
GPMEEEERIVKGDDVIVELEATLEDLYMGSTLKVWREKNILKPAPGKRPCKCRNEVYHRQ
IGPGMFQQMTEQVCEQCQNVKFGREGFNVTIDIEKGMQDGQEVVFYEDGEPIVDGEPGDL
KVSMLLDAIVTILCSLSLVYWNFLINAHMFA
>GbDnaJ105
MDREGGSHGGSCYYTVLGIRKDASFSDIRAAYRKLALKWHPDRYVTNPAVAGEAKLRFQQ
IQEAYSVLSNGSKRSMYDASLYDPLEDDDQDFCDFMQEMISMMNNVKDEGVSLEDLQKVF
ADMVGSGDGMSFNVNTDLTETKKAHFTASKT
>GbDnaJ106
MEGNHNSTPKDYYKILEVEYDATDEKIRLNYRKLALKWHPDKHKGDSSVTAKFQEINEAY
NVLIDPDKRFEYDITGIYEIDKYTLREYLARFKGMILTCNGLGIPHTSIWTQQLRETNEY
ADDEKG
>GbDnaJ107
MFGRAPKKSDNTRYYEILGVSKNASHDDLKKAYKKAAIKNHPDKGGDPEKFKELAQAYEV
LSDPEKREIYDQYGEDALKEGMGGGAAAHDPFDIFSSFFGGSPFGGASSRGRRQRRGEDV
VHPLKVSLEDLYLGTSKKLSLSRNVICSKCSGKGSKSGASMQCPGCQGSGMKISVRHLGP
SMIQQMQHPCNECKGTGETINDKDRCPQCKGEKVVQEKKVLEVIVEKGMQNGQKITFPGE
ADEAPDTITGDIVFVLQQKDHPKFKRKGEDLFVEHTLALTEALCGFQFVVTHLDGRQLLI
KSIPGEVVKPDSFKAINDEGMPLYQRSFIKGKMYIHFTVEFPDSLNPDQIKALEAILPPK
PTSHLTDMELDECEETTLHDVNIEEEMRRKQQVAQEAYDEDEDTHGRAQRVQCAQQ
>GbDnaJ108
MAPPATIRWCALVLTLFLSLYISPSIAIYCDEDDCYDLLGVSQSANASEIKKAYYKLSLK
YHPDKNPDPESRKLFVKIANAYEILKDEATREQYDYAIAHPEEVFYNTARYYRAYYGHKT
DTRAVLVGVLLILSVFQYFNQLTRYNQAVDMVKKTPAYKNRLRALELERSGGTTNKKKSN
RQIVKKKEEDLSNELELDIKGAEKPSIWELVGVRFILLPYTIGKLLLWYGCWFWRYKVKQ
APYSWEDAAYLTRNSLRVPLDAWLNIDESTKEDLLQRRLWIKSNLDSYLSEMRKEHKRRR
>GbDnaJ109
MFGRAPKKSDNTRYYEILGVSKNASHDDLKKAYKKAAIKNHPDKGGDPEKFKELAQAYEV
LSDPEKREIYDQYGEDALKEGMGGGAAAHDPFDIFSSFFGGSPFGGASSRGRRQRRGEDV
VHPLKVSLEDLYLGTSKKLSLSRNVICSKCSGKGSKSGASMQCPGCQGSGMKISVRHLGP
SMIQQMQHPCNECKGTGETINDKDRCPQCKGEKVVQEKKVLEVIVEKGMQNGQKITFPGE
ADEAPDTITGDIVFVLQQKDHPKFKRKGEDLFVEHTLALTEALCGFQFVVTHLDGRQLLI
KSIPGEVVKPDSFKAINDEGMPLYQRPFIKGKMYIHFTVEFPDSLNPDQIMALEAILPPK
PTSHLTDMELDECEETTLHDVNIEEEMRRKQQAAQEAYDEDEDTHGGAQRVQCAQQ
