# Supplementary material for: Genome-wide identification and expression profiling of DnaJ gene family in Gossypium barbadense reveals candidate thermotolerance genes
Source: Front Plant Sci. 2026 Jan 20;16:1728216. doi: 10.3389/fpls.2025.1728216 (PMC12865410; doi:10.3389/fpls.2025.1728216)
Supplement: Supplementary Data Sheet 1 — Protein sequences of the GbdnaJ gene family. [file Supplementaryfile1.zip › Supplementary Material/Data Sheet 2.PDF]

````
## 1. Reading data
```{r}
##Read histogram data table
library(readr)
rename_plantCARE_BarPlot <- read_csv("rename_plantCARE_BarPlot.csv")
View(rename_plantCARE_BarPlot)
##Read heatmap data table
library(readr)
plantCARE_HeatmapPlot <- read_csv("plantCARE_HeatmapPlot.csv")
View(plantCARE_HeatmapPlot)
```

2.Drawaing
```{r}
library(tidyverse)
library(patchwork)    ## used to combine graphics

# 2.1 Create a bar chart
rename_plantCARE_BarPlot$id <- factor(rename_plantCARE_BarPlot$id,
                                                         levels =
unique(rename_plantCARE_BarPlot$id)[order(as.numeric(gsub("\\D", "",
unique(rename_plantCARE_BarPlot$id))))])

# Add Description legend
bar_plot <- ggplot(rename_plantCARE_BarPlot, aes(x = id, y = Description_num, fill
= Description)) +
   geom_bar(stat = "identity", position = "stack", width = 0.8, color = "black", size =
0.2) +
   scale_fill_manual(values = c(
      "Plant growth and development" = "#A7D4D8",
      "Light response" = "#C7DB95",
      "Plant hormone related" = "#94D09F",
      "Stress related" = "#BCB9D8"
   )) +
   coord_flip() +    # Convert to horizontal bar chart
   scale_x_discrete(limits = rev(levels(rename_plantCARE_BarPlot$id))) +    #
Reverse order matching effect diagram

   theme_minimal() +
   theme(
      axis.text.y = element_text(size = 8, face = "bold"),    # Bold Y axis label (gene
name)
      axis.text.x = element_text(size = 10),
      axis.title.x = element_text(size = 12),
````
````

```r
    legend.position = "left",    # Show legend on the left
    plot.margin = margin(5, 2, 5, 5, "mm"),
    panel.grid.minor = element_blank()
  ) +
  labs(x = "", y = "Number of cis-acting elements", fill = "Description")    # Add
legend title

# 2.2 Create heat map data
# Create a complete gene × category combination
all_genes <- unique(plantCARE_HeatmapPlot$id)
all_categories <- unique(plantCARE_HeatmapPlot$category)
all_descriptions <- plantCARE_HeatmapPlot %>%
  select(category, Description) %>%
  distinct()

# Create a complete data frame
complete_data <- expand.grid(
  id = all_genes,
  category = all_categories,
  stringsAsFactors = FALSE
) %>%
  left_join(all_descriptions, by = "category") %>%
  left_join(plantCARE_HeatmapPlot %>% select(id, category, category_num),
            by = c("id", "category")) %>%
  mutate(
    category_num = ifelse(is.na(category_num), 0, category_num)
  )

# Create heat map quantity grouping
heatmap_data <- complete_data %>%
  mutate(count_group = case_when(
    category_num <= 1 ~ "[0,1]",
    category_num <= 4 ~ "[2,4]",
    category_num <= 10 ~ "[5,10]",
    category_num <= 20 ~ "[11,20]",
     category_num <= 40 ~ "[21-40]",
     category_num <= 60 ~ "[41-60]",
     category_num <= 80 ~ "[61-80]",
     category_num <= 100 ~ "[81-100]",
     category_num <= 120 ~ "[101-120]",
     TRUE ~ "[121-140]"
  )) %>%
  mutate(count_group = factor(count_group, levels = c(
    "[0,1]", "[2,4]", "[5,10]", "[11,20]", "[21-40]",
```

```
    "[41-60]", "[61-80]", "[81-100]", "[101-120]", "[121-140]"
  )))

# Make sure that the gene sequence is consistent with the bar chart
heatmap_data$id <- factor(heatmap_data$id, levels =
rev(levels(rename_plantCARE_BarPlot$id)))

# Adjust spacing and bold fonts
heatmap_plot <- ggplot(heatmap_data, aes(x = category, y = id, fill = count_group)) +
  geom_tile(color = "black", size = 0.2, height = 1.0, width = 0.7) +   # Add
width=0.9 to make spaces between grids.
# Add a digital label
  geom_text(aes(label = category_num), size = 2.5, color = "black") +
  scale_fill_manual(values = c(
    "[0,1]" = "#8CD0C3",
    "[2,4]" = "#FAF5B5",
    "[5,10]" = "#BCB9D8",
    "[11,20]" = "#80B1D2",
    "[21-40]" = "#F18072",
    "[41-60]" = "#F9B063",
    "[61-80]" = "#B3D46B",
    "[81-100]" = "#F7CBDF",
    "[101-120]" = "#D7D7B5",
    "[121-140]" = "#BA7FB5"
  ), name = "Count") +
  facet_grid(~ Description, scales = "free_x", space = "free_x") +
  scale_x_discrete(position = "top") +
  theme_minimal() +
  theme(
    axis.text.x = element_text(angle = 90, hjust = 0, vjust = 0.5, size = 6, face =
  "bold"), #category name bold
    axis.text.y = element_blank(),
    axis.title = element_blank(),
    legend.position = "right",
    plot.margin = margin(1, 1, 1.5, 0, "cm"),
    panel.grid = element_blank(),
    strip.placement = "outside",
    strip.text = element_text(angle = 0, hjust = 0.5, size = 10, face = "bold", margin
= margin(b = 10)),
    panel.spacing = unit(0.5, "lines")
  )

# 2.3 Create a combination diagram
combined_plot <- bar_plot + heatmap_plot +
```

```
  plot_layout(widths = c(1, 1.2))    # Increase the width ratio of heatmap
# Display combination diagram
print(combined_plot)

# 3. Save the graph
ggsave("plantcare_composite_plot.pdf", combined_plot,
       width = 22, height = 16, limitsize = FALSE)

ggsave("plantcare_composite_plot.jpg", combined_plot,
       width = 22, height = 16, dpi = 300, limitsize = FALSE)

# 4. Show statistics
cat("Gene dosage:", length(unique(plantCARE_HeatmapPlot$id)), "\n")
cat("Elements num:", length(unique(plantCARE_HeatmapPlot$category)), "\n")
cat("Description category:", paste(unique(plantCARE_HeatmapPlot$Description),
collapse = ", "), "\n\n")

# Statistics by category
category_summary <- plantCARE_HeatmapPlot %>%
  group_by(Description) %>%
  summarise(
    unique_elements = n_distinct(category),
    total_occurrences = sum(category_num),
    avg_per_gene = round(total_occurrences /
length(unique(plantCARE_HeatmapPlot$id)), 2),
    .groups = 'drop'
  )
print("Statistics by category:")
print(category_summary)
```
