# Supplementary material for: Genome-wide identification and expression profiling of DnaJ gene family in Gossypium barbadense reveals candidate thermotolerance genes
Source: Front Plant Sci. 2026 Jan 20;16:1728216. doi: 10.3389/fpls.2025.1728216 (PMC12865410; doi:10.3389/fpls.2025.1728216)
Supplement: Supplementary Data Sheet 1 — Protein sequences of the GbdnaJ gene family. [file Supplementaryfile1.zip › Supplementary Material/Data Sheet 3.PDF]

```{r}
library(pheatmap)
library(tibble)
library(ggplot2)

dev.new()
library(RColorBrewer)
Gb40_pheatmap <- pheatmap(
  log2(Gb40_de + 1),
  scale = "row",
  # Use the RdYlBu color scheme and reverse it.
  color = colorRampPalette(rev(brewer.pal(11, "RdYlBu")))(100),
  show_rownames = TRUE,
  show_colnames = TRUE,
  fontsize_row = 6,
  fontsize_col = 8,
  cellwidth = 10,
  cellheight = 5,
  cluster_rows = TRUE,
  cluster_cols = TRUE,
  clustering_distance_rows = "euclidean",
  clustering_distance_cols = "euclidean",
  clustering_method = "complete",
  border_color = NA,
  treeheight_row = 30,
  treeheight_col = 30,
  main = "GbDnaJs expression thermograms",
  filename = "Gb40_heatmap_orange.pdf",
  width = 12,
  height = 15
)
```

```{r}
# # Adjust the order of line names
# Define the column order
desired_column_order <- c("CK_Flower1", "CK_Flower2", "CK_Flower3",
                          "CK_Bud1", "CK_Bud2", "CK_Bud3",
                          "CK_Leaf1", "CK_Leaf2", "CK_Leaf3",
                          "Heat_Flower1", "Heat_Flower2", "Heat_Flower3",
                          "Heat_Bud1", "Heat_Bud2", "Heat_Bud3",
                          "Heat_Leaf1", "Heat_Leaf2", "Heat_Leaf3")

# Ensure that all specified column names exist in the data.
```

~~~
existing_columns <- intersect(desired_column_order, colnames(Gb40_de))
missing_columns <- setdiff(desired_column_order, colnames(Gb40_de))

if (length(missing_columns) > 0) {
  warning(paste("The following name does not exist in the data:", paste(missing_columns, collapse = ", ")))
}

# Rearrange the column order of data frames
Gb40_de_ordered <- Gb40_de[, existing_columns, drop = FALSE]

# Create a heat map and save it as a PDF
library(RColorBrewer)
library(pheatmap)

pheatmap(
  log2(Gb40_de_ordered + 1),
  scale = "row",
  color = colorRampPalette(rev(brewer.pal(11, "RdYlBu")))(100),
  show_rownames = TRUE,
  show_colnames = TRUE,
  fontsize_row = 6,
  fontsize_col = 8,
  cellwidth = 10,
  cellheight = 5,
  cluster_rows = TRUE,
  cluster_cols = FALSE,    # Turn off column clustering and use the specified column order
  clustering_distance_rows = "euclidean",
  clustering_method = "complete",
  border_color = NA,
  treeheight_row = 30,
  treeheight_col = 30,
  main = "GbDnaJs expression thermograms",
  filename = "Gb40_heatmap_orange_2.pdf",
  width = 12,
  height = 15,
)
```


```{r}
# Save heat map
pdf("GbDnaJs_pheatmap.pdf", width = 10, height = 12)
```
~~~
