# Supplementary material for: Genome-wide identification and expression profiling of DnaJ gene family in Gossypium barbadense reveals candidate thermotolerance genes
Source: Front Plant Sci. 2026 Jan 20;16:1728216. doi: 10.3389/fpls.2025.1728216 (PMC12865410; doi:10.3389/fpls.2025.1728216)
Supplement: Supplementary Data Sheet 1 — Protein sequences of the GbdnaJ gene family. [file Supplementaryfile1.zip › Supplementary Material/Table 1.DOCX]

**Supplementary Table. 2 Primer sequences used for qRT-PCR analysis of *GbDnaJ*.** The table includes gene identifiers, primer forward and reverse sequences, amplicon size (bp), GC content (%GC) and melting temperature (Tm).

| **Primer** | **Sequence(5' to 3')** | **(bp)** | **%GC** | **Tm** |
| --- | --- | --- | --- | --- |
| Gh-ubq7-F | GAAGGCATTCCACCTGACCAAC | 22 | 54.5 | 59 |
| Gh-ubq7-R | CTTGACCTTCTTCTTCTTGTGCTTG | 25 | 44 | 56 |
| GbDnaJ1-F | AGCGGTTTATACGGGTCCAG | 20 | 55 | 57.2 |
| GbDnaJ1-R | CGTCGTCTAGCAGAGAAAGCA | 21 | 52.4 | 57 |
| GbDnaJ3-F | AGCTCGAAACGGTGGAACAT | 20 | 50 | 56.9 |
| GbDnaJ3-R | CTCCCAATGCCCTGAGTGAG | 20 | 60 | 58.5 |
| GbDnaJ21-F | GCGAAGCACAACGACTTTCA | 20 | 50 | 56.3 |
| GbDnaJ21-R | ATACCGCTTGTCTTTGCCGA | 20 | 50 | 56.9 |
| GbDnaJ28-F | GATACGCCAGAAACCCAGGA | 20 | 55 | 57.2 |
| GbDnaJ28-R | CCGGCGTCATACATTGATCG | 20 | 55 | 56.5 |
| GbDnaJ36-F | CAACCGAACACAATCCCTGC | 20 | 55 | 57.2 |
| GbDnaJ36-R | CTGTTAATGCTTCGGCCAGC | 20 | 55 | 56.3 |
| GbDnaJ47-F | CCCACGGCGGATCTTGTTAT | 20 | 55 | 57.8 |
| GbDnaJ47-R | TGGCGTATCTGTCTGGATGC | 20 | 55 | 57.5 |
| GbDnaJ56-F | ATTACCTCAACGCAAGGCGA | 20 | 50 | 56.9 |
| GbDnaJ56-R | TCACGCTTTTCGGGATCAGT | 20 | 50 | 56.6 |
| GbDnaJ57-F | GGTTAAGGCACTTGAGGCCA | 20 | 55 | 58.0 |
| GbDnaJ57-R | GCGTAGTCTCCTCACACTCG | 20 | 60 | 57.9 |
| GbDnaJ59-F | AGACAAAGGCGGTGATCCTG | 20 | 55 | 57.7 |
| GbDnaJ59-R | CCTTGAGTGCATCCTCACCA | 20 | 55 | 57.5 |
| GbDnaJ60-F | GGCCTTACTCCTTCGAGGTC | 20 | 60 | 57.9 |
| GbDnaJ60-R | AGGCGGAGACCTTTCTGGTA | 20 | 55 | 58.2 |
| GbDnaJ63-F | GCTTGCTCTGAAATGGCACC | 20 | 55 | 57.6 |
| GbDnaJ63-R | GGATCGTAGAGACTGGCGTC | 20 | 60 | 57.8 |
| GbDnaJ67-F | ACGTTGCCTTGTAGCCTTGA | 20 | 50 | 56.9 |
| GbDnaJ67-R | CTGGCAATGTCTTCCCGCTA | 20 | 55 | 57.7 |
| GbDnaJ81-F | CATTGTTCGCCCACTTCAGC | 20 | 55 | 57.4 |
| GbDnaJ81-R | ACCCATCTCTTGCTTCGTCC | 20 | 55 | 57.5 |
| GbDnaJ101-F | CCAAGGGTACAAGAAGGCGT | 20 | 55 | 57.7 |
| GbDnaJ101-R | GCATTCTTCGCCTACCACCT | 20 | 55 | 57.8 |
| GbDnaJ105-F | CGTTCTCTCAAACGGGTCCA | 20 | 55 | 57.5 |
| GbDnaJ105-R | CCTCCAAACTAACCCCCTCG | 20 | 60 | 58.2 |
